# Discrimination of missing data types in metabolomics data based on particle swarm optimization algorithm and XGBoost model

**DOI:** 10.1038/s41598-023-50646-8

**Published:** 2024-01-02

**Authors:** Yang Yuan, Jianqiang Du, Jigen Luo, Yanchen Zhu, Qiang Huang, Mengting Zhang

**Affiliations:** 1 School of Computer Science, Jiangxi University of Chinese Medicine, Nanchang, 330004 China; 2 Key Laboratory of Artificial Intelligence in Chinese Medicine, Jiangxi University of Chinese Medicine, Nanchang, 330004 China

**Keywords:** Computational biology and bioinformatics, Data processing

## Abstract

In the field of data analysis, it is often faced with a large number of missing values, especially in metabolomics data, this problem is more prominent. Data imputation is a common method to deal with missing metabolomics data, while traditional data imputation methods usually ignore the differences in missing types, and thus the results of data imputation are not satisfactory. In order to discriminate the missing types of metabolomics data, a missing data classification model (PX-MDC) based on particle swarm algorithm and XGBoost is proposed in this paper. First, the missing values in a given missing data set are obtained by panning the missing values to obtain the largest subset of complete data, and then the particle swarm algorithm is used to search for the concentration threshold of missing data and the proportion of low concentration deletions as a percentage of overall deletions. Next, the missing data are simulated based on the search results. Finally, the training data are trained using the XGBoost model using the feature set proposed in this paper in order to build a classifier for the missing data. The experimental results show that the particle swarm algorithm is able to match the traditional enumeration method in terms of accuracy and significantly reduce the search time in concentration threshold search. Compared with the current mainstream methods, the PX-MDC model designed in this paper exhibits higher accuracy and is able to distinguish different deletion types for the same metabolite. This study is expected to make an important breakthrough in metabolomics data imputation and provide strong support for research in related fields.

## Introduction

Metabolomics aims to provide a panoramic view of the metabolome, revealing the metabolic state of an organism in different physiological or pathological states by analyzing and interpreting metabolite profiles in biological samples^1^. Metabolomics can be applied to multiple fields, including life sciences, medicine, agriculture, and environmental science, among others. Through the study of metabolomics data, it is possible to gain in-depth insights into changes in metabolic reactions within organisms, discover potential biomarkers, identify key enzymes in metabolic pathways, and explore the underlying mechanisms of diseases^2,3^.

Metabolomics data are of high dimensionality, diversity and complexity, encompassing information on a large number of metabolites. These data are often characterized as highly dynamic and are modulated by multiple factors, including environmental, genetic and physiological states^4–6^. There are often missing values in metabolomics data^7^, which need to be processed in an appropriate way to fully utilize the available data for data analysis. Therefore, imputation or completion of missing values in metabolomics data is a very necessary preprocessing task.

There are various reasons for missing metabolomics data, specifically the following three^8^: (1) metabolite concentrations are below the instrumental detection threshold; (2) missing values may be introduced randomly; and (3) changes in the measurement environment may result in missingness. Based on these reasons, missing values of data can be categorized into three types^9–12^: (1) Missing Completely At Random (MCAR: Missing Completely At Random), which means that missing data are completely random and have no relation to the missing variable and other variables; (2) Missing At Random (MAR: Missing At Random), MAR values are independent of missing values but are dependent on the observed values (e.g., measured hormone variable); (3) Missing Not At Random (MNAR: Missing Not At Random), MNAR values refer to missing data depending on non-observed variables, where concentrations below a specific threshold cannot be detected due to the limited detection accuracy of the instrument, resulting in missing data.

Due to different types of missingness, different imputation methods need to be selected. Identifying the type of missing values helps in selecting the appropriate data imputation mechanism. If the missing data are missing completely at random (MCAR), the probability of missingness can be assumed to be independent of the observed data itself or other unobserved factors. In this case, multiple imputation methods, such as multiple imputation, can be used^13^. Because it is often difficult to determine whether there is a link between missing values, it is common to treat missing at random (MAR) as missing completely at random (MCAR) and use the same imputation method for them^14^. However, if the missing data are missing at random (MNAR), it implies that the missing values themselves may be related to unobserved variables and that the missing values should be below the minimum value of the metabolite concentration. In such cases, imputation methods that simply use MCAR assumptions may result in biased or inaccurate data. Therefore, advanced imputation methods such as maximum likelihood estimation can be established for MNAR type missingness^9^. Different imputation methods come with different assumptions and limitations. Accurately identifying the type of missing data can help avoid incorrect assumptions and inappropriate imputation methods, thereby enhancing the accuracy and reliability of data imputation. Dekermanjian et al. proposed a unique two-step imputation method^15^, with the first step focusing on identifying the type of missing values. However, this method has a relatively low accuracy and assumes that there is only one type of missing data for the same metabolite. In response to this challenge, the paper introduces the based on Particle Swarm Optimization and XGBoost Missing Data Classification model (PX-MDC).

In this research, this paper focuses on improving the classifier algorithm proposed by Dekermanjian et al. in order to remedy its shortcomings. The approximate steps of the classifier algorithm proposed by Dekermanjian et al. are as follows: first, the missing dataset is panned, the concentration thresholds of the missing data are searched through the grid, and then seven features, namely, mean, median, minimum, maximum, missing rate, metabolite quartile, and metabolite abundance of metabolites, are selected to construct the classifier using the random forest model. By studying the method in depth, this paper identifies the shortcomings of the method in using a grid search to determine concentration thresholds and the proportion of low-concentration deletions as a percentage of the overall deletions when the search granularity is too large. Grid search is essentially a limited parameter enumeration method that is empirically related and cannot fully represent the diversity of all data. In addition, in terms of feature construction, this paper also notes that Dekermanjian et al. method treats different deletion types for the same metabolite as identical, which may lead to loss of information. In terms of selecting classifiers, this paper also observes that random forests may not be the best choice for the problem of categorizing deletion types in metabolomics data. In order to solve the above problems, three key improvements are proposed in this paper:

- using particle swarm algorithm to search for concentration thresholds and the proportion of low concentration deletions as a percentage of overall deletions to improve search efficiency and applicability.
- Nine different features, including the number of consecutively missing metabolites, the number of consecutively missing samples, the mean, the median, the minimum, the maximum, the missing rate of metabolites, the name of metabolites, and the relationship between metabolites and the high and low concentrations they belong to, were used to construct the classifiers.
- Introduction of XGBoost model as a construction method for the classifiers.

The results show that the method proposed in this paper has faster speed and wider applicability in searching concentration thresholds and the proportion of low concentration deletions as a percentage of overall deletions. Compared with Dekermanjian et al. method, the features constructed in this paper are able to distinguish different deletion types of the same metabolite. In addition, the classifier constructed using the XGBoost model exhibits higher classification accuracy. These improvements provide a more effective and accurate solution to the problem of categorizing deletion types in metabolomics data, which is expected to have a significant impact in related fields.

### Data simulation

In real metabolomics data, the missing types of missing values are usually difficult to obtain, and therefore it is not possible to verify the accuracy of classifiers for different types of missing data. In this paper, simulation of missing metabolomics data is needed to generate missing data containing labels of missing types. First, a complete dataset is selected and missing data are introduced according to the Mixed missingness (MM) method^14^ proposed by Y. Lee et al. to obtain missing data $X^{MM}$. In this process, the type of missing data is recorded for subsequent verification of the accuracy of the classifier.

### Datasets

Three publicly available and complete datasets were selected for this data imputation experiment, all of which are accessible through the Metabolomics Workbench (https://www.metabolomicsworkbench.org/).

### Protein family data (bacteria)

This dataset is from an experiment to examine differences in intracellular metabolite concentrations between wild-type and knockout strains. The study consisted of 5 groups of 6 replicates each, with 1 sample missing (29 samples total), and 249 metabolites were determined by GC–MS. The dataset and its experimental acquisition details are described in study ID ST000118.

### Asthma/obesity correlation study (mouse)

The experiments in this dataset examined how three different diets affect arginine metabolic pathways in mouse lungs. The experiment contained 80 mouse lung tissue samples with 612 metabolites measured by GC–MS, and details of this dataset and its experimental collection are available in study ID ST000419.

### Non-diabetic/diabetic correlation studies (human)

The experiments in this dataset investigated changes in glucose homeostasis in two different populations. The experiment contained 181 samples with 511 metabolites determined by GC–MS, and details of the dataset and its experimental collection are provided in study ID ST000385.

### Generating missing data

**Figure 1:**
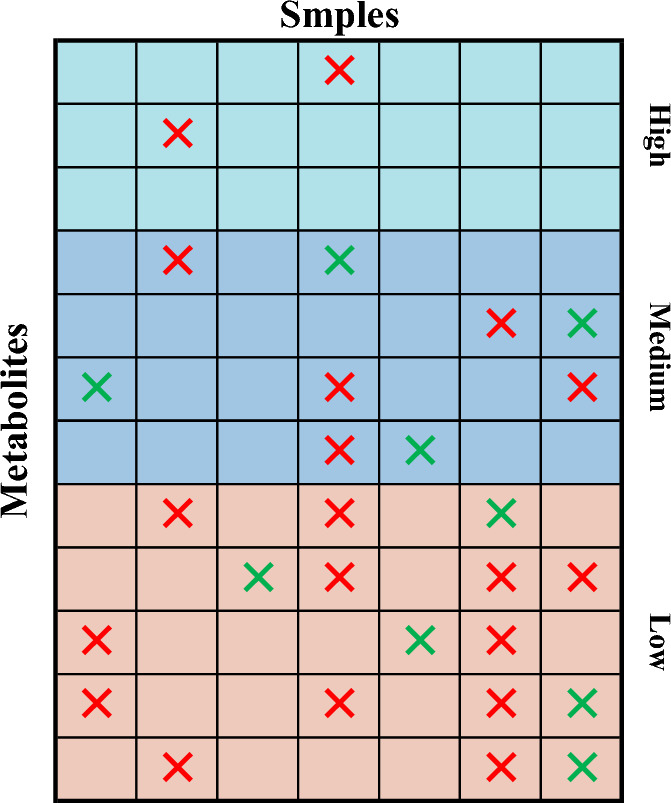
Schematic diagram of MM mixed deletion. Each column represents a sample, while each row represents a metabolite. In the figure, red color is used in this paper to indicate MNAR missing, while green color indicates MCAR missing.

There are three main types of common metabolomics data missingness, but in real missing data, there is usually a mixture of multiple missingness types. The Mixed missingness (MM) model proposed by Lee et al. more realistically reflects the distribution of missingness in metabolomics datasets. In the MM model, the concentrations of metabolites are sorted in order from high to low, and a threshold percentage is set to classify the metabolites into high, medium, and low concentration levels. Threshold percentages need to be set by themselves according to the data. By observing the data, this paper assumes that the metabolite concentration higher than the overall metabolite concentration average is high concentration, lower than 1/10 of the overall metabolite concentration average is low concentration, and the rest of the metabolite is medium concentration, for three different data, the assumption can make the concentration thresholds are not the same, which can avoid the result error caused by the concentration thresholds are the same. The MM model points out that the missing rate of the low concentration accounts for the vast majority of the overall missing rate. Assuming that $\alpha$ is the proportion of low-concentration deletions to overall deletions. The range of $\alpha$ is set from 40 to 60% in this paper’s experiments, with a step size of 5%. The missing rate of the data ranges from 2.5 to 40% with a step size of 2.5%. With this setup, real metabolomics data with a mixture of different deletion rates and deletion types can be simulated. In fact, whatever assumptions are made about the concentration threshold and the proportion of low concentration misses to overall misses do not have an impact on the results of this paper's use of particle swarm search for concentration thresholds. This is because the particle swarm search algorithm used in this paper is characterized by searching for a better global optimal solution, thus adapting to different contexts. Figure 1 clearly represents the distribution of the MM model.

## Methods

**Figure 2:**
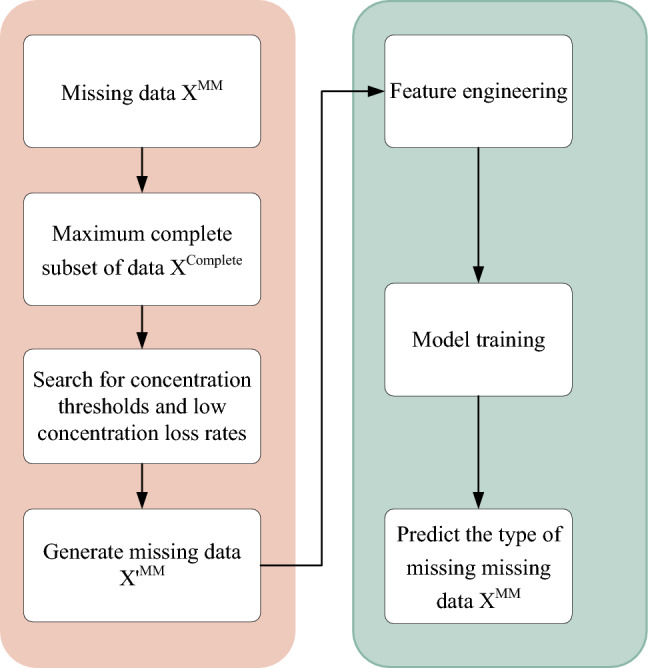
The flowchart of the PX-MDC algorithm proposed in this paper shows the process of constructing the training data on the left side, while the right side shows the steps of constructing the classifier from the training data.

The overall process of constructing a classifier proposed in this paper is as follows: firstly, a maximum complete set $X^{Complete}$ is obtained based on the missing data $X^{MM}$. Secondly, $X^{Complete}$ is subjected to different concentration thresholds to simulate the missing to obtain $X{\prime }^{MM}$. A particle swarm algorithm is utilized to find the $X{\prime }^{MM}$ that has the smallest Euclidean distance from $X^{MM}$. At this time, the concentration threshold and the proportion of low concentration deletions as a percentage of overall deletions corresponding to $X{\prime }^{MM}$ are the concentration threshold and the proportion of low concentration deletions as a percentage of overall deletions of $X^{MM}$. Finally, $X{\prime }^{MM}$ is utilized to construct a classifier to predict the missing data types in $X^{MM}$. Figure 2 depicts the method proposed in this paper.

### Maximum complete subset of missing data

**Figure 3:**
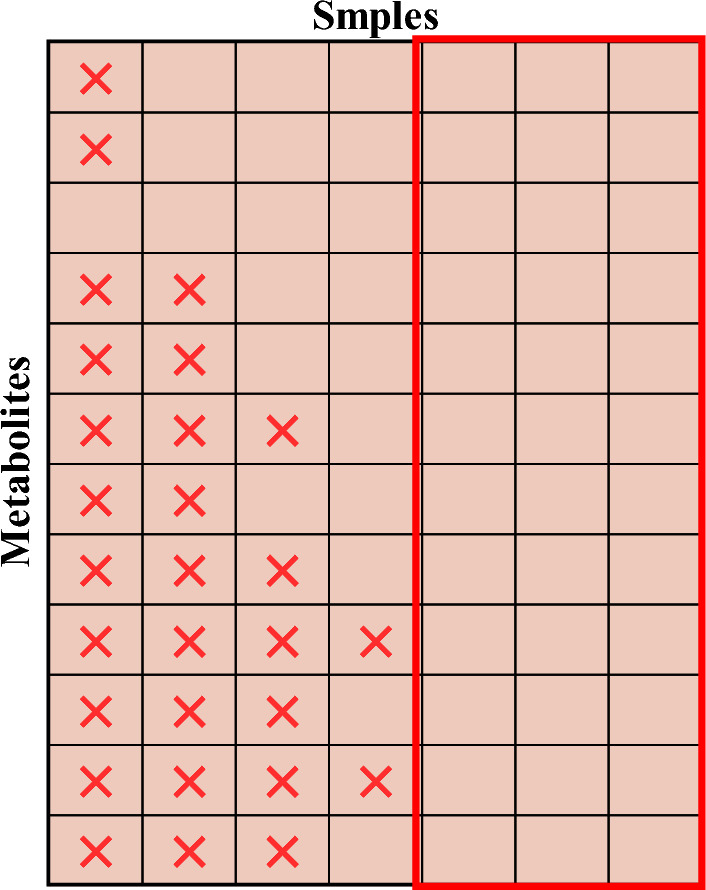
By panning the missing data, it is possible to obtain the largest complete subset of the red region as shown in the figure.

In order to construct a data missing type classifier, a panning operation on the missing data is required. In this paper, the missing data for each metabolite was flattened to the far left to obtain the largest subset of complete data. This ensures that as much of the available data as possible is utilized when constructing the classifier and reduces the impact of missing data on the performance of the classifier. The red boxed area in Fig. 3 represents the largest complete subset of this dataset $X^{Complete}$. When the sample size in $X^{Complete}$ is small, metabolites with missing values exceeding 20% of the sample size can be removed according to the 80% rule^16^ for missing value processing of metabolomics data, and such processing can solve the problem of small sample size in $X^{Complete}$.

### Particle swarm search

**Figure 4:**
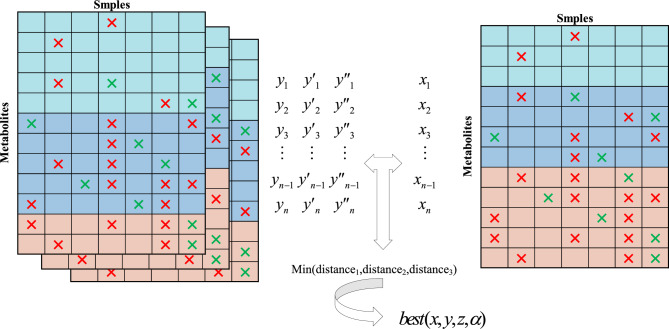
For each set of $(x,y,z,\alpha )$, a copy of the missing data can be generated using the MM algorithm. By calculating the missing vectors of these missing data, the Euclidean distance between the two copies of data can be obtained. The smaller the distance, the more similar these two pieces of data are. By particle swarm search algorithm, the optimal solution can be found.

In order to model data that is consistent with the distribution of missing data, it is necessary to obtain a concentration threshold for the original data and the proportion of low concentration deletions as a percentage of overall deletions. Assume that the threshold for high concentration is $x$, the threshold for medium concentration is $y$, the threshold for low concentration is $z$, and the he proportion of low concentration deletions to overall deletions is $\alpha$. With the MM algorithm, a copy of the missing data can be generated and the similarity between this generated data and the original missing data can be calculated. In order to determine the best combination of concentration threshold and the proportion of low concentration deletions as a percentage of overall deletions $(x,y,z,\alpha )$, the similarity between each copy of the generated missing data and the original missing data can be calculated^15^. This is done by calculating the deletion rate for each metabolite and transforming it into a vector of deletion rates. Then, the Euclidean distance between the two missing rate vectors is calculated, and a smaller distance indicates a higher degree of similarity between the data^17^. Considering the randomness of the MM algorithm, this paper generates 10 copies of missing data for each group $(x,y,z,\alpha )$. For each copy of the generated missing data and the original missing data, the Euclidean distances between them are calculated, and the arithmetic mean of these distances is taken as the Euclidean distance corresponding to the missing data generated for that group $(x,y,z,\alpha )$. This can reduce the impact of randomness in the MM algorithm and increase the stability of the results. Figure 4 shows the schematic diagram of concentration threshold search.

For the search of concentration thresholds, Dekermanjian et al. proposes to use a grid search to achieve this, with the grid defining $x$ from 5 to 100%, $y$ from 60 to 80%, $\alpha$ from 5 to 60%, and all with a search step of 5%. In this paper, it is argued that the concentration thresholds are different for each set of data, the range of thresholds should not be fixed empirically, and a step size of 5% may lead to a large error in the results. Particle Swarm Optimization^18–20^ (PSO) is a heuristic global optimization algorithm: first, a population of particles is initialized, each representing a potential solution, which are randomly distributed in the problem space. Then, the fitness value of each particle, i.e., the value of the objective function of the problem at the current position, is computed. Each particle maintains its own individual best position and the global best position of the whole swarm of particles. In each iteration, the particles are continuously updated according to their velocity and position, with the update of the velocity being influenced by the attractiveness of the individual best position and the global best position. This process iterates until a set number of iterations or a stopping condition is reached, and finally the solution corresponding to the global best position is returned as the optimal or near-optimal solution of the problem. Particle swarm search algorithms help to search and gradually approach the optimal solution in the problem space by modeling the synergy and competition among individuals in the population. Therefore, the particle swarm algorithm is a flexible and efficient global optimization algorithm that can effectively search the solution space for the optimal solution or near-optimal solution^21–23^, which is suitable for the search problem of the concentration threshold of this problem. In order to compare the results of particle swarm search, this paper also carries out enumeration of all the $(x,y,z,\alpha )$ that satisfy the conditions to obtain the optimal solution. Following are the steps to implement the particle swarm search concentration threshold in this paper:

1. Initialize the particle population, randomly initialize the position and velocity for each particle.
2. For each group of $(x,y,z,\alpha )$, perform MM algorithm on $X^{Complete}$ to obtain $X{\prime }^{MM}$.
3. Calculate the deletion rate of each metabolite in $X^{MM}$ and $X{\prime }^{MM}$ to get the corresponding deletion vector.
4. Calculate the Euclidean distance between the two missing vectors in step 3 and store it.
5. loop steps 2–4 ten times and use the average of the ten Euclidean distances as the corresponding Euclidean distance of $(x,y,z,\alpha )$.
6. Update the position of the particle swarm to get the new $(x,y,z,\alpha )$, and jump back to step 2 until the stopping condition of the particle swarm search is met.
7. Obtain the optimal particle swarm.

### Classifier features

The optimal concentration threshold and the proportion of low concentration deletions as a percentage of overall deletions can be obtained by particle swarm search algorithm. Using these parameters, MM missing is performed on the maximum complete subset $X^{Complete}$ to obtain $X{\prime }^{MM}$, and the type of missing data is recorded so that it can be used to train the classifier. In this paper, the following features are proposed to construct a classifier for a particular missing value:

- Number of consecutive missing metabolites: when a metabolite has a high number of consecutive missing metabolites in the dataset, this may indicate that the missingness is not random, but more likely due to unobservable factors.
- Number of consecutive missing samples: in metabolomics experiments, samples are usually arranged in increasing or decreasing order of concentration. When the concentration reaches a specific value, the deletion of metabolites may change, thus affecting the pattern of deletions in the data.
- Maximum, minimum, median, mean, and deletion rate of metabolites for each metabolite: statistical characterization of a metabolite provides a better understanding of the nature of that metabolite and the pattern of deletions.
- Metabolite name: Different metabolites may be affected by different biological or experimental conditions, resulting in different deletion patterns.
- High or low concentration of the metabolite to which it belongs: metabolites at different concentration levels may have different deletion patterns.

**Figure 5:**
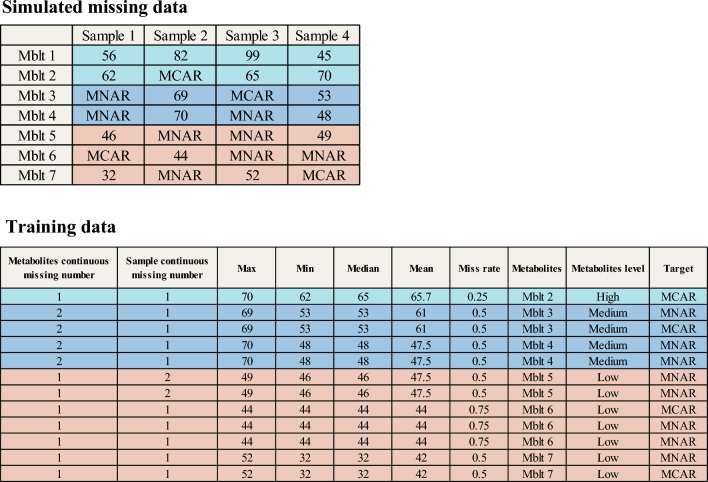
Example plot of training data. In the upper table, the missing data is shown, where the rows represent different metabolites, the columns represent different samples, and the colors indicate the metabolites relative to the concentration threshold. In the following table, the training data obtained after incorporating the feature construction method proposed herein.

Each missing value in $X{\prime }^{MM}$ is feature-labeled to obtain the corresponding vector, and when all the missing values are vectorized, the training data corresponding to that group of data is obtained, where Fig. 5 clearly depicts a copy of the training data corresponding to the classifier for the missing data. Since each group of $(x,y,z,\alpha )$ Y has random missing differences in $X{\prime }^{MM}$ generated each time, several copies of $X{\prime }^{MM}$ are generated to get richer training data to improve the accuracy of the classifier. According to the size of the amount of missing data, in this paper, we choose to generate $X{\prime }^{MM}$ five times to get the corresponding training data.

### Classifier model

Common classifier models include plain Bayes, support vector machines, decision trees, random forests and neural networks^24,25^. In Dekermanjian et al. study, random forest was used as the model to train the classifier. And in this paper, four common models, Random Forest^26–28^, Plain Bayes^29,30^, XGBoost^31–33^ and BP Neural Network^34,35^ are compared for training.

- Random Forest (RF): Random Forest is a powerful machine learning method for classification by constructing multiple decision trees and integrating their predictions. Random Forest uses self-sampling and random selection of feature subsets to increase the variability among decision trees and improve the generalization ability of the overall model. It is well adapted to high-dimensional and large-scale datasets, can handle missing values and unbalanced data, and can assess the importance of variables and reduce the risk of overfitting with high accuracy and robustness, and is widely used in real-world scenarios.
- Naive Bayes: Naive Bayes is a classification method based on Bayes' theorem. It is based on the assumption of conditional independence of features and classifies new samples by learning a priori probability and conditional probability models. Plain Bayes counts the frequency of each feature under each category in the training phase, calculates the posterior probability using Bayes' theorem in the prediction phase, and selects the category with the largest posterior probability as the prediction result. Plain Bayes has good classification effect on small sample data, scalability on high-dimensional feature data, some robustness to missing data, and fast training and prediction speed. Although its assumption of conditional independence of features does not exactly match the actual situation, Park Bayes still shows good classification performance and is a simple but effective classification method for a variety of practical problems.
- XGBoost: XGBoost is an integrated learning algorithm based on gradient boosting decision trees, which is widely used in classification problems. It continuously improves the prediction performance of the model by iteratively training the decision tree model and optimizing the loss function. XGBoost uses an adaptive learning strategy to adjust the sample weights according to the prediction errors of the previous model and pays more attention to the samples that have been previously incorrectly predicted. At the same time, the complexity of the model is controlled by regularization terms to avoid overfitting. XGBoost has the advantages of handling high-dimensional sparse data, being robust to missing values, and evaluating feature importance. It excels in handling large-scale datasets and complex tasks and is one of the important classification algorithms in machine learning.
- BP Neural Network (Backpropagation Neural Network): BP Neural Network is an artificial neural network based on the backpropagation algorithm, which is commonly used in classification problems. It consists of an input layer, a hidden layer and an output layer, and realizes the classification of new samples by learning the mapping relationship between the input samples and the corresponding output labels. BP neural network calculates the output through forward propagation, and adjusts the weights and biases in the network according to the prediction error by using the backpropagation algorithm. By updating the weights and biases through many iterations, the network gradually optimizes the classification performance. BP neural networks have the advantages of learning nonlinear mappings, strong adaptability, and good generalization ability. However, BP neural networks also have the problems of local optimal solutions and sensitivity to initial weights. Nevertheless, BP neural network is still an important classification algorithm and is widely used in various fields.

## Results

### Particle swarm algorithm evaluation

In the problem of searching for the concentration threshold of missing data and the proportion of low concentration deletions as a percentage of overall deletions, Dekermanjian et al. proposed grid search to solve this problem, the essence of grid search is to enumerate the finite solution, and the initialization of grid search is based on experience, and can not be applied to all the data, so in this paper, we propose to use the Particle Swarm Algorithm to solve this problem. In order to verify the reliability of the results of the particle swarm algorithm search, in this paper, the enumeration method is used to solve the threshold, that is, $(x,y,z,\alpha )$ are from 1 to 100%, the step size is 1%, and $x + y + z = 100\%$ satisfy , the results of the enumeration method and the results of the Particle Swarm Algorithm are compared.

**Figure 6:**
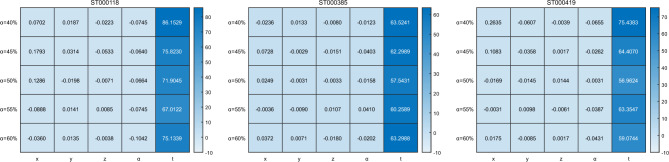
The search results of the particle swarm algorithm and the enumeration method were compared. The formula for calculating the percentage difference is the absolute value of the difference between the particle swarm results and the enumeration results divided by the particle swarm results. The heatmap clearly shows that the search time t has a greater impact than the difference.

For each set of data, by applying the particle swarm algorithm and the enumeration method, their thresholds $x,y,z,\alpha$ and the search time $t$ were obtained so that the percentage difference between the two algorithms could be calculated. For data with the same $\alpha$ value but different missing rates, there is consistency in their concentration thresholds and the proportion of low concentration deletions as a percentage of overall deletions. Therefore, such data can be categorized into the same category. When comparing the results, focusing on their mean values, this practice can provide support to verify the stability of the algorithm. From Fig. 6, it can be observed that the maximum percentage difference of $x,y,z,\alpha$ for all the data is 0.26, while the minimum percentage difference of the search time $t$ is 59.07. From this, it can be concluded that the differences between $x,y,z,\alpha$ are very small and are within the acceptable error range, but the difference of the search time $t$ is very obvious, and the enumeration method takes much longer than the particle swarm algorithm. This indicates that the particle swarm algorithm not only achieves comparable quality of results to the enumeration method, but also reduces the solution time significantly and is suitable for this problem.

### Feature quality and PX-MDC model evaluation

In order to evaluate the quality of features and PX-MDC model proposed in this paper, the experimental results of the proposed classifier model by Dekermanjian et al. as well as the classifiers such as Random Forest (rf), Plain Bayes, and BP Neural Networks are compared. The MM algorithm is applied to generate 10 batches of datasets with each particular value. For each batch of data, experiments were conducted with the missing rate gradually increasing from 2.5 to 40% (in steps of 2.5%) and the missing type of the missing value in each missing data was recorded. The types of missing values in these 10 batches of data were predicted using the constructed classifiers, and the prediction accuracies in the validation set and in the validation set with different missing rates were obtained, and the stability of the algorithms was solidly supported by averaging the prediction accuracies at the same missing rate.

**Figure 7:**
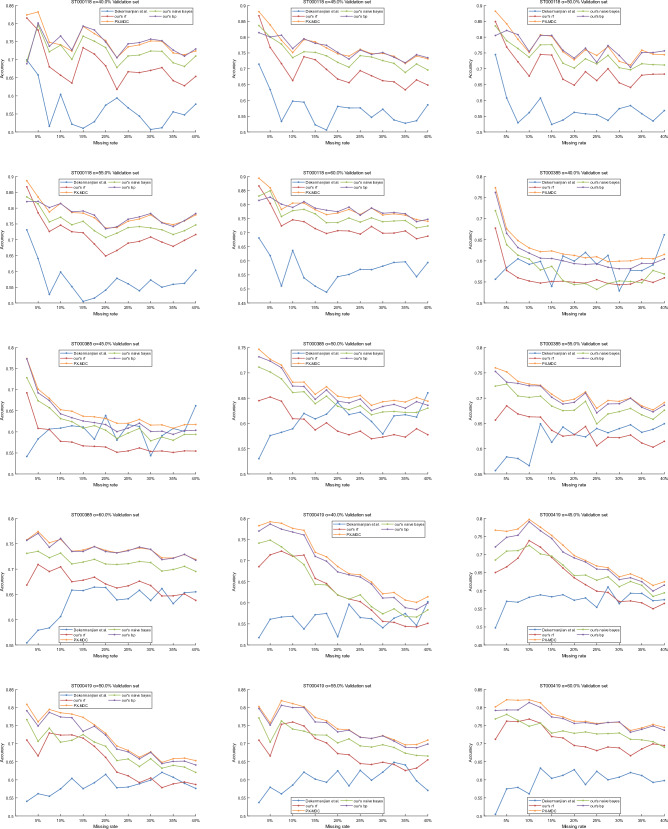
Accuracy of the classifier on the validation set. In this paper, the training data is divided in the ratio of 8:2 where 80% is used to construct the training set while 20% is used for the validation set. This study compares the experimental results of PX-MDC with Dekermanjian et al., Random Forest (rf), Plain Bayes, and BP Neural Networks on different combinations of data.

**Figure 8:**
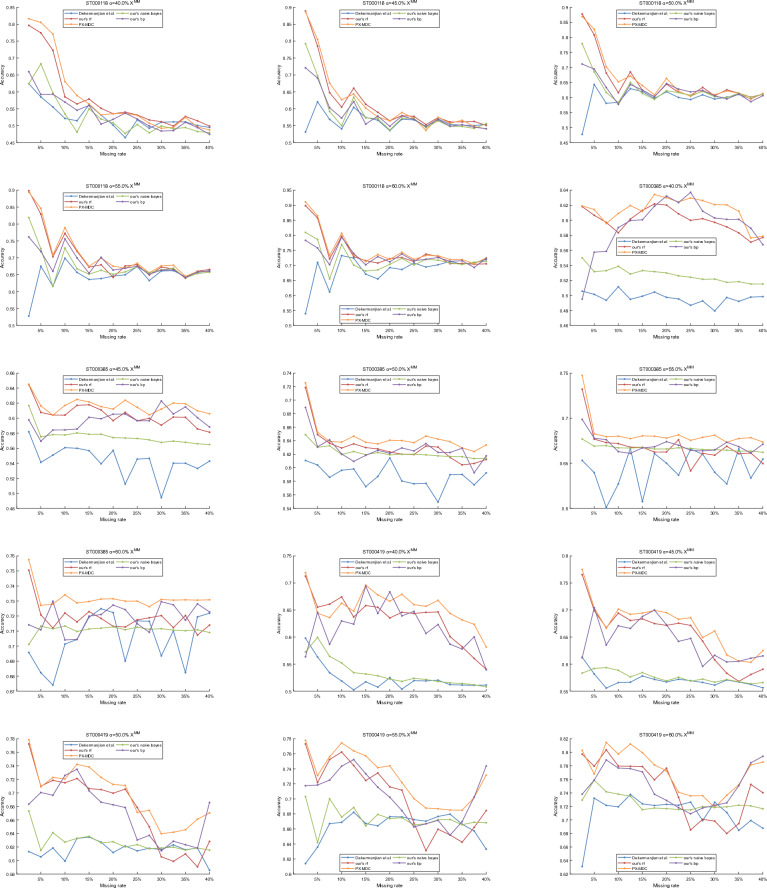
Accuracy of classifiers on missing datasets. This study compares the experimental results of PX-MDC with Dekermanjian et al., Random Forest (rf), Plain Bayes, and BP Neural Networks on different combinations of data.

According to the results in Figs. 7 and 8, the method proposed by Dekermanjian et al. performs relatively poorly on the validation set and the missing data set compared to the four models proposed in this paper. On the validation set, the average accuracy of the method proposed in this paper using the random forest model is 13% higher on the ST000118 dataset and 8% higher on the ST000419 dataset relative to the method proposed by Dekermanjian et al. Meanwhile, the PX-MDC model has 20% higher average accuracy on the ST000118 dataset, 6% higher on the ST000385 dataset, and 14% higher on the ST000419 dataset relative to the method proposed by Dekermanjian et al. On the missing dataset, the method proposed in this paper using the random forest model has 6% higher average accuracy on the ST000118 dataset, 5% higher on the ST000385 dataset, and 7% higher on the ST000419 dataset, relative to the method proposed by Dekermanjian et al. The PX-MDC model is also more accurate on the ST000118 dataset, ST000385 dataset, and ST000419 dataset. Meanwhile, the average accuracy of the PX-MDC model relative to the method proposed by Dekermanjian et al. is 6% higher on the ST000118 dataset, 6% higher on the ST000385 dataset, and 9% higher on the ST000419 dataset.

In order to test the quality of the features proposed in this paper, the two features proposed in this paper were added separately were compared and the experimental results (Supplementary Figs. 1–8) showed that the accuracy of the classifier was better when both features were considered. Considering the effect of biological or experimental variables, comparing the name of the metabolite and the concentration level, the experimental results (Supplementary Figs. 9–10) show that the accuracy of the classifier is better when considering the name of the metabolite and the concentration level. In order to verify the effect of the division of concentration thresholds on the experimental results, three different sets of concentration thresholds were randomly set, and the comparison found that the accuracy of the PX-MDC model has a certain stability (Supplementary Tables 1–10).

Therefore, it can be concluded that the method proposed in this paper is superior to the method proposed by Dekermanjian et al. in terms of feature quality. Further comparative studies also show that the XGBoost model is a more suitable choice for the classification of metabolomics missing data types. In the field of metabolomics data analysis, it is common to exclude the same metabolite when its missing rate reaches 20%, which follows the “80% principle” of metabolomics data processing. According to the results in Figs. 7 and 8, the PX-MDC model performs better in terms of accuracy than the method proposed by Dekermanjian et al. when the deletion rate is lower than 20%.

## Discussion

In this paper, data simulation experiments were conducted on three sets of publicly available datasets, where the MM model was firstly missing using the complete dataset in order to obtain missing data $X^{MM}$ that corresponds to the real situation and to record the missing type of its missing values. Then the largest complete subset $X^{Complete}$ was obtained by panning the missing data and the particle swarm search algorithm was used to determine the concentration threshold and the proportion of low concentration deletions as a percentage of overall deletions of the closest missing data. The MM algorithm was simulated on the largest subset $X^{Complete}$ to obtain data labels with deletion types. Features such as the number of consecutively missing metabolites, the number of consecutively missing samples, the mean, the median, the minimum, the maximum of each metabolite, the rate of missing metabolites, and the high and low concentrations of the metabolites and their belonging to the metabolite were used to construct the XGBoost classifiers.

The results of experimental analysis show that the PX-MDC model proposed in this paper has high accuracy and fast searching speed in concentration threshold searching, and the PX-MDC model has better features and higher accuracy compared to the model proposed by Dekermanjian et al. In addition, Dekermanjian et al. method uses an assumption that the missing values of the same metabolite belong to the same missing type. On the contrary, the PX-MDC model proposed in this paper performs more accurately in the prediction of deletion types for the same metabolite and is able to distinguish between different deletion types, which is more in line with the reality of metabolomics data.

When performing MM model missingness, this paper assumes that higher than the overall metabolite concentration average is set as high concentration, lower than 1/10 of the overall metabolite concentration average is set as low concentration, and the rest is medium concentration. At the same time, the missing rate of low concentration is set to account for the overall missing rate in the range of 40–60% with a step size of 5%. Therefore, for each data, the concentration threshold is set differently, which can avoid the result error caused by the concentration threshold. In the modeling process, for any one copy of missing data, it is necessary to search for the concentration threshold, and the particle swarm search algorithm can be used to quickly and reliably find a solution within the acceptable error range, which shows that the algorithm has a certain degree of universality.

In summary, this paper proposes the PX-MDC modeling method in constructing a classifier model for missing types of metabolomics data, and verifies the validity and superiority of the method in experiments, which provides a reliable support for the subsequent data imputation using different strategies, and can better restore the distribution of the real and complete data, which can provide effective help for the discovery of potential biomarkers, the identification of key enzymes in metabolic pathways, and exploring the pathogenesis of diseases.

## Supplementary Information

Supplementary Information.

## Data Availability

All datasets are accessible through the Metabolomics Workbench (https://www.metabolomicsworkbench.org/).
