# Correction: Discrimination of missing data types in metabolomics data based on particle swarm optimization algorithm and XGBoost model

**DOI:** 10.1038/s41598-026-41012-5

**Published:** 2026-02-26

**Authors:** Yang Yuan, Jianqiang Du, Jigen Luo, Yanchen Zhu, Qiang Huang, Mengting Zhang

**Affiliations:** 1 School of Computer Science, Jiangxi University of Chinese Medicine, Nanchang, 330004 China; 2 Key Laboratory of Artificial Intelligence in Chinese Medicine, Jiangxi University of Chinese Medicine, Nanchang, 330004 China

Correction to: *Scientific Reports* 10.1038/s41598-023-50646-8, publihed online 02 January 2024

The original version of this Article contained errors in Figure 6, where panel ST000419 was duplicated twice and panels ST000118 and ST000385 were omitted. The original Figure 6 appears below.

**Fig. 6:**
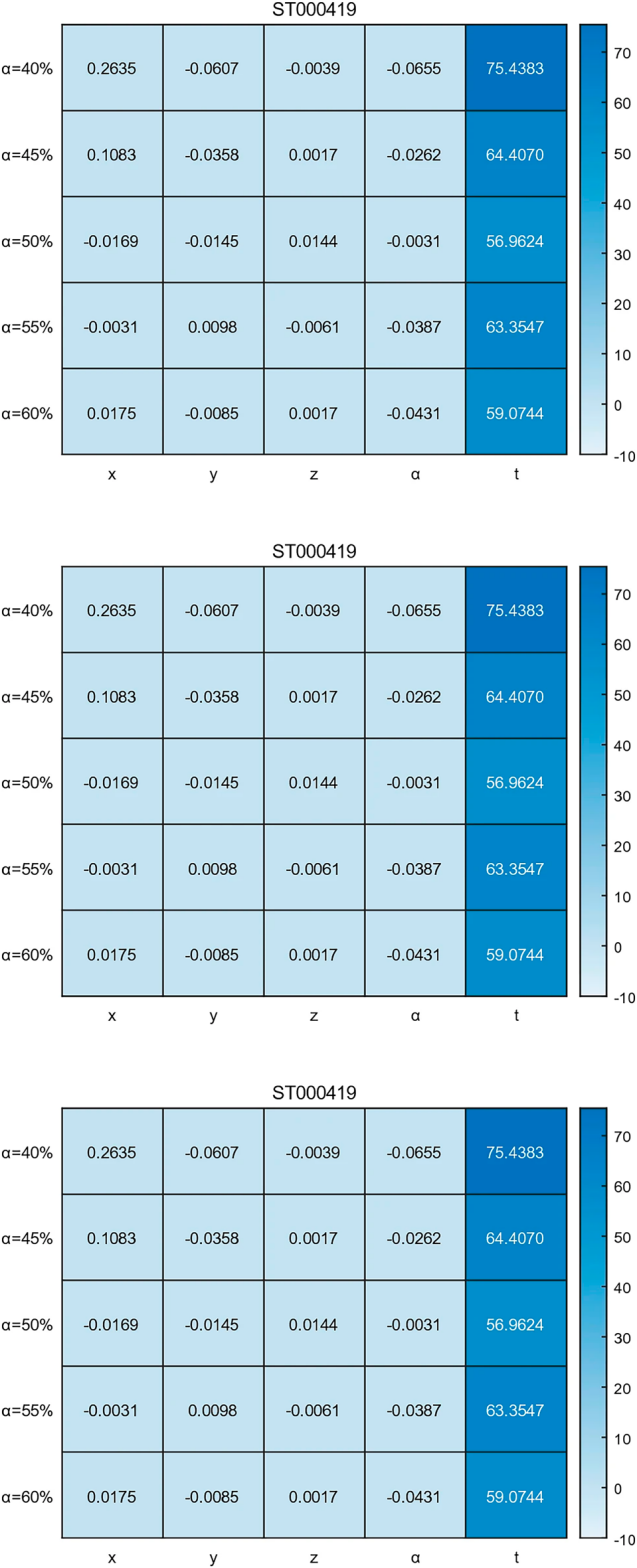
The search results of the particle swarm algorithm and the enumeration method were compared. The formula for calculating the percentage difference is the absolute value of the difference between the particle swarm results and the enumeration results divided by the particle swarm results. The heatmap clearly shows that the search time t has a greater impact than the difference.

The original Article has been corrected.

