# Supplementary material for: Discrimination of missing data types in metabolomics data based on particle swarm optimization algorithm and XGBoost model
Source: Sci Rep. 2024 Jan 2;14:152. doi: 10.1038/s41598-023-50646-8 (PMC10762217; doi:10.1038/s41598-023-50646-8)
Supplement: Supplementary file 1 — Supplementary Information. [file 41598_2023_50646_MOESM1_ESM.docx]

**SUPPLEMENT**

# Discrimination of Missing Data Types in Metabolomics Data Based on Particle Swarm Optimization Algorithm and XGBoost Model

Yang Yuan^1,^, Jianqiang Du *^1,2^, Jigen Luo^1,2^, Yanchen Zhu^1^, Qiang Huang^1^, Mengting Zhang^1^

^1.^School of Computer Science, Jiangxi University of Chinese Medicine, Nanchang 330004, China

^2^Key Laboratory of Artificial Intelligence in Chinese Medicine, Jiangxi University of Chinese Medicine, Nanchang 330004, China

#Corresponding author; email address: jianqiang_du@163.com

*Contributed equally

# Section S1: Figures

In Supplementary Figure 1-8, the prediction accuracies of three different sets of features on four different classifiers are compared. Where Supplementary Figure 1, Supplementary Figure 3, Supplementary Figure 5 and Supplementary Figure 7 shows the accuracy on the validation set and Supplementary Figure 2, Supplementary Figure 4, Supplementary Figure 6 and Supplementary Figure 8 shows the accuracy on X^MM^.

Number of consecutive missing metabolites was characterized by the number of consecutive missing metabolites, the mean, median, minimum, and maximum values for each metabolite, the missing rate of the metabolite, the name of the metabolite, and the concentration of the metabolite to which it belongs.

Number of consecutive missing samples was characterized by the number of consecutive missing samples, the mean, median, minimum, and maximum values for each metabolite, the missing rate of the metabolite, the name of the metabolite, and the concentration of the metabolite to which it belongs.

PX-MDC was characterized by the number of consecutively missing metabolites, the number of consecutively missing samples, the mean, the median, the minimum, the maximum of each metabolite, the rate of missing metabolites, the name of the metabolite, and the level of the metabolite's concentration to which it belongs.

| 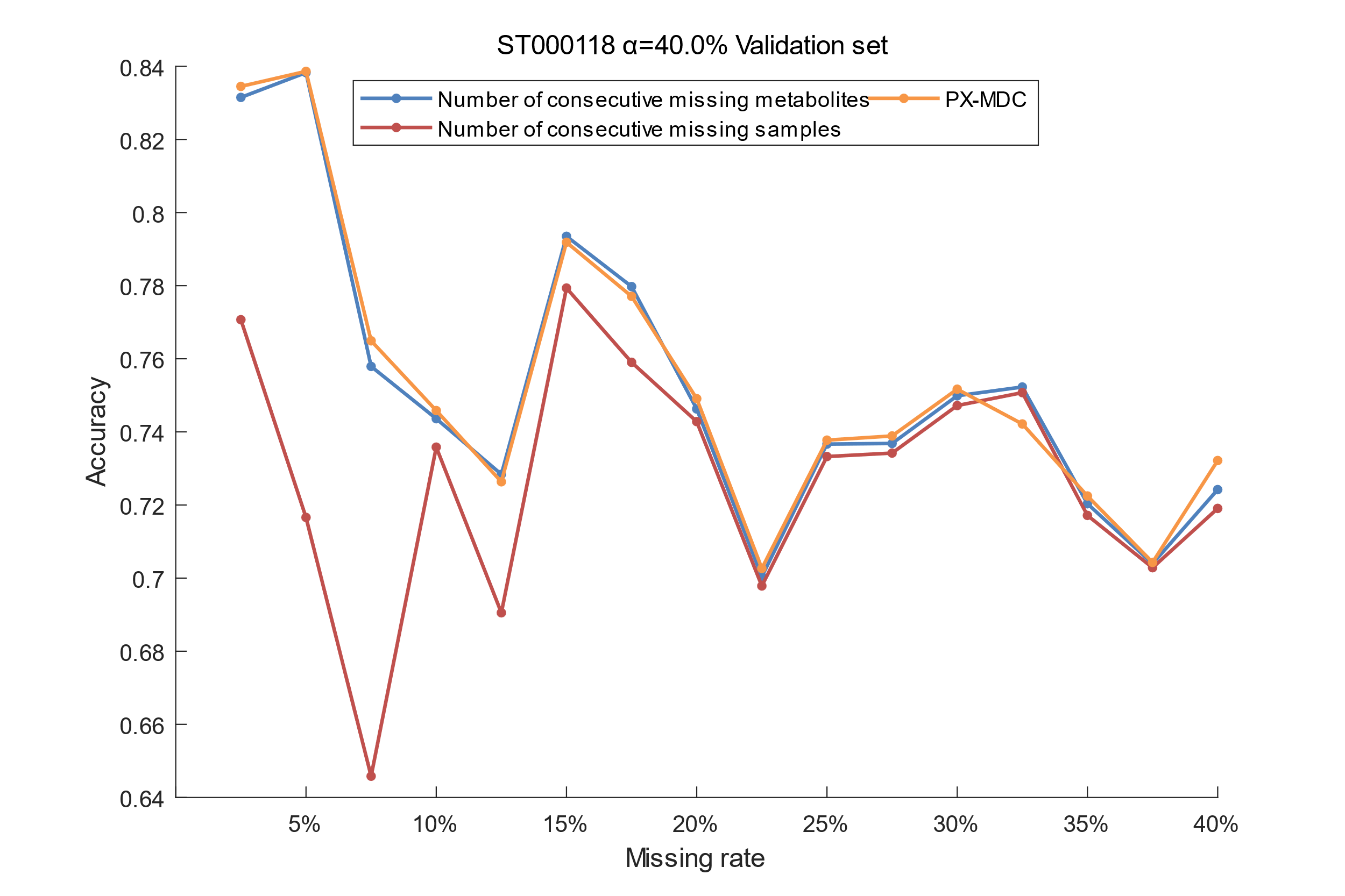 | 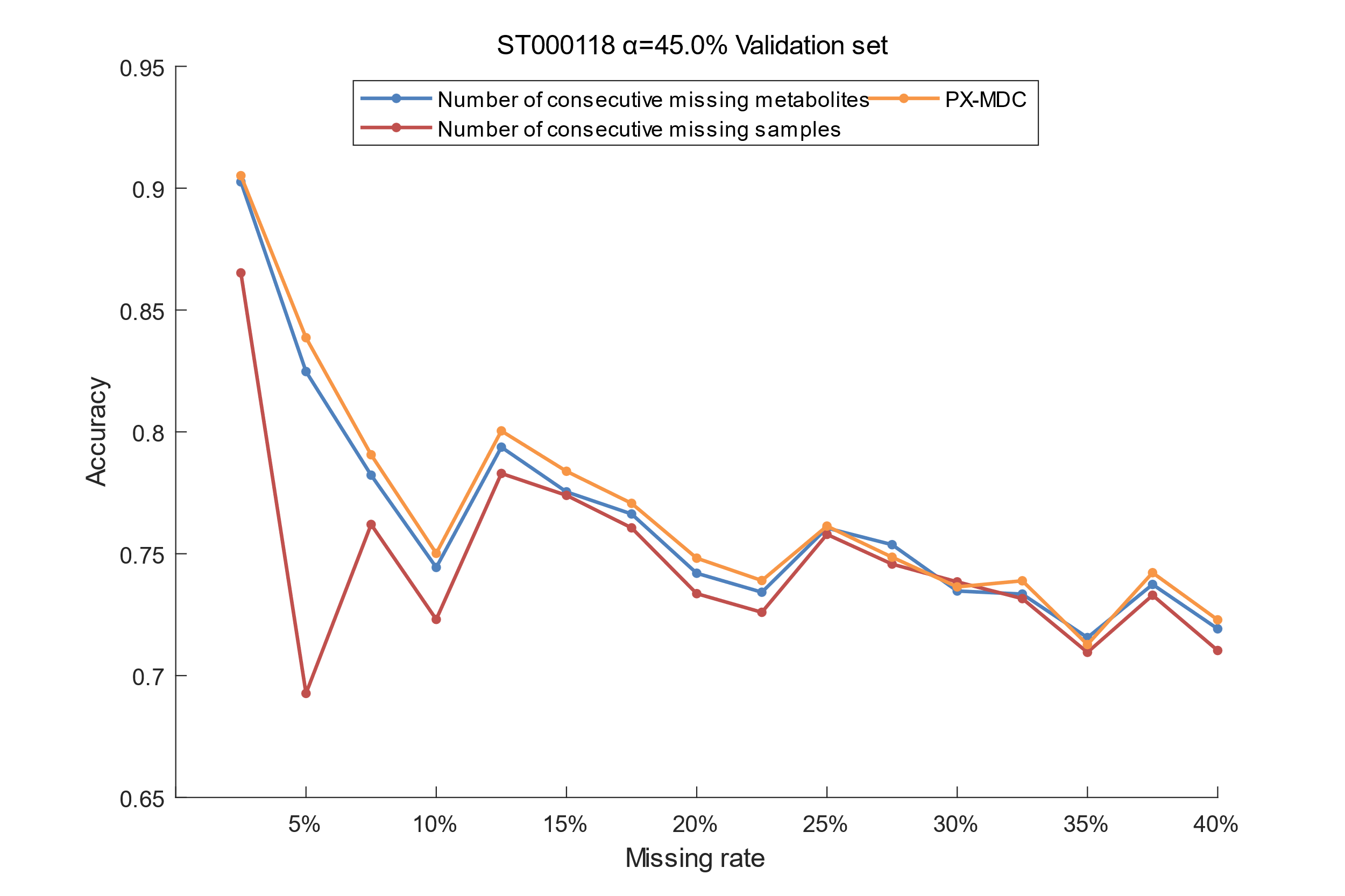 | 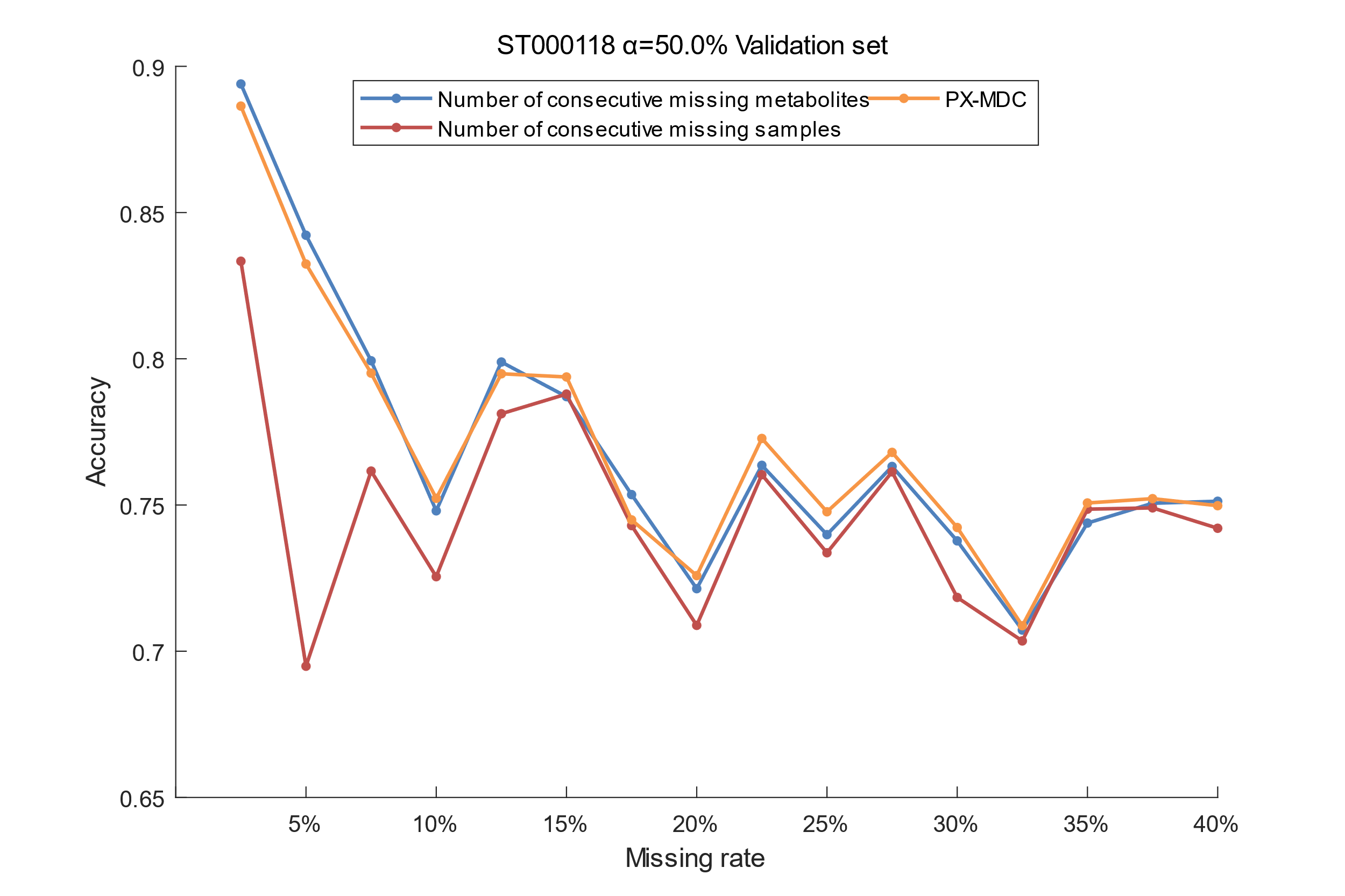 |
| --- | --- | --- |
| 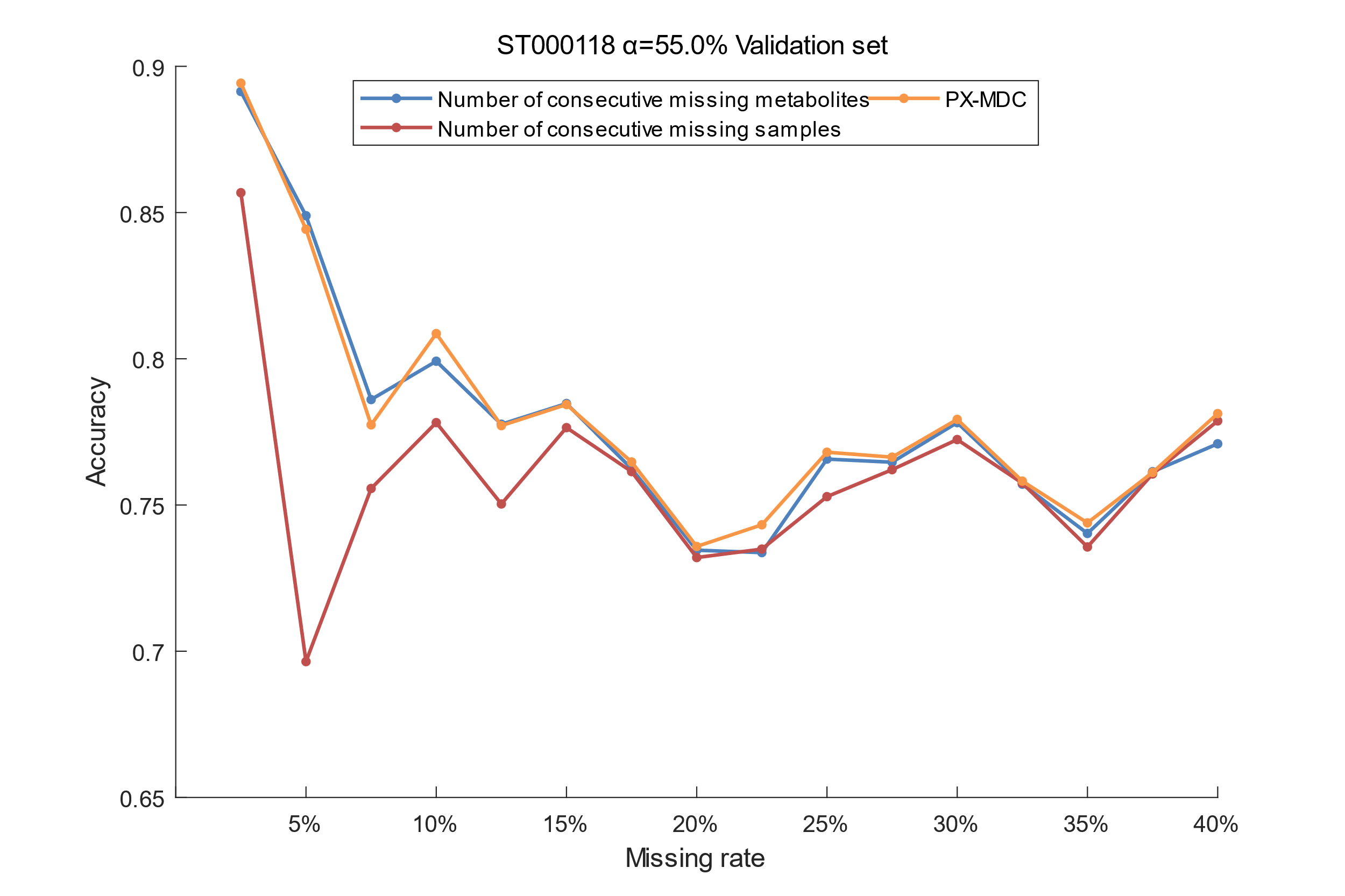 | 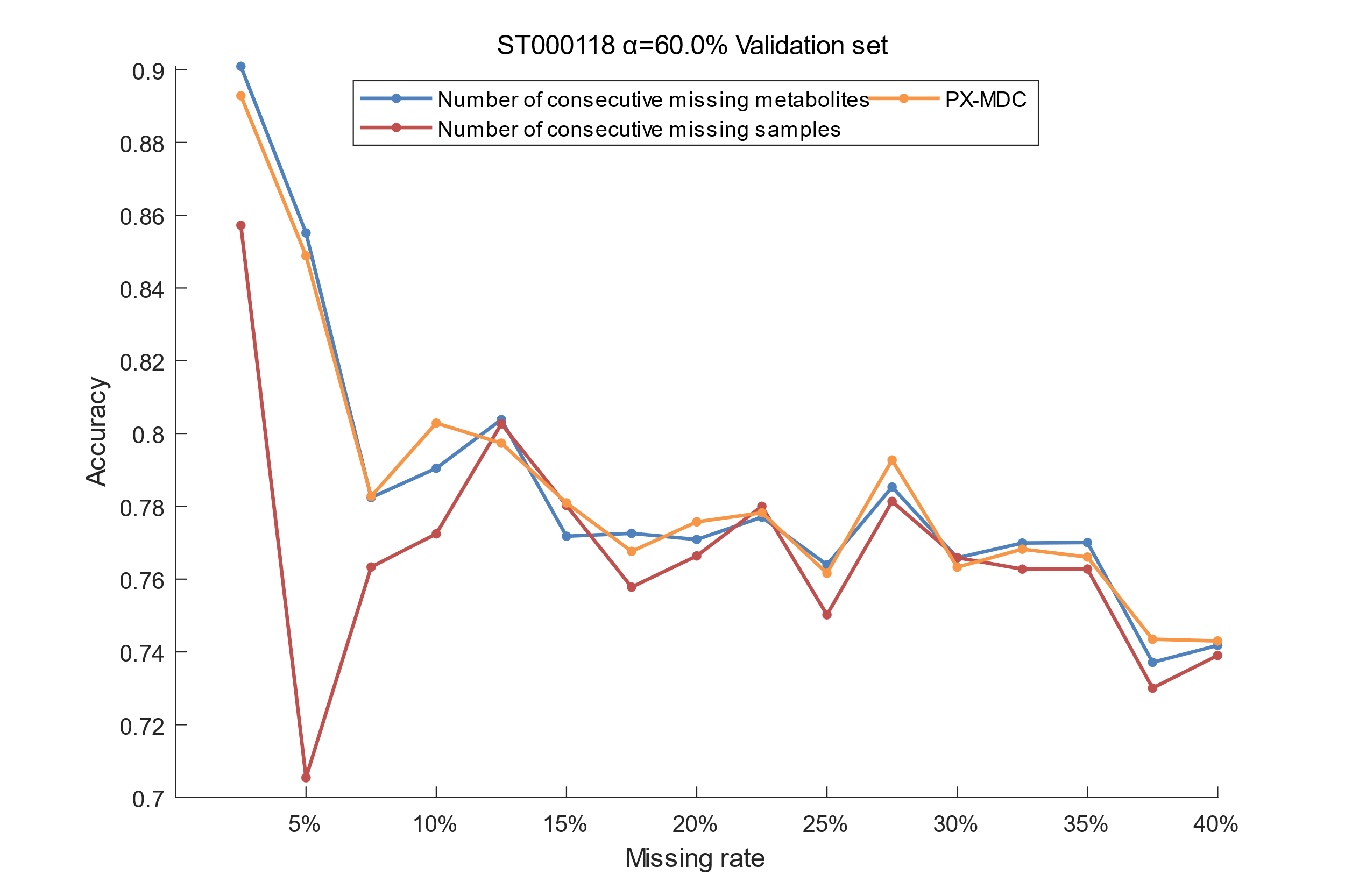 | 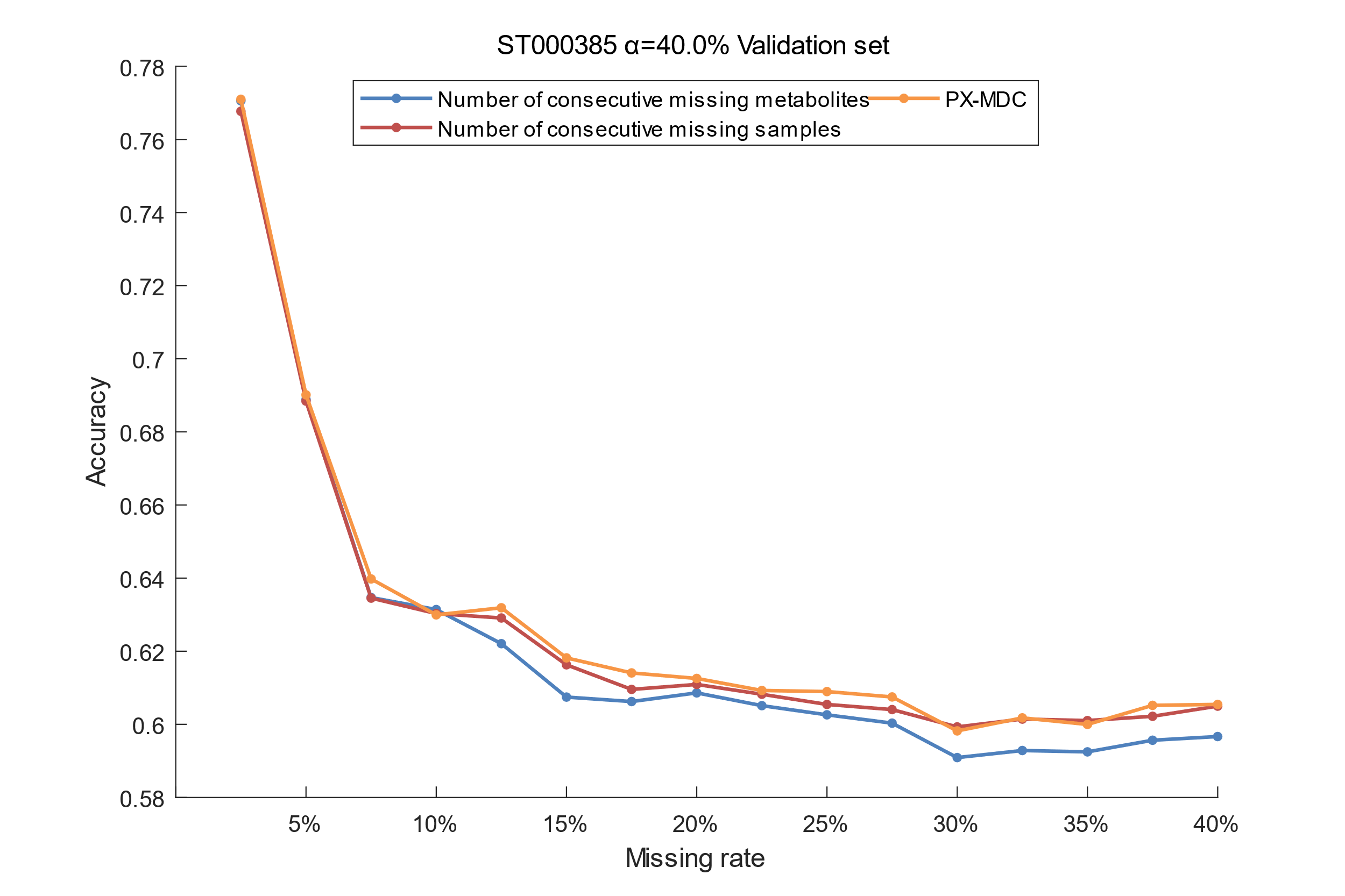 |
| 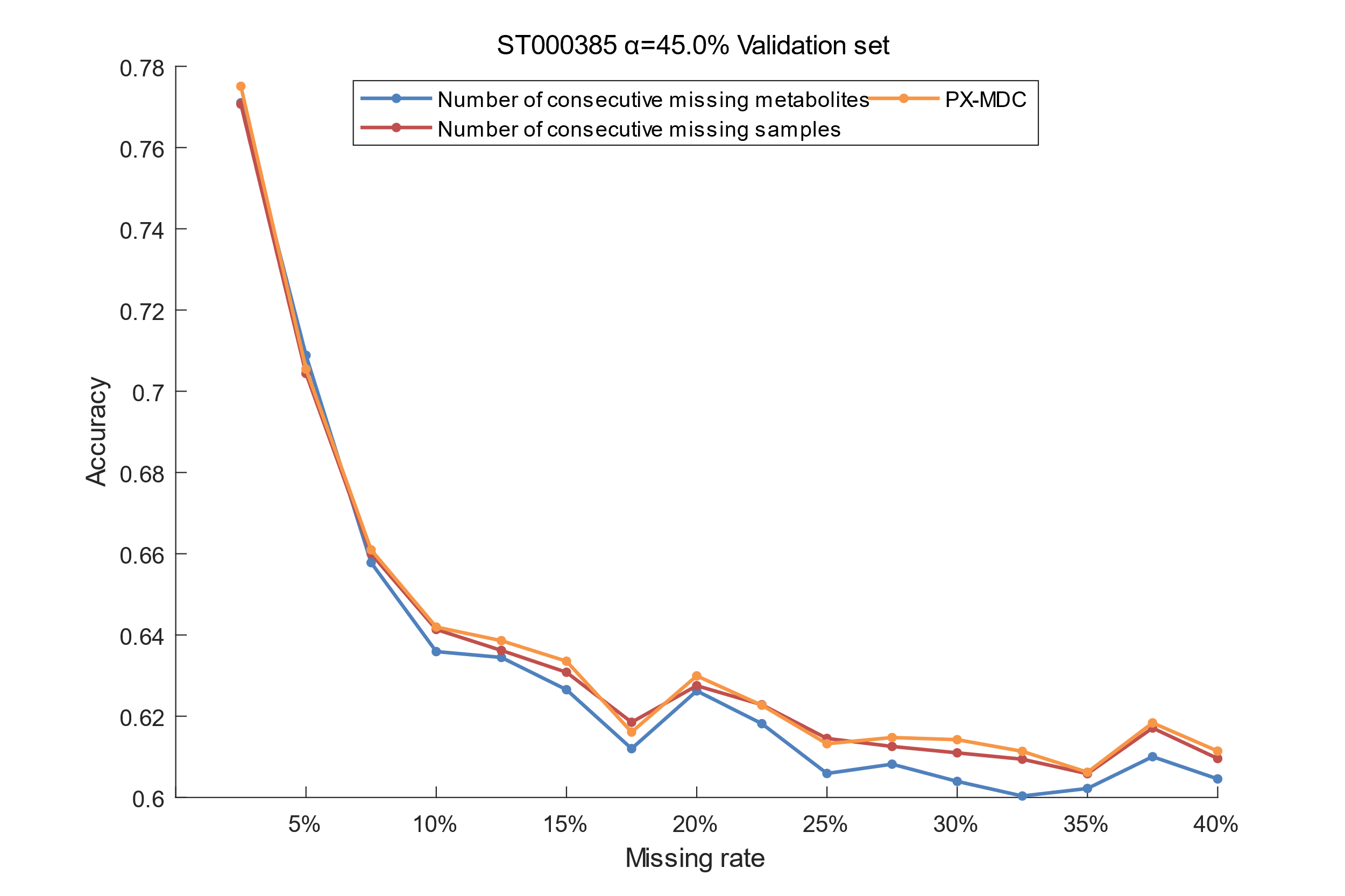 | 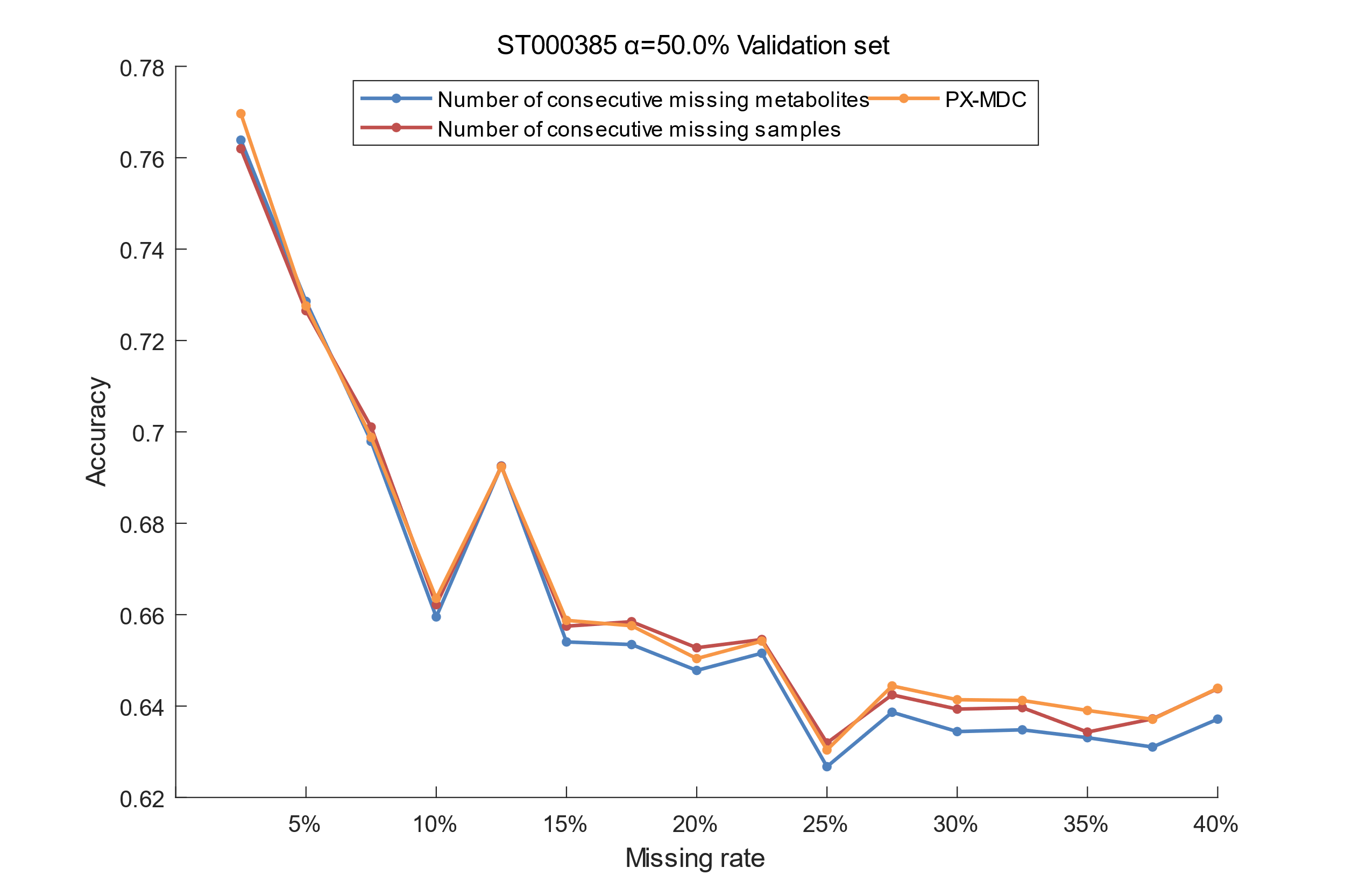 | 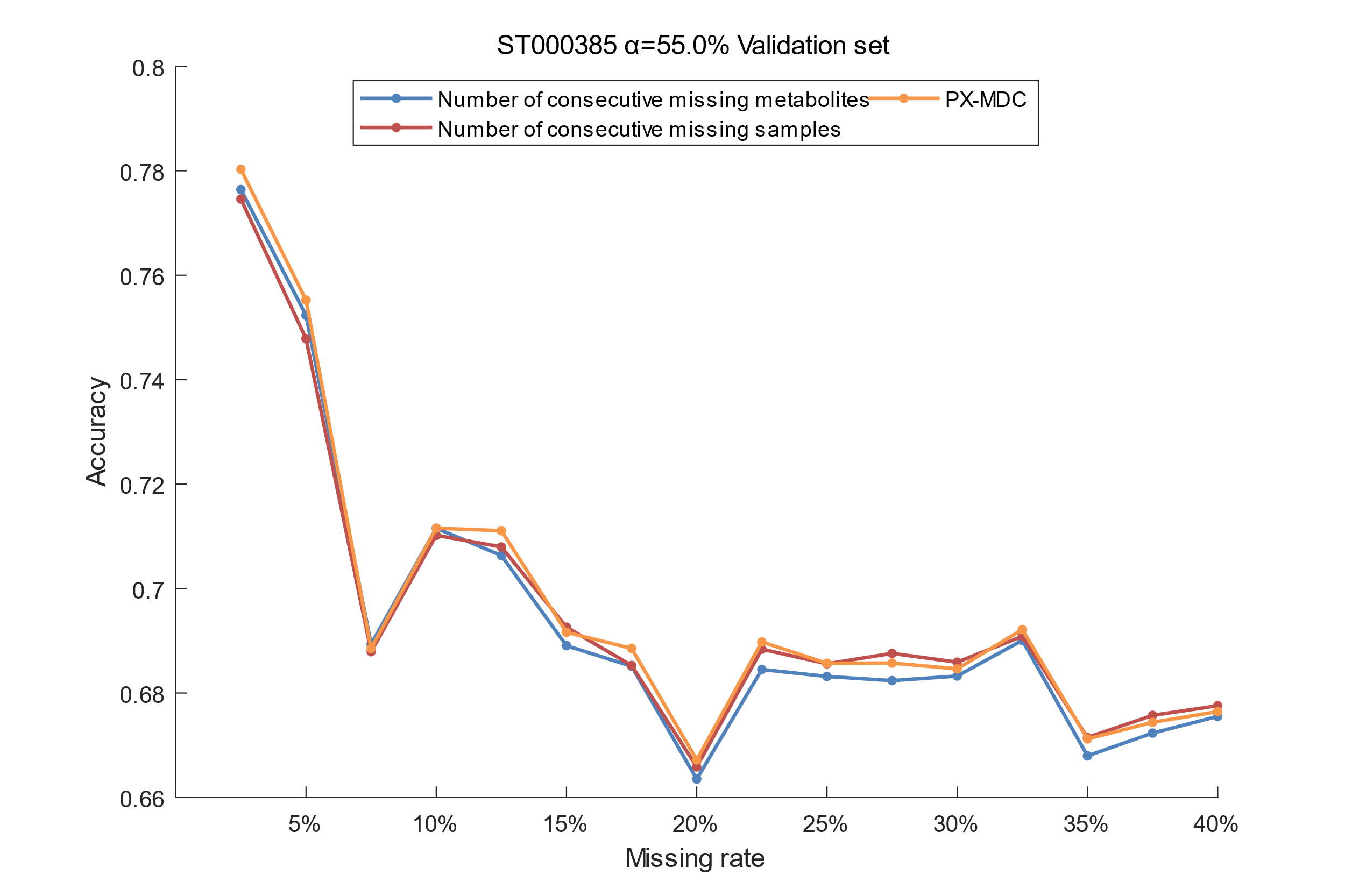 |
| 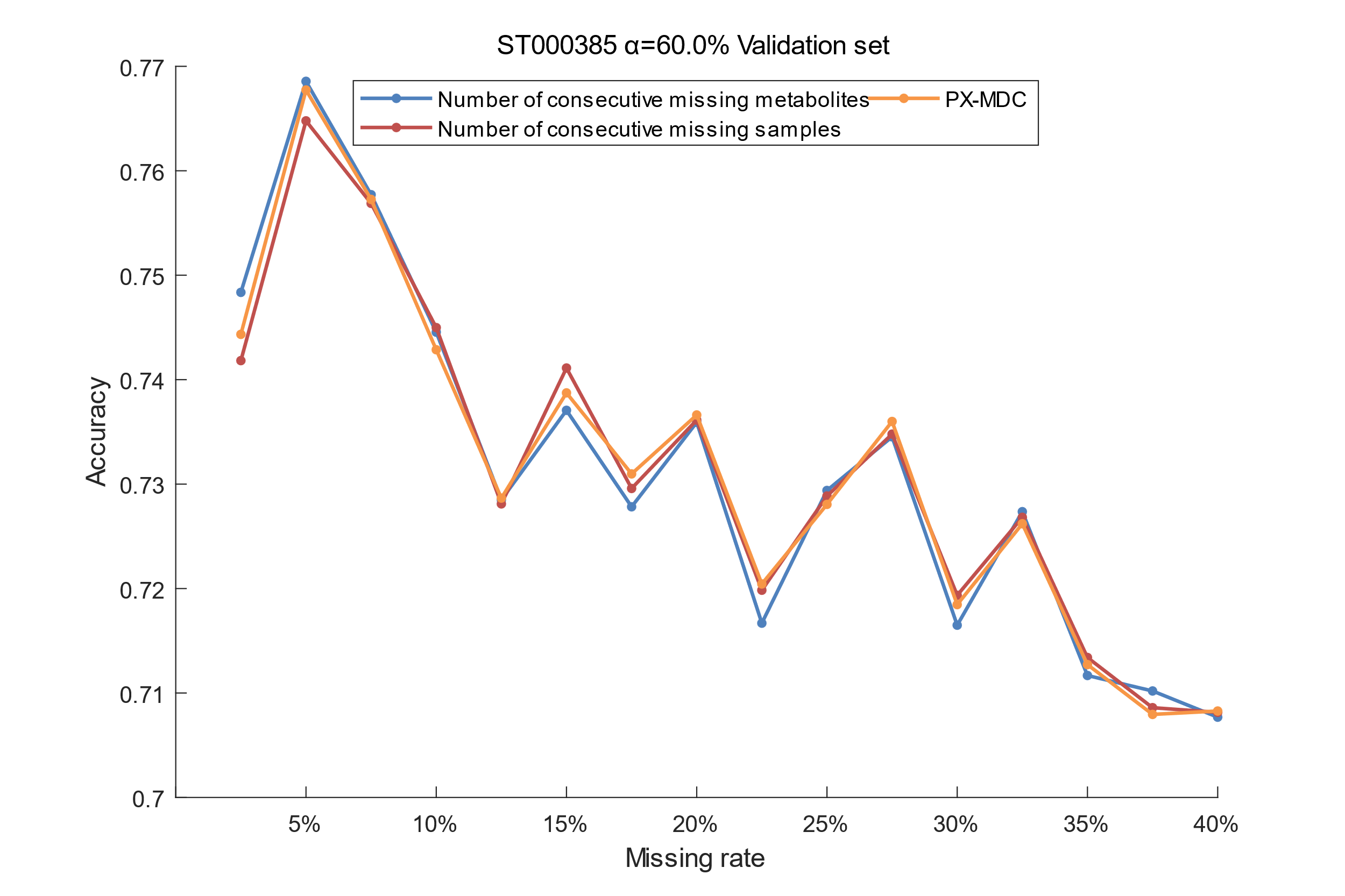 | 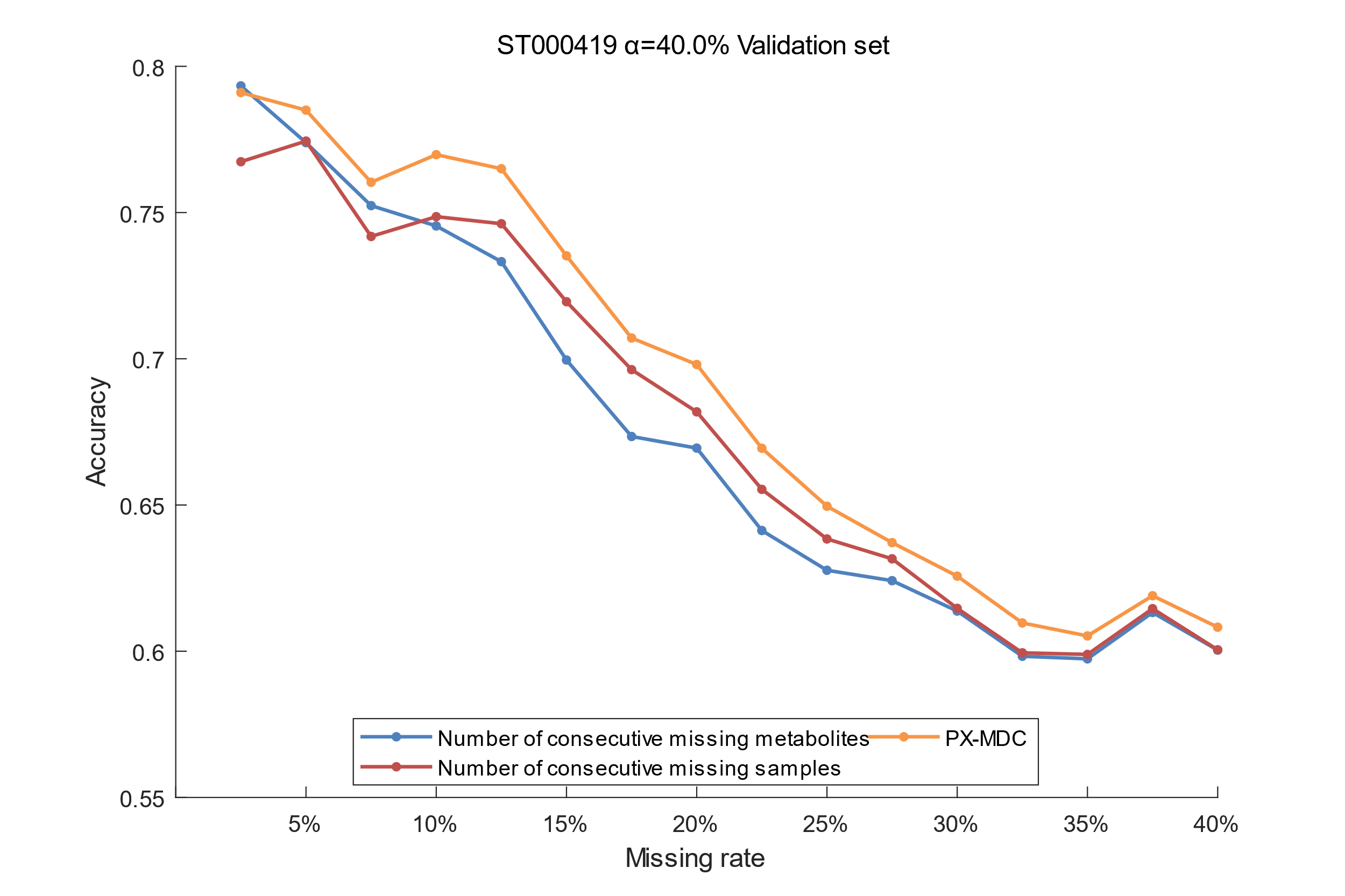 | 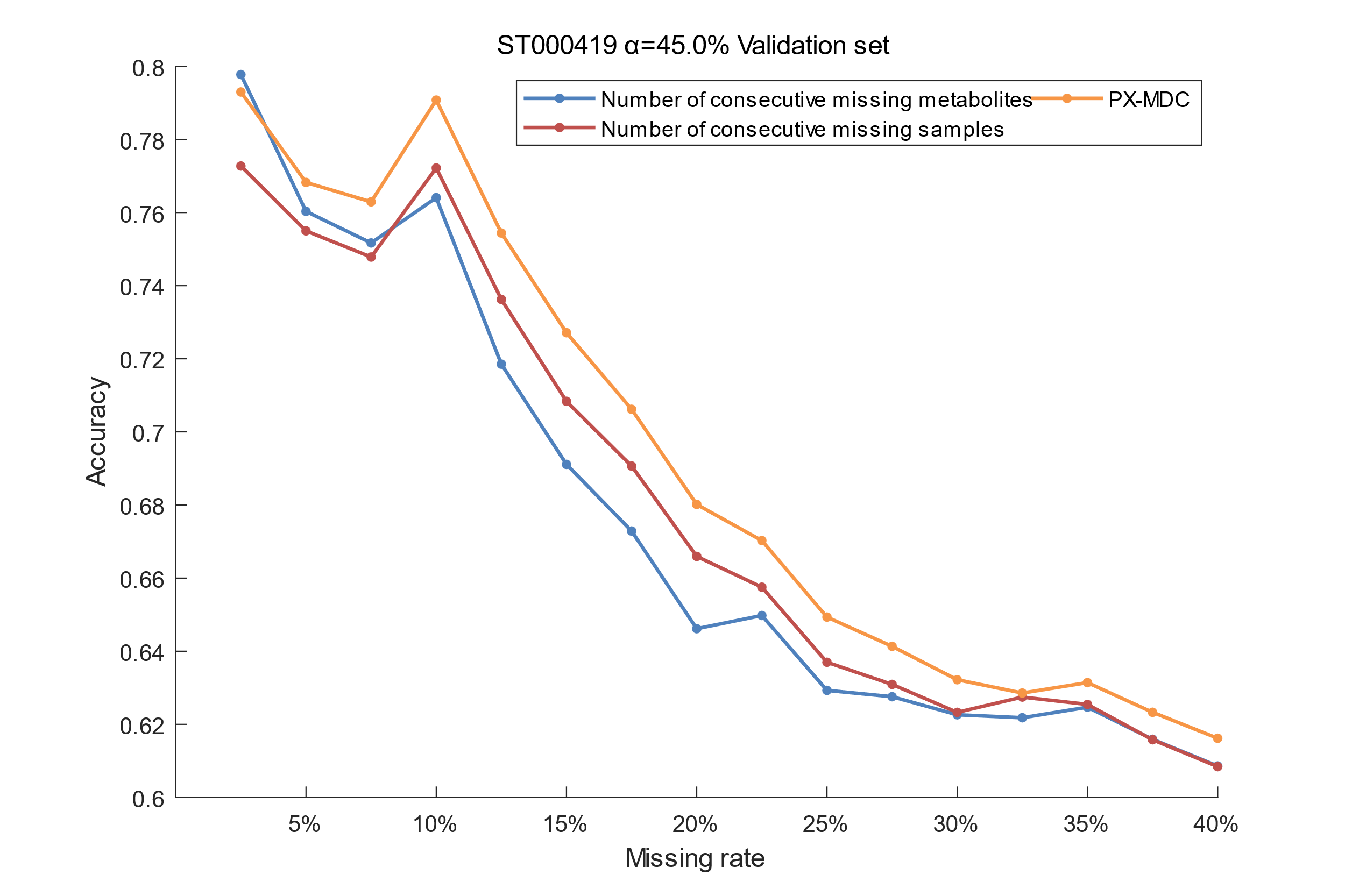 |
| 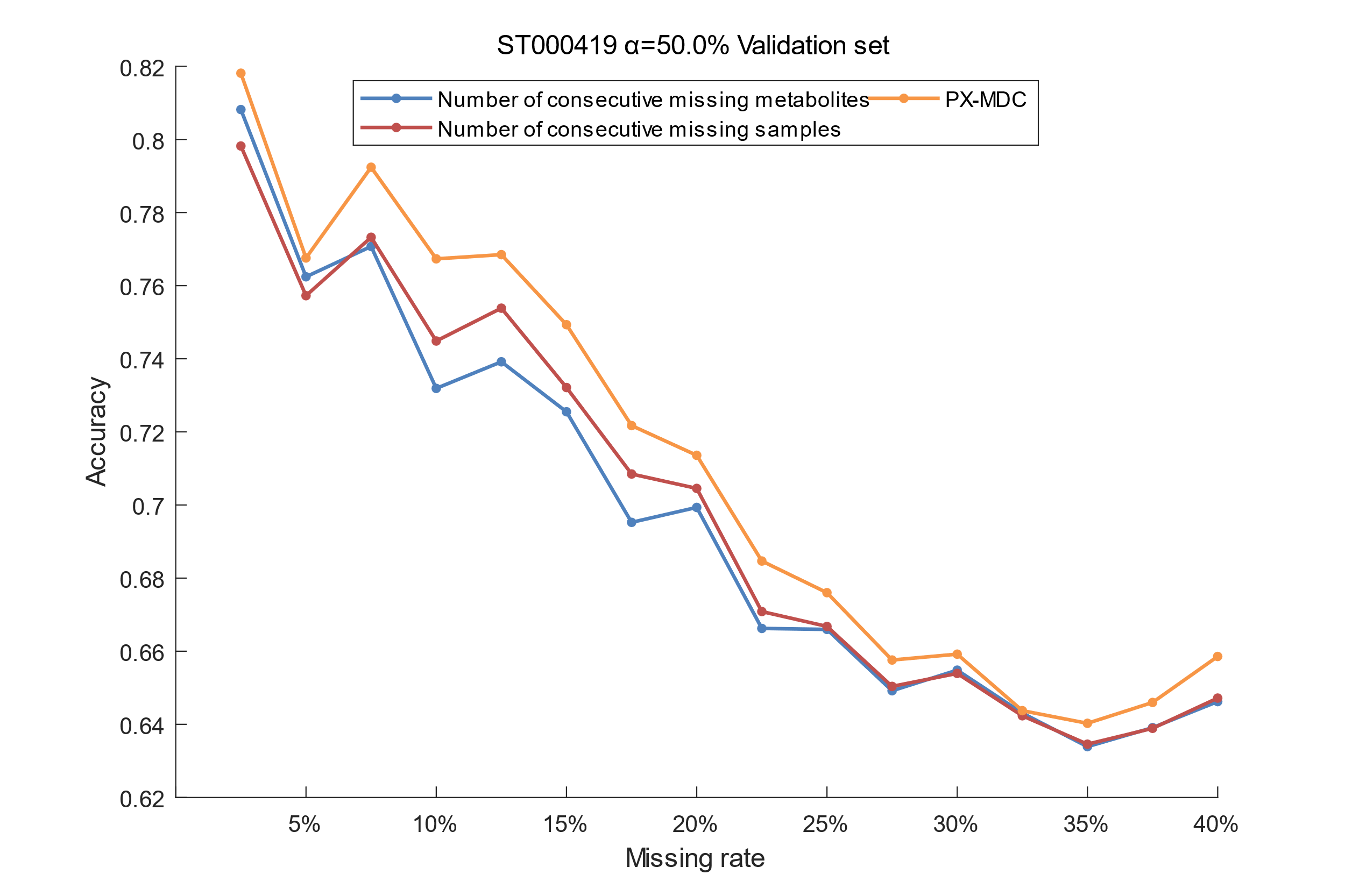 | 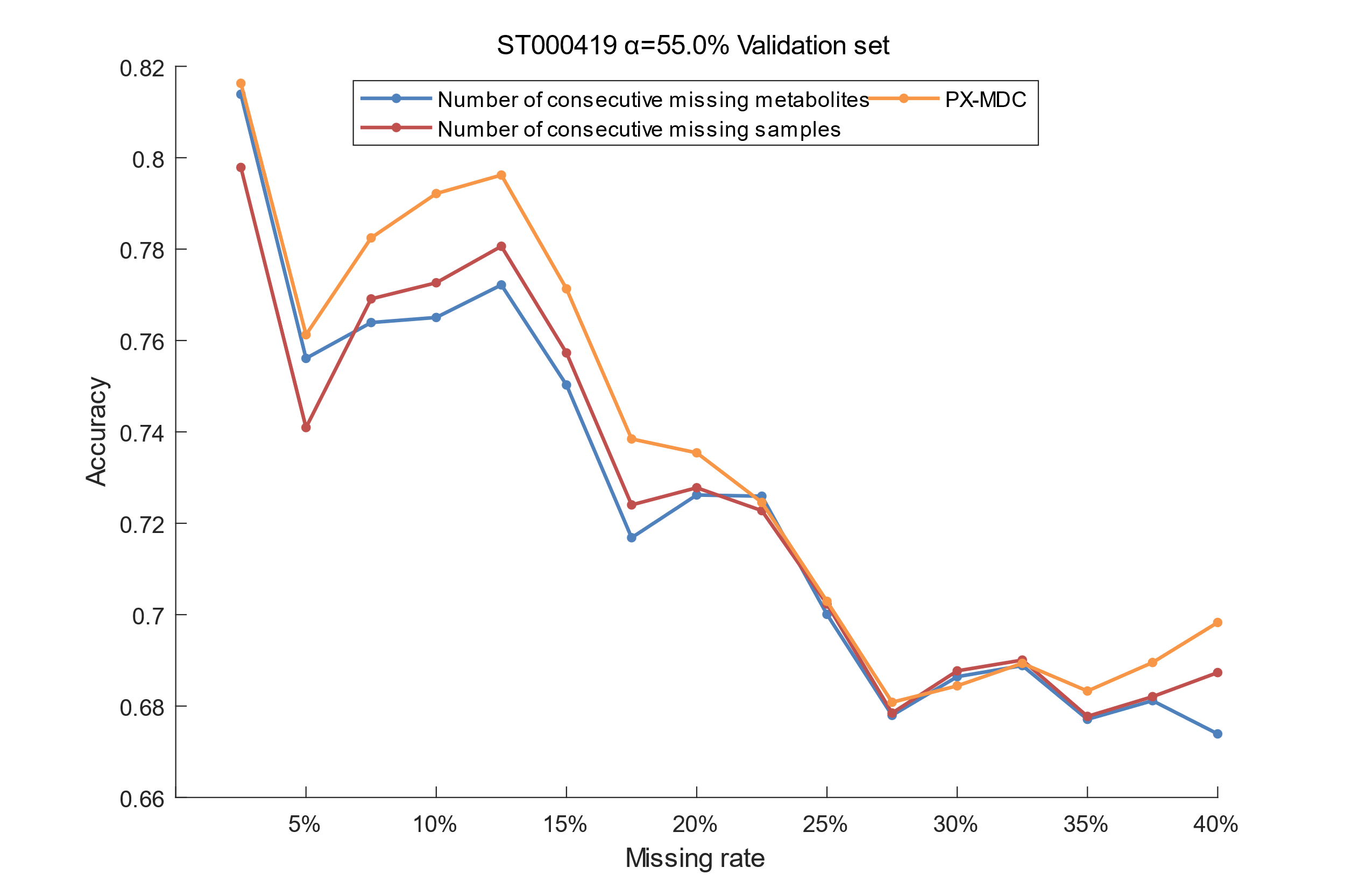 | 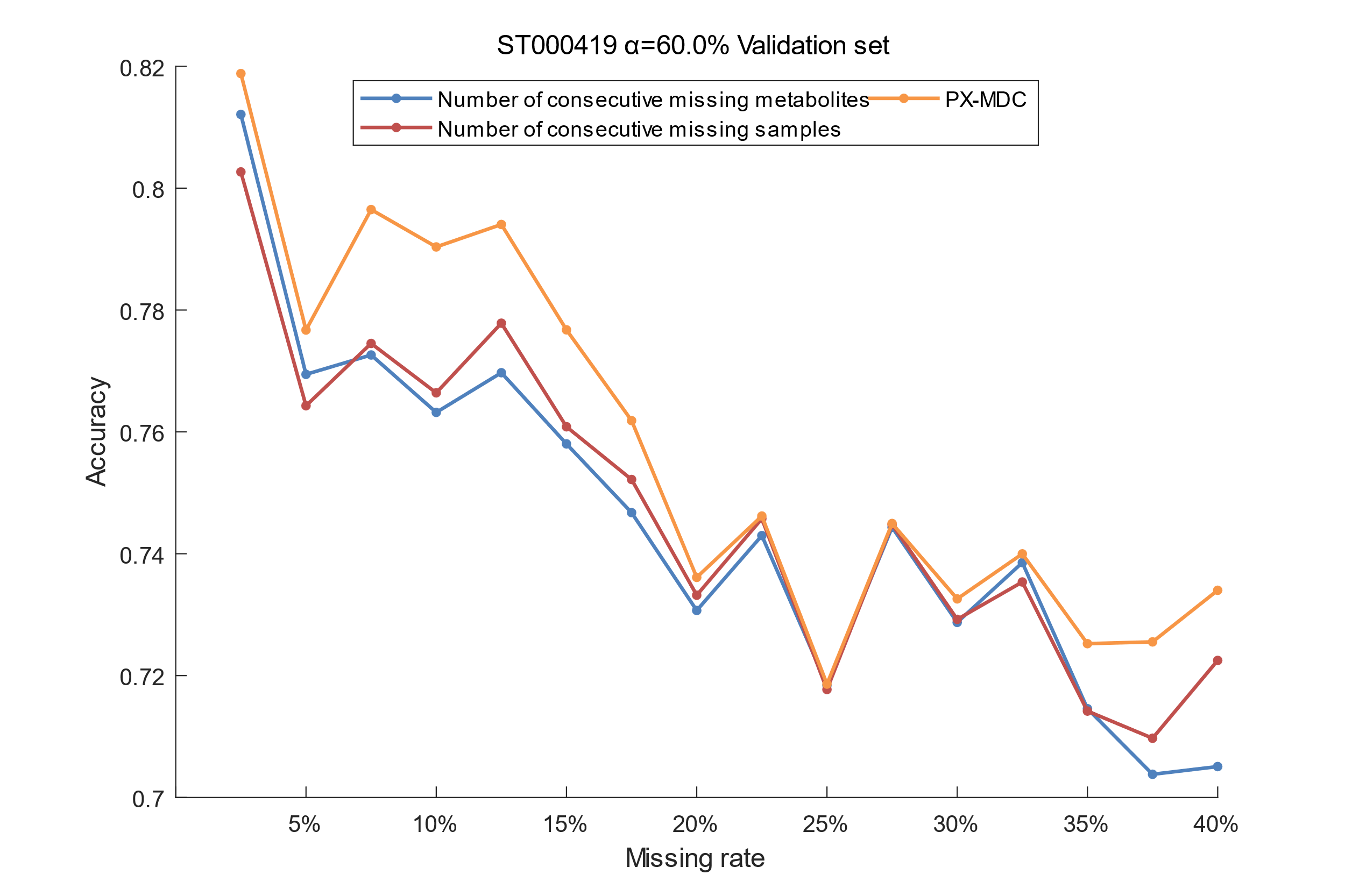 |

**Supplementary Figure 1.** Accuracy of validation sets in XGBoost models for different feature.

| 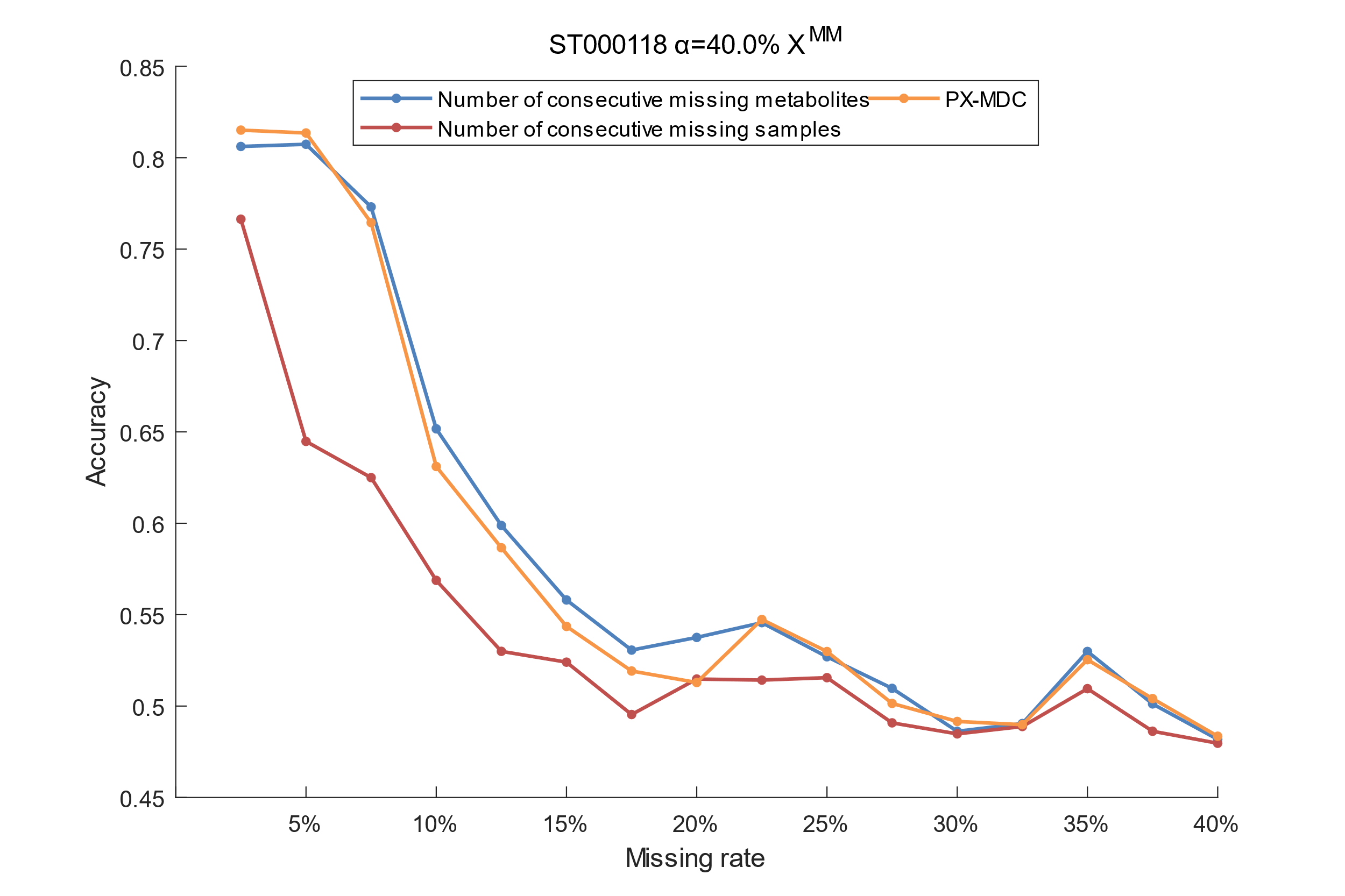 | 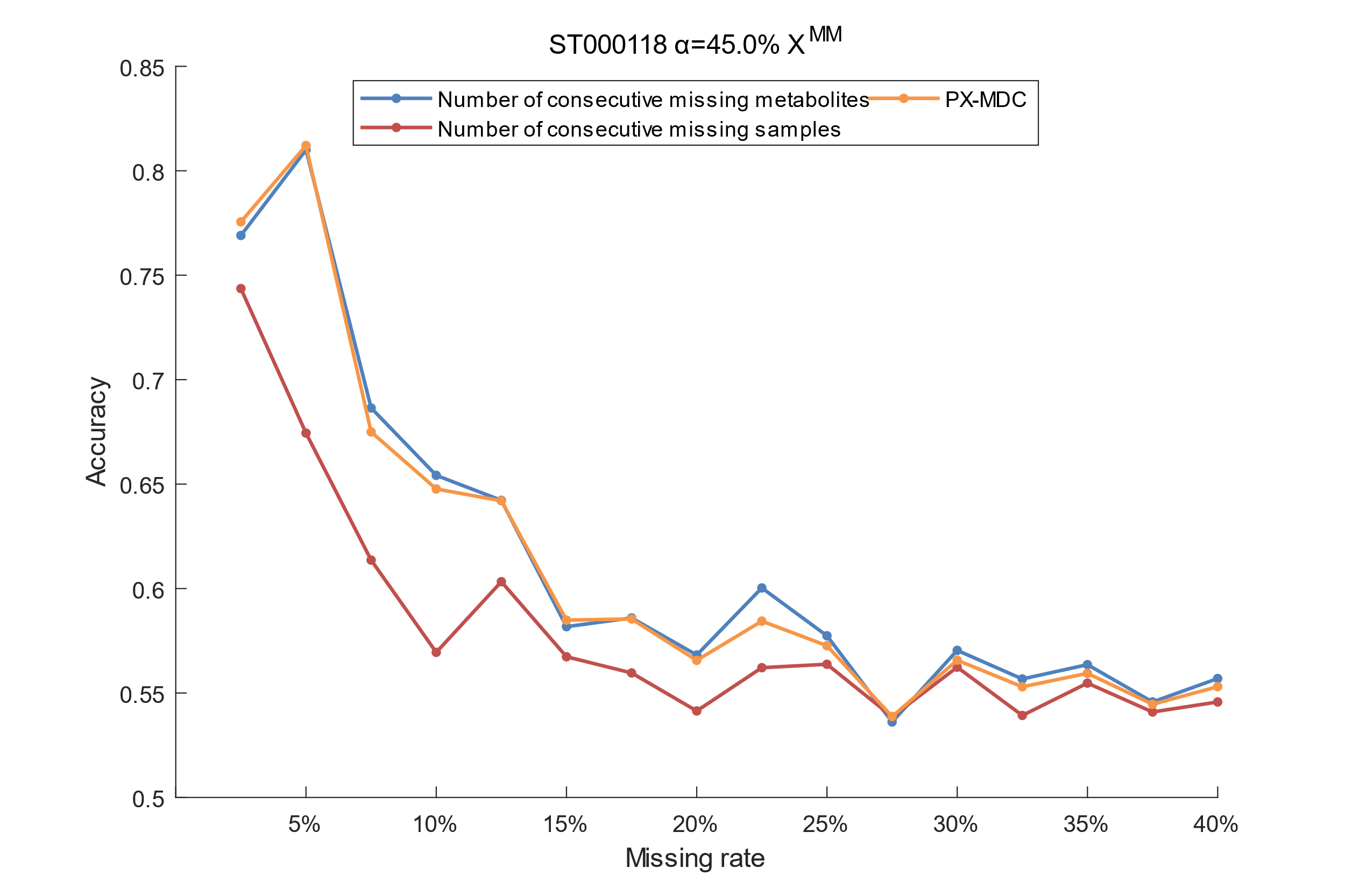 | 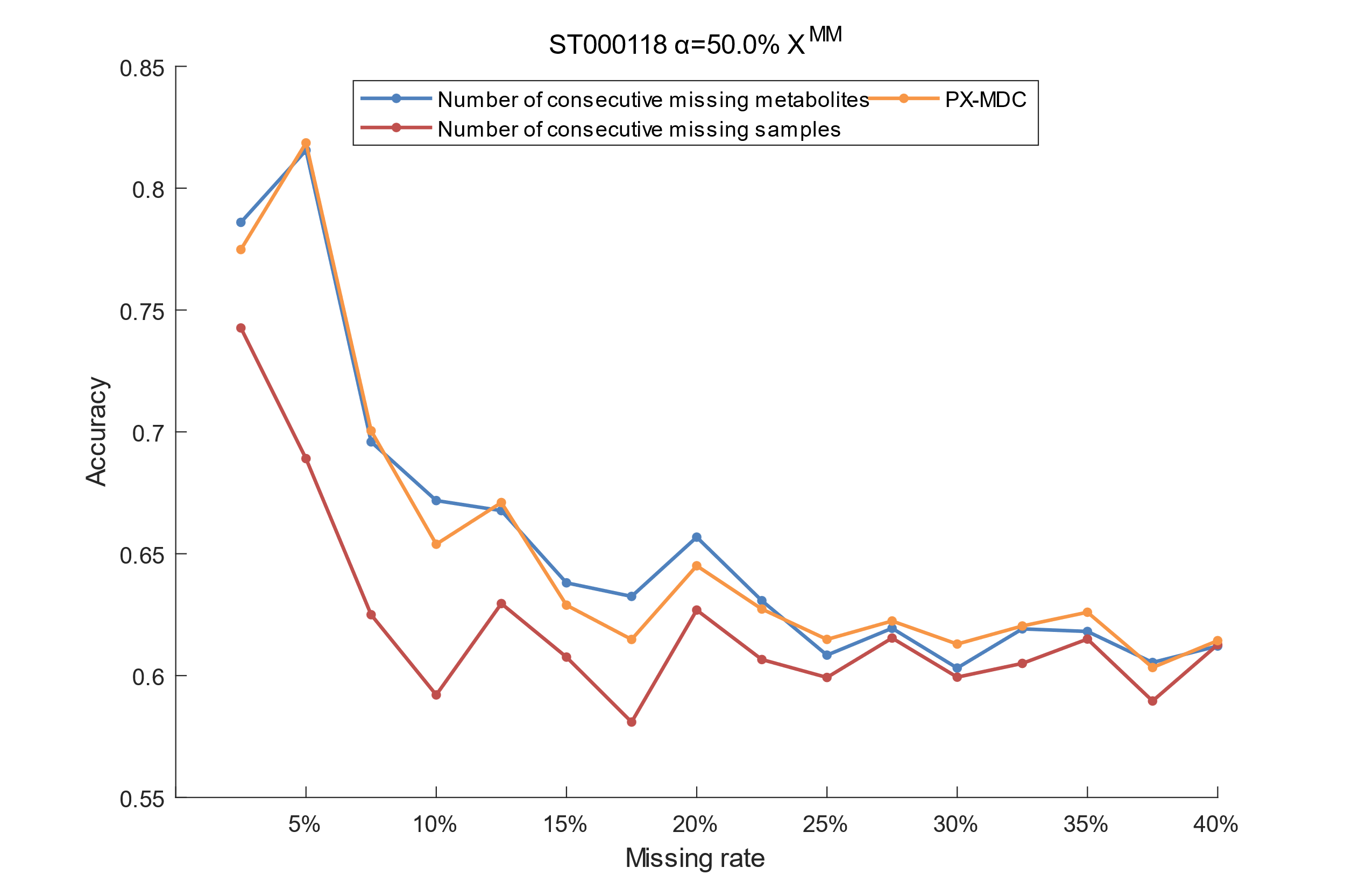 |
| --- | --- | --- |
| 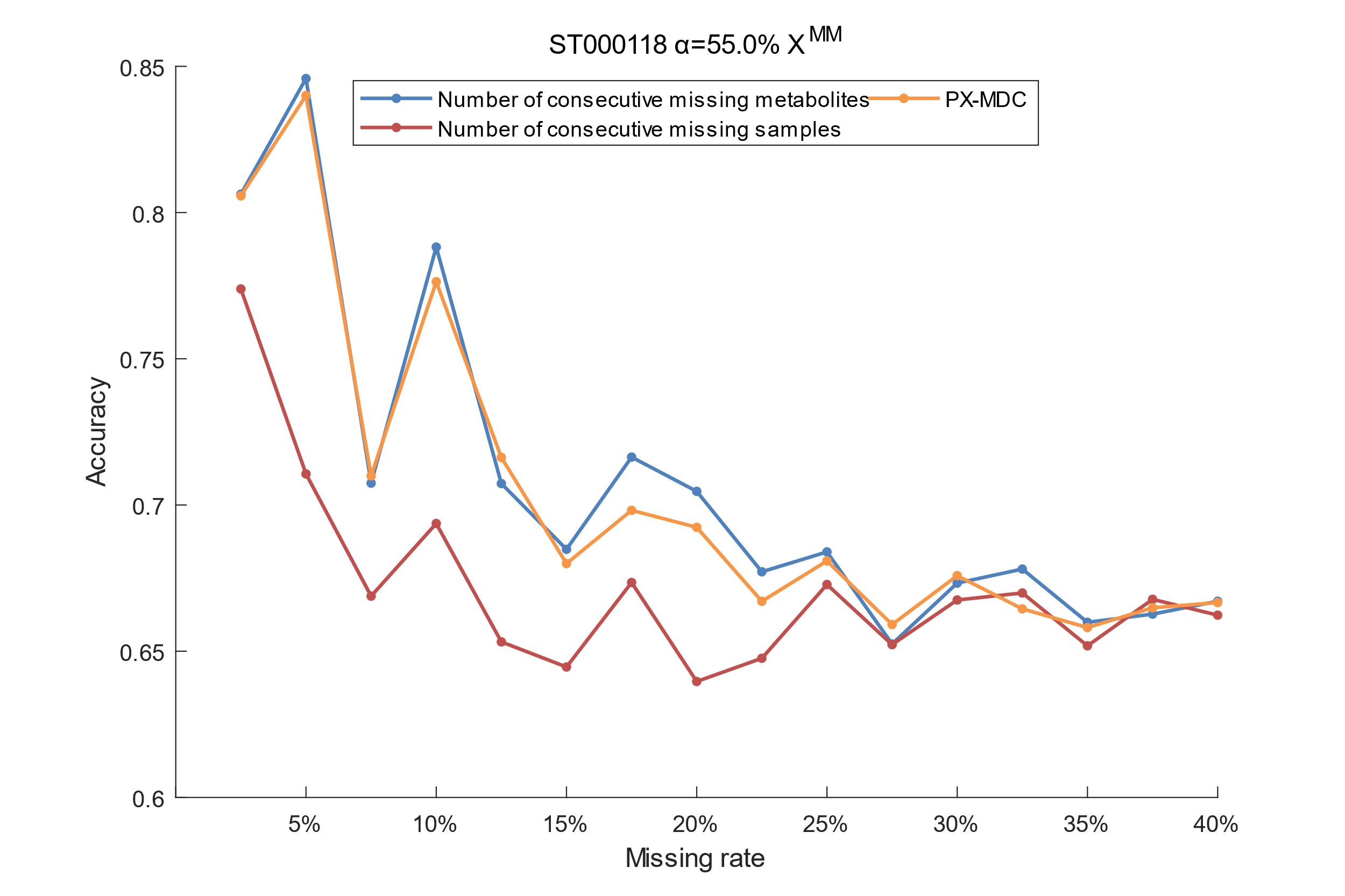 | 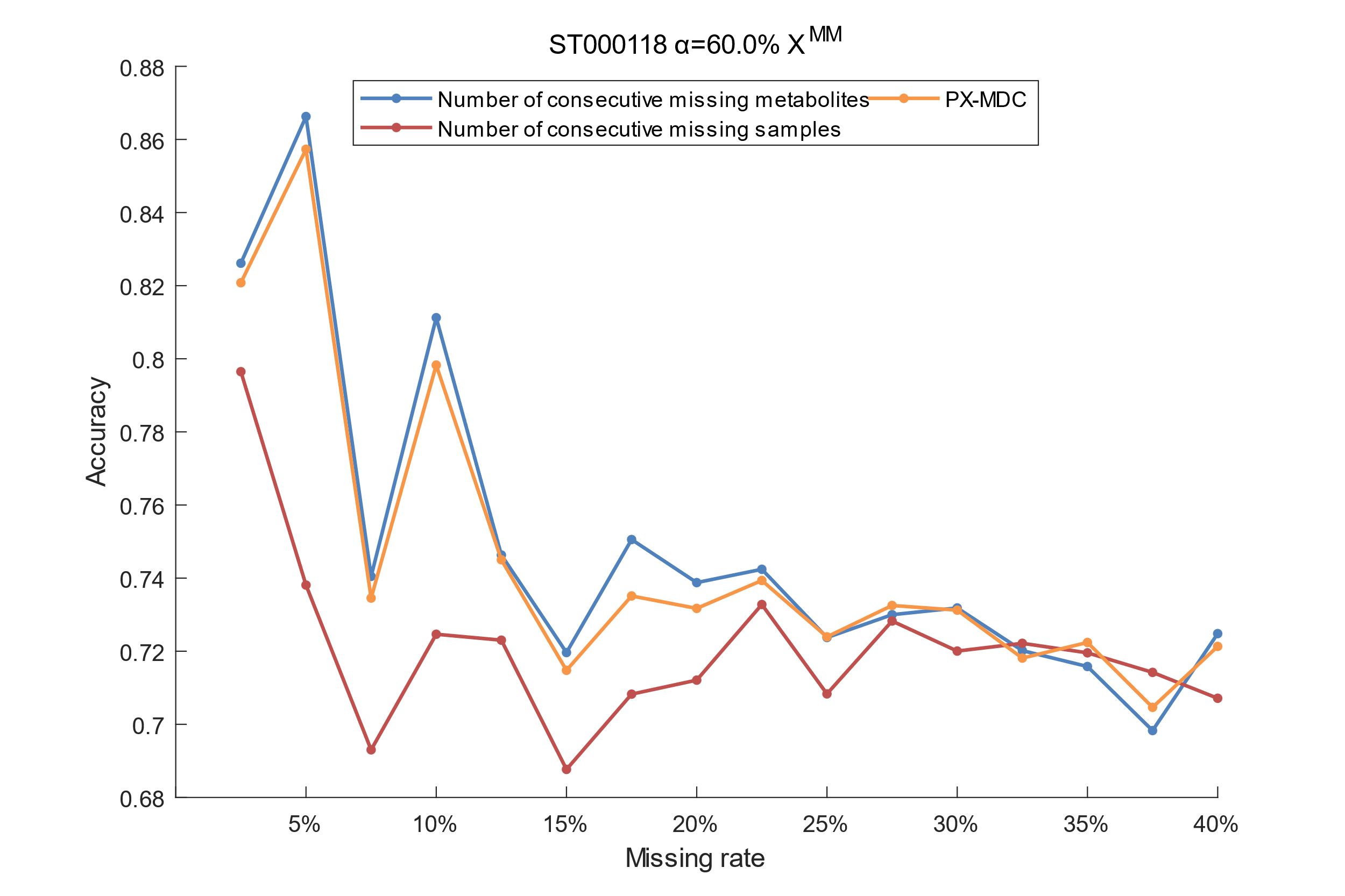 | 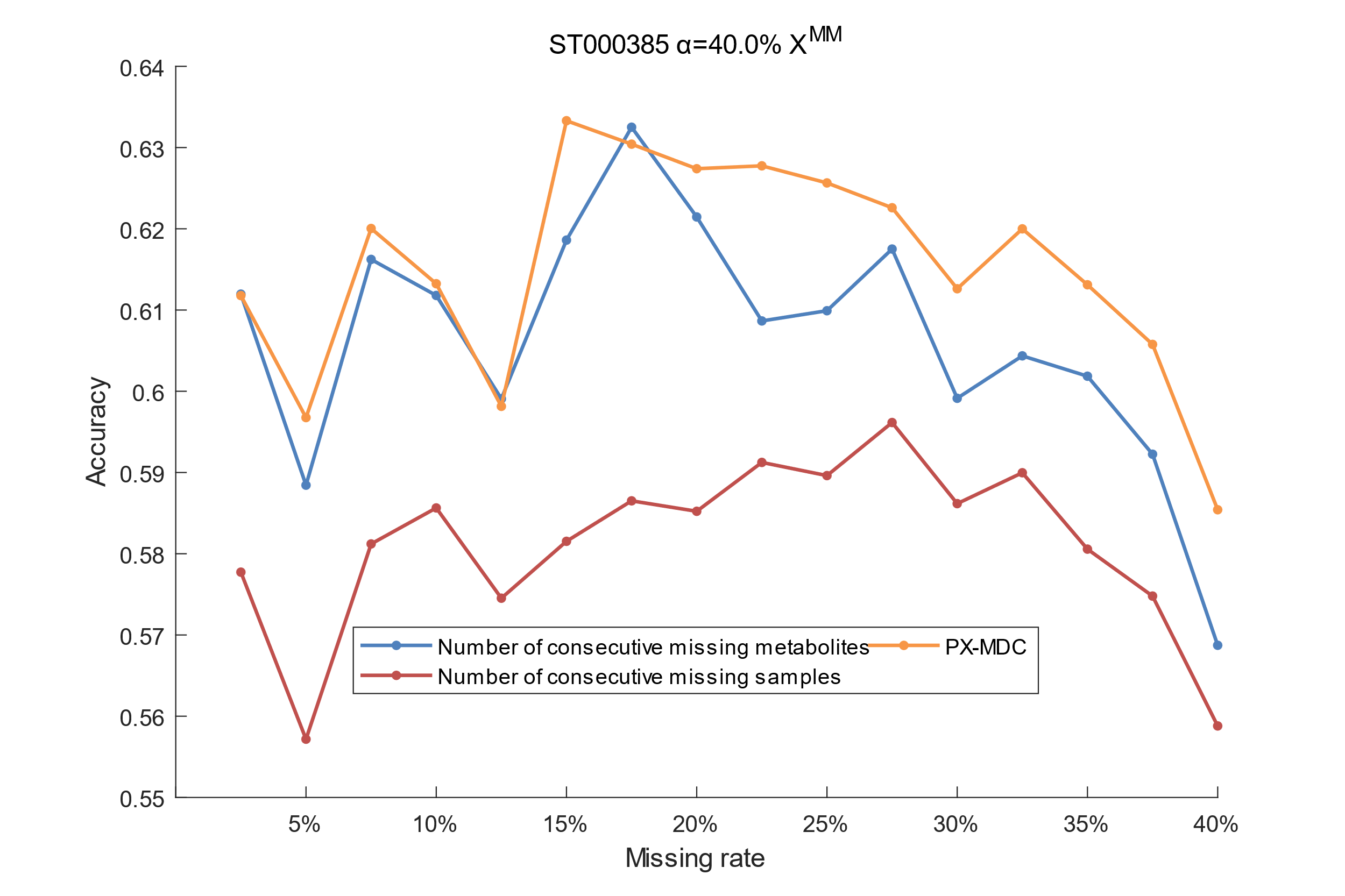 |
| 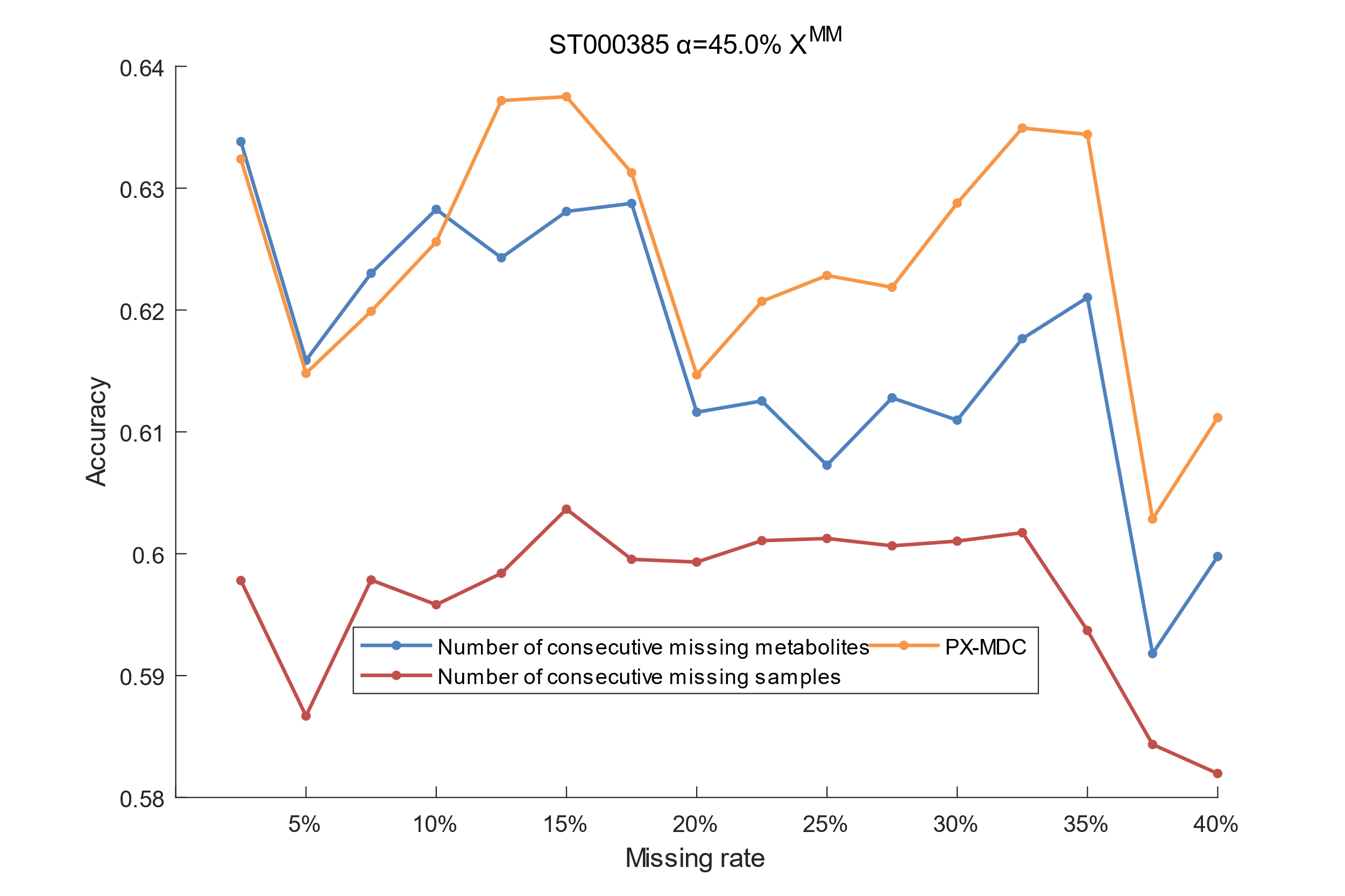 | 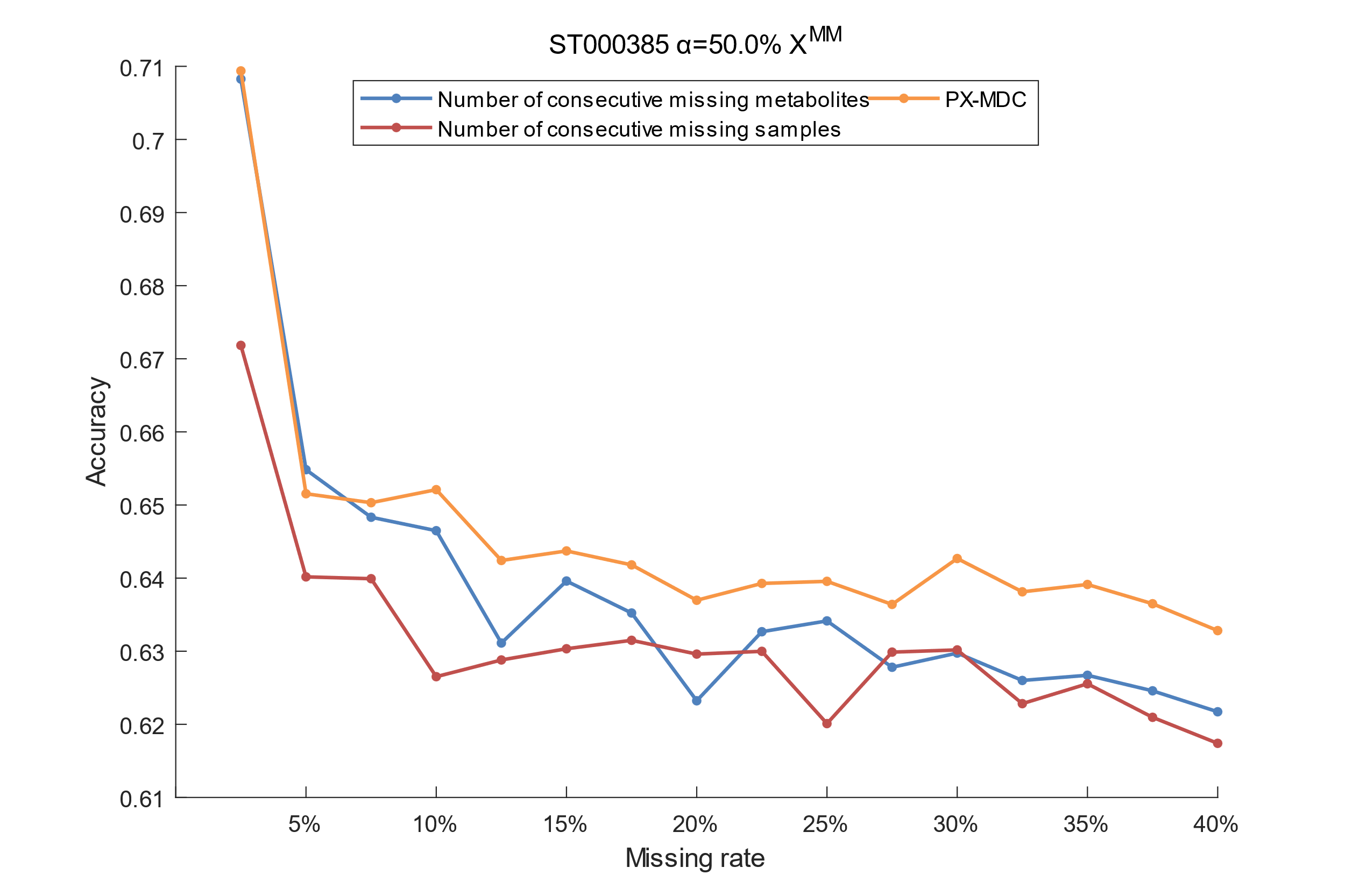 | 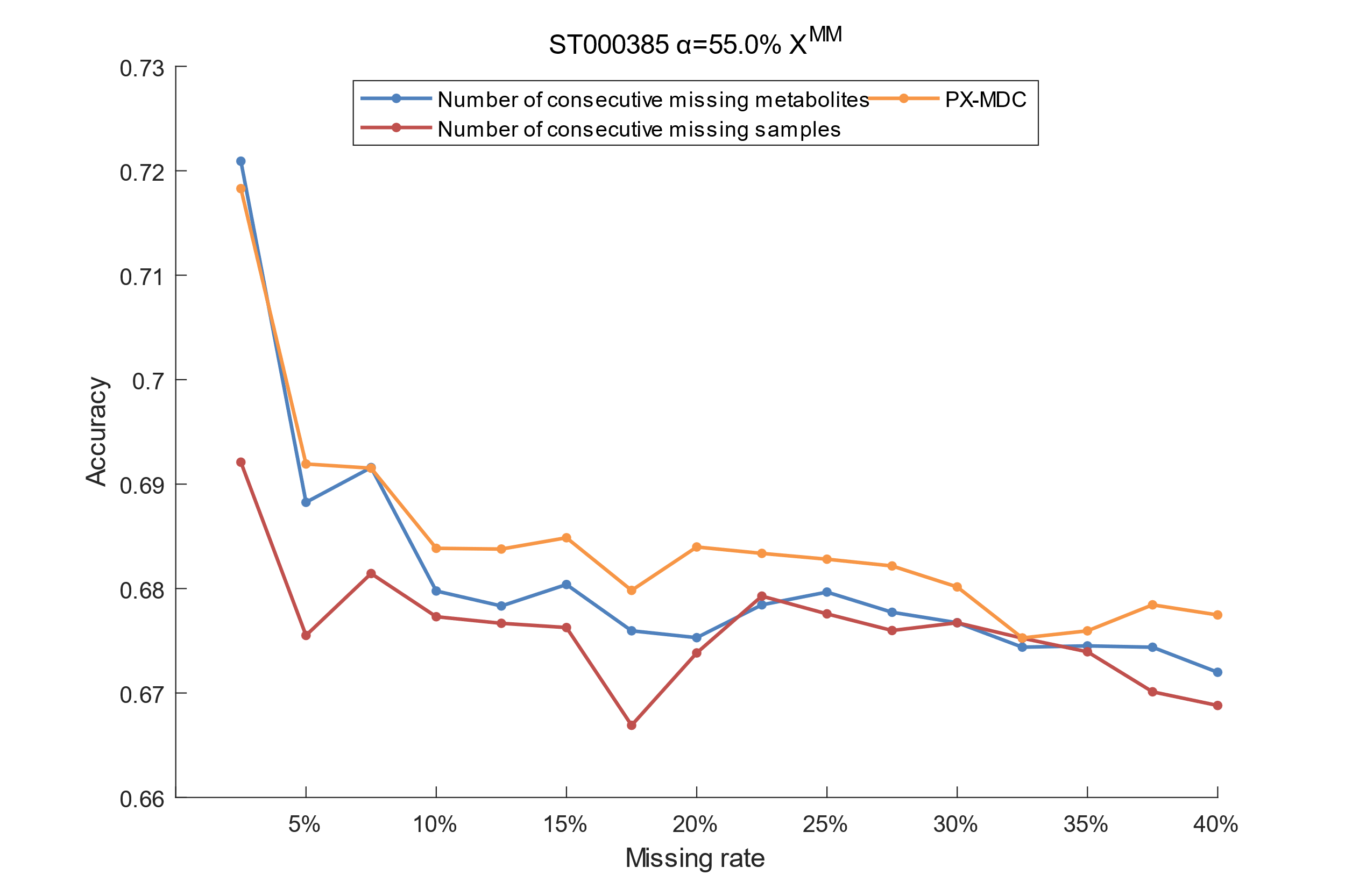 |
| 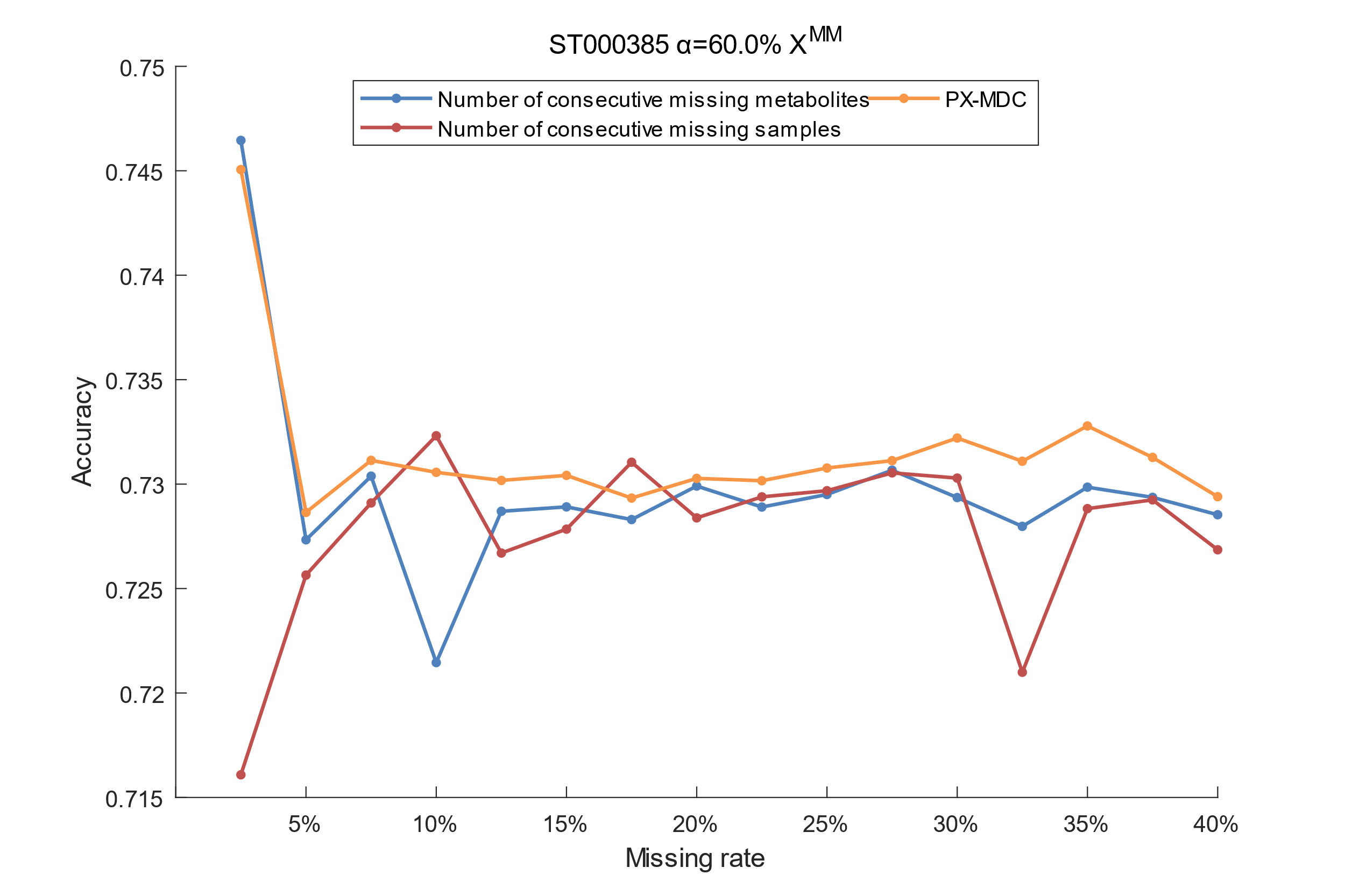 | 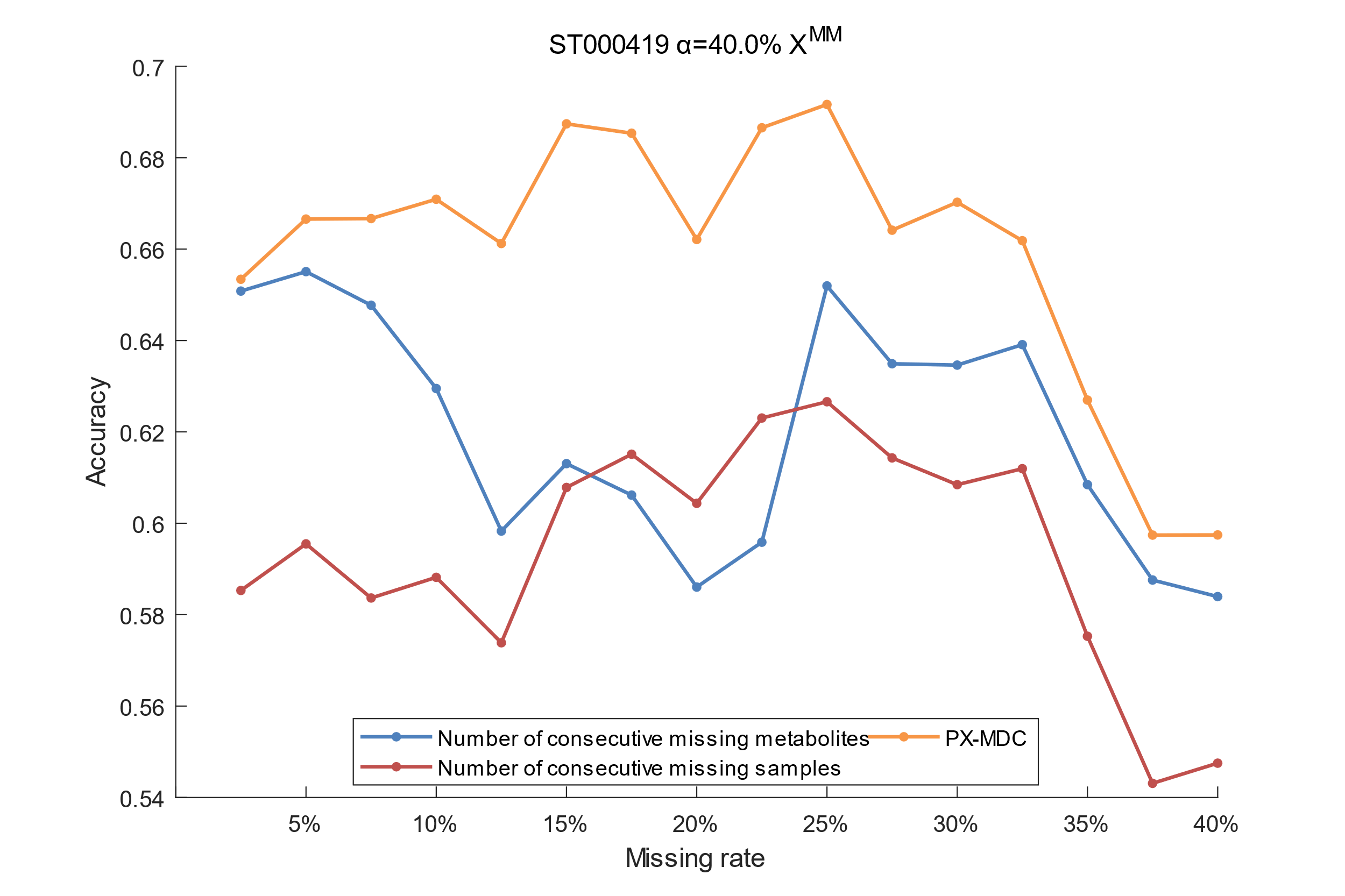 | 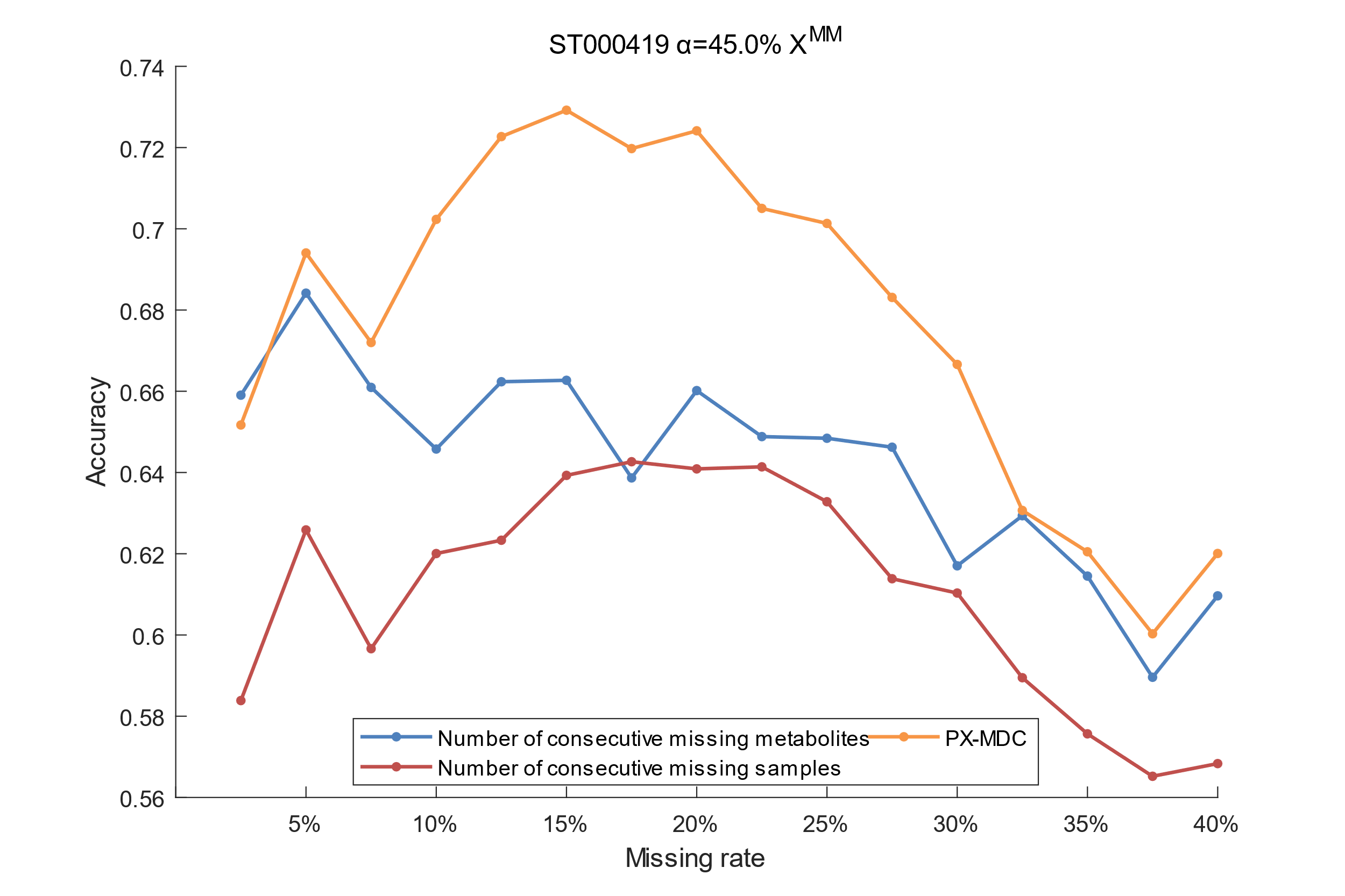 |
| 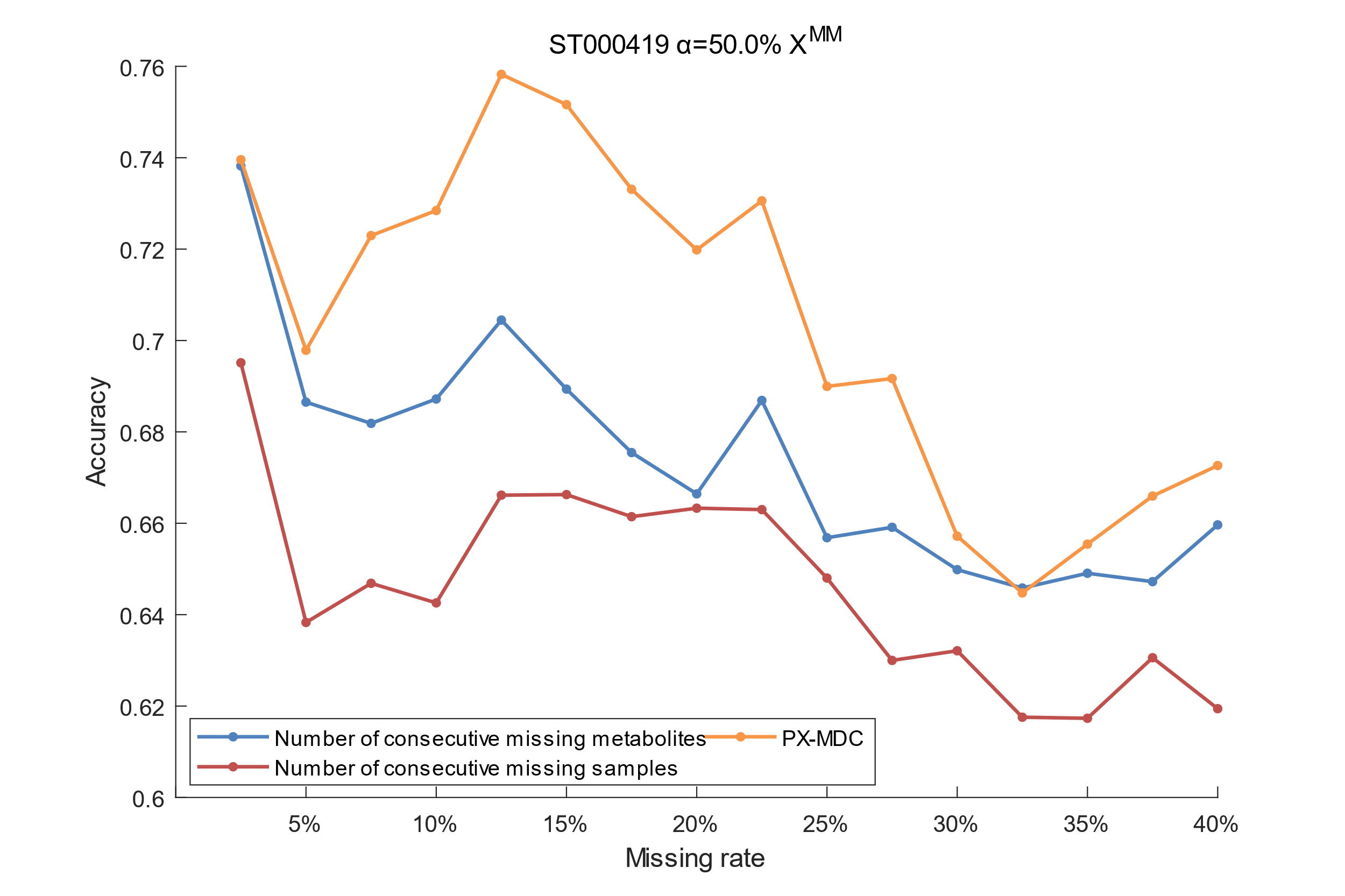 | 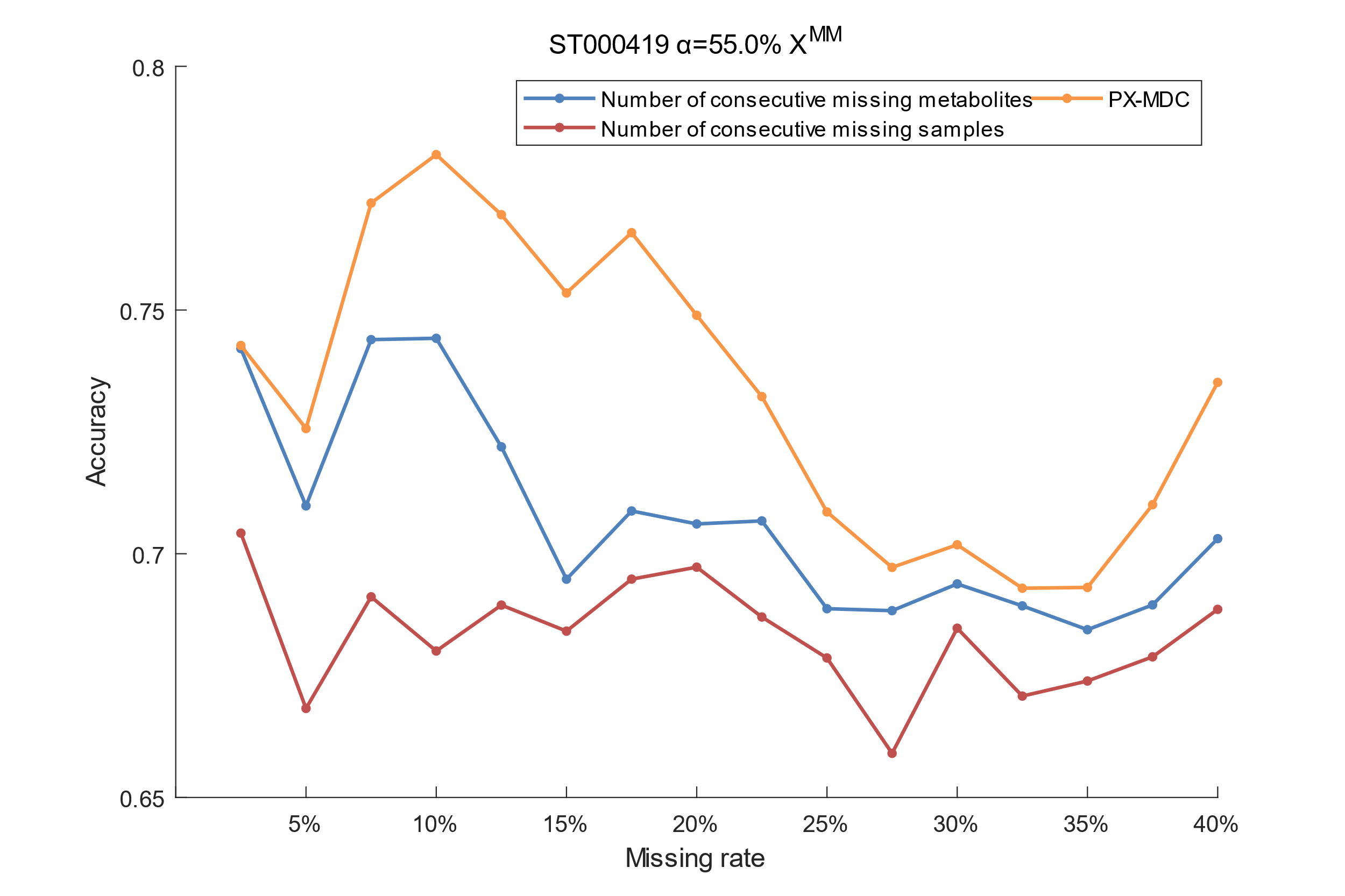 | 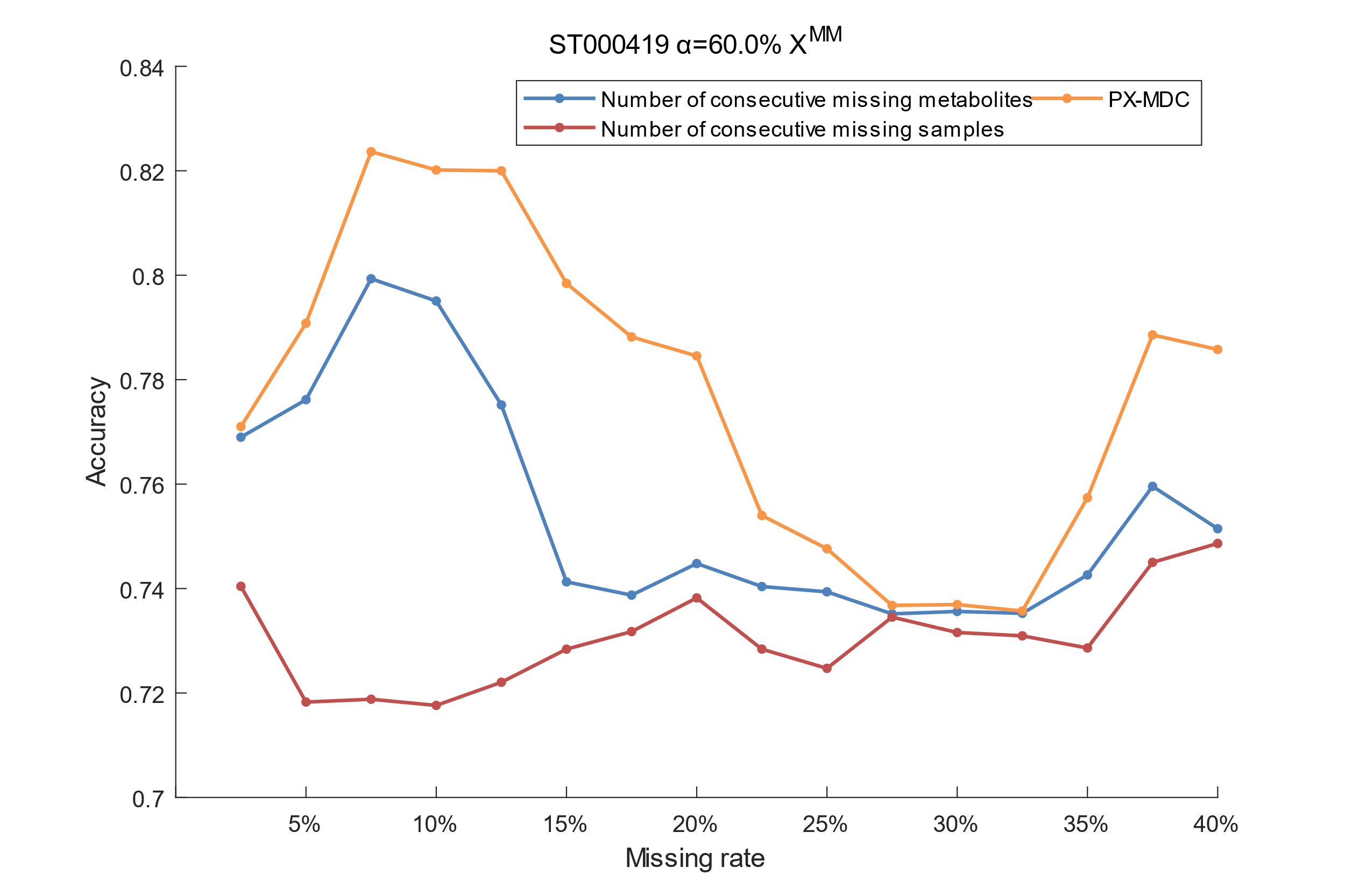 |

**Supplementary Figure 2.** Accuracy of X^MM^ in XGBoost model for different features.

| 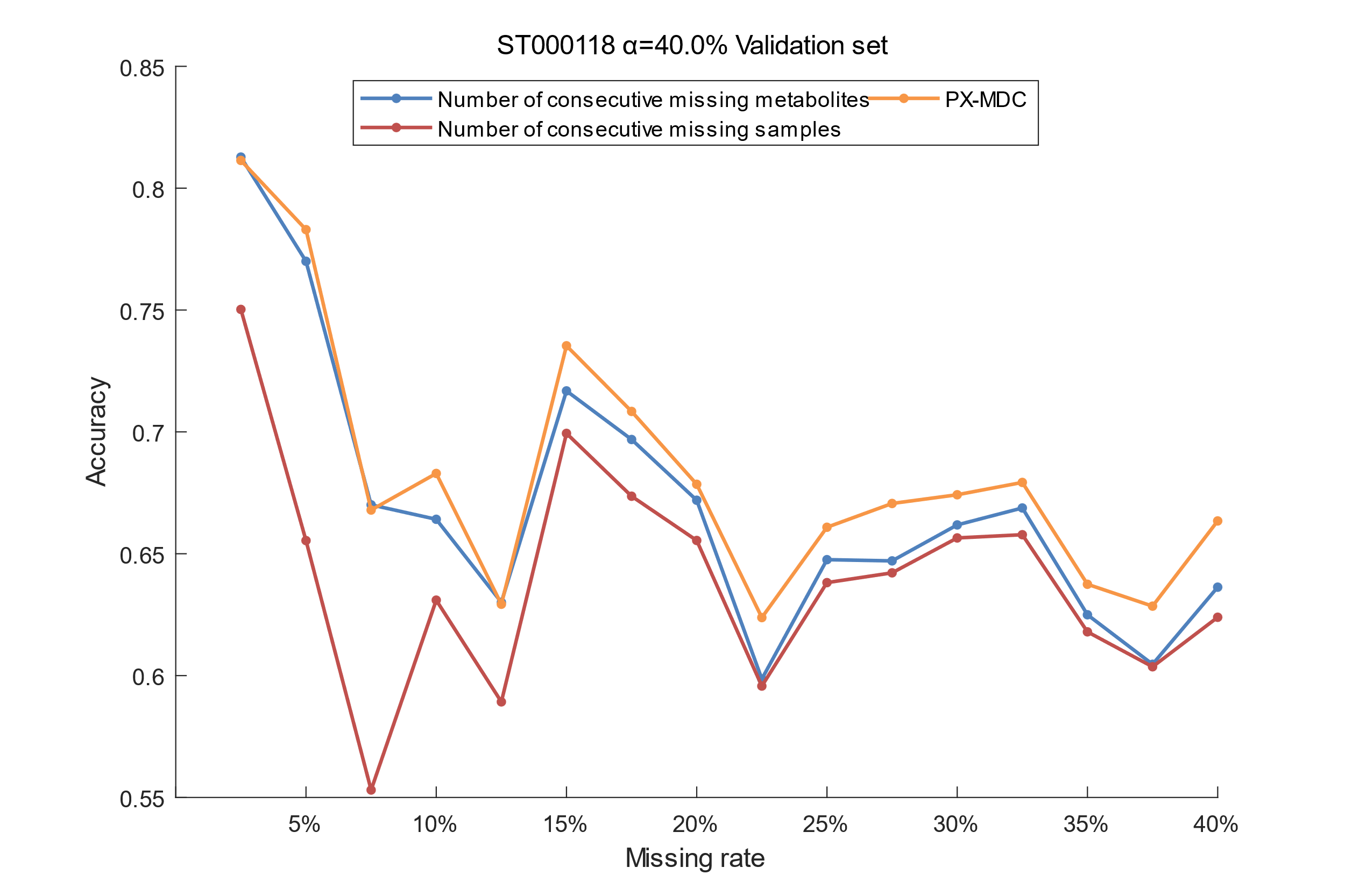 | 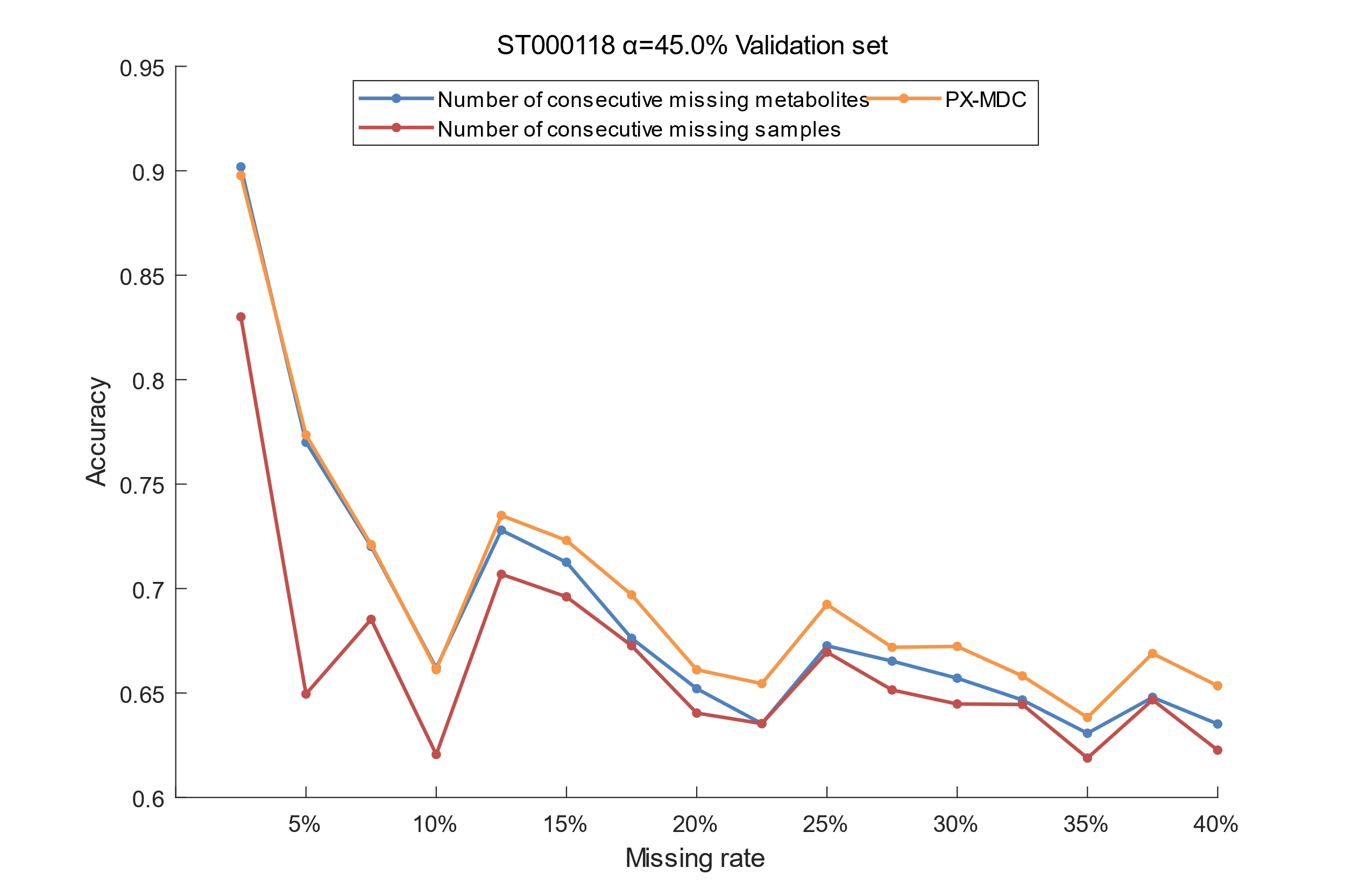 | 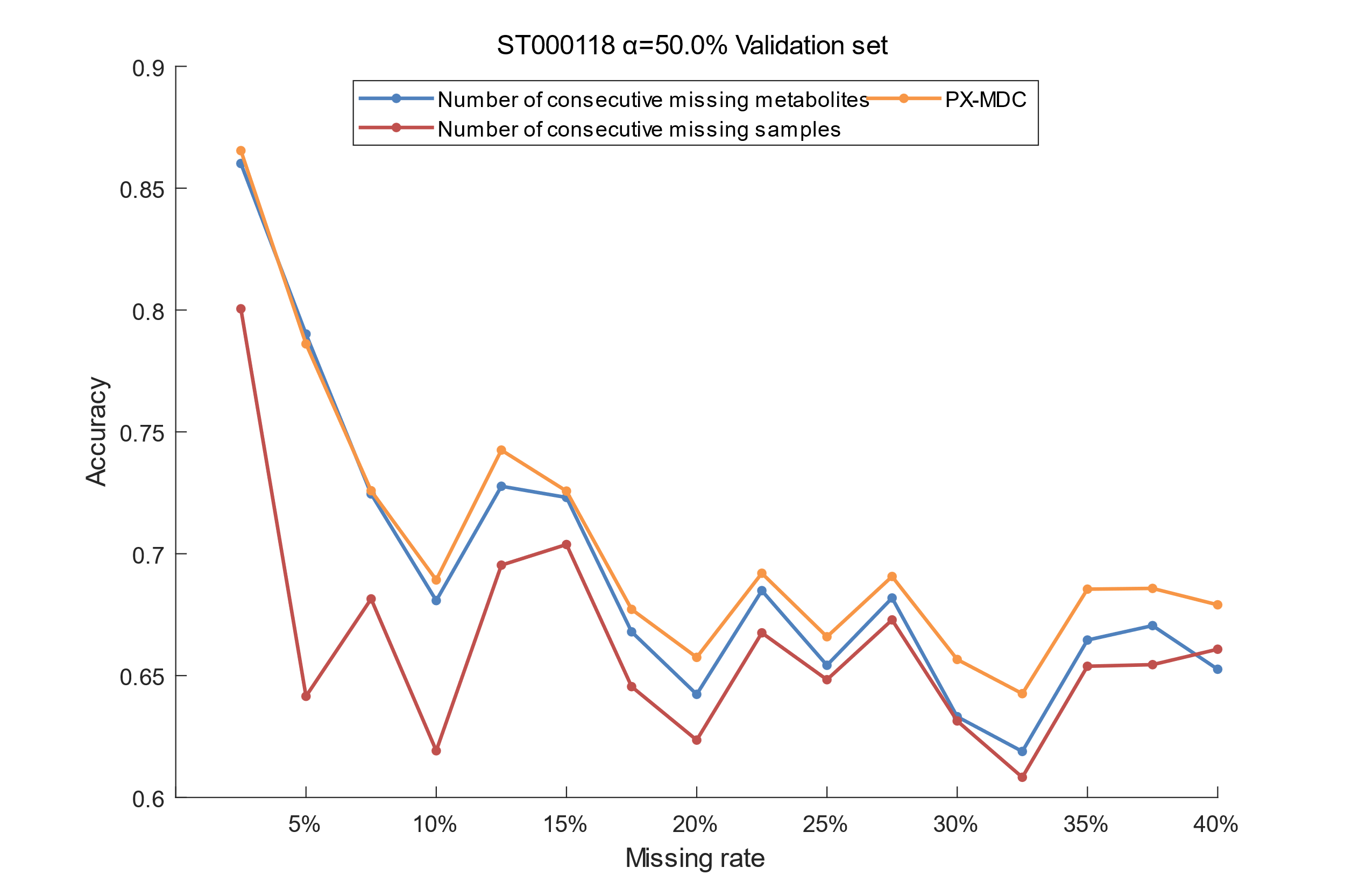 |
| --- | --- | --- |
| 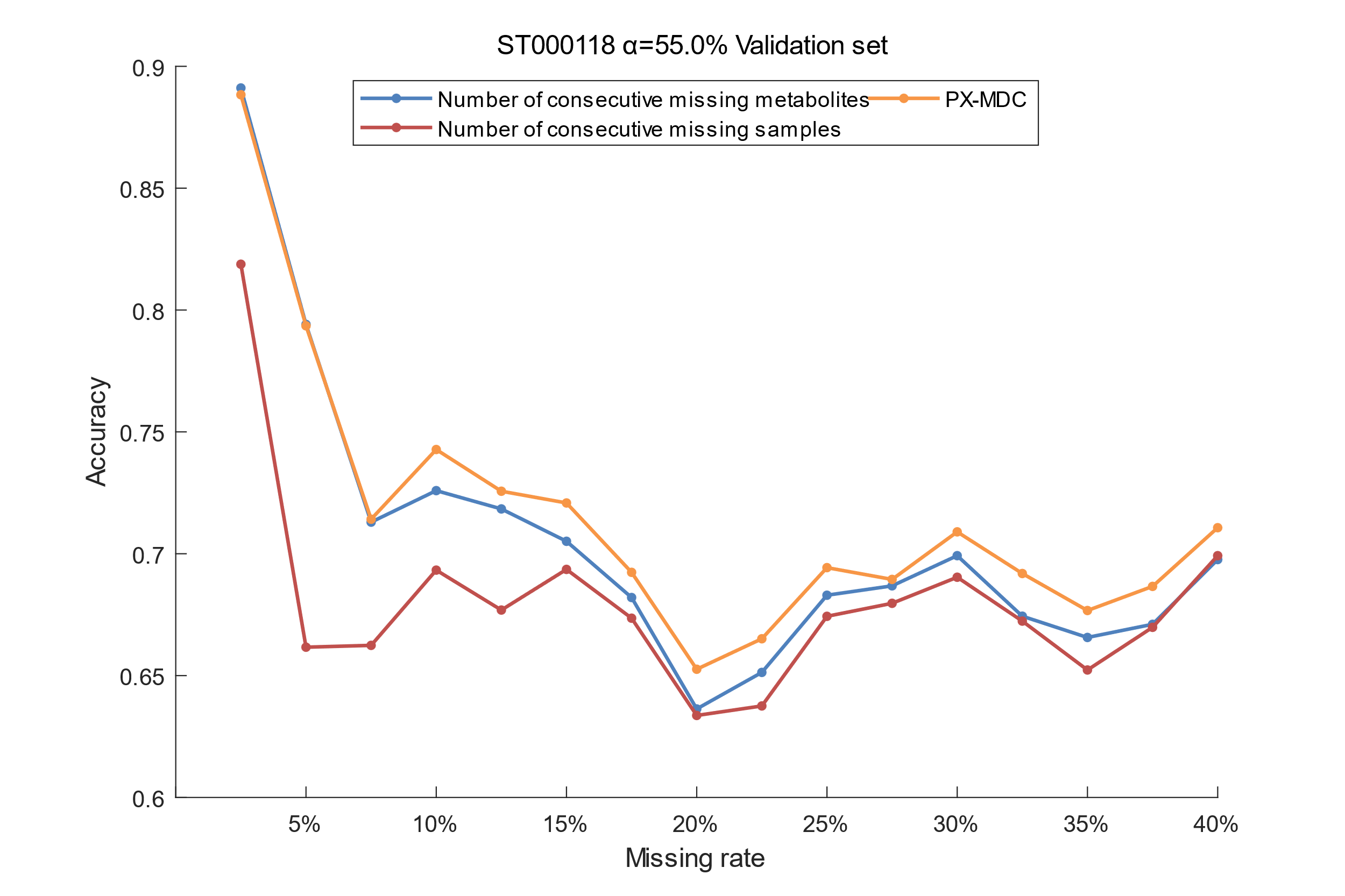 | 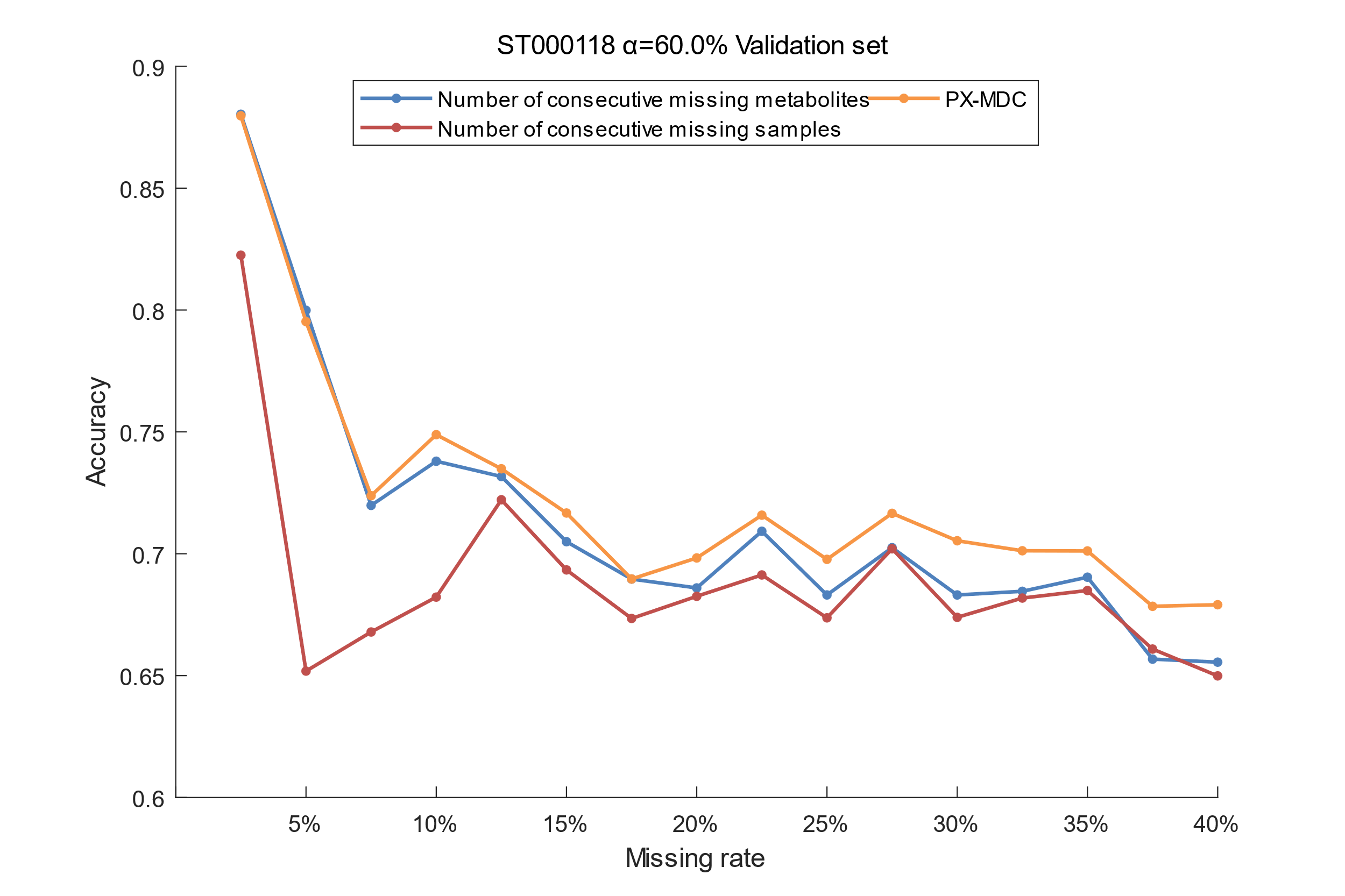 | 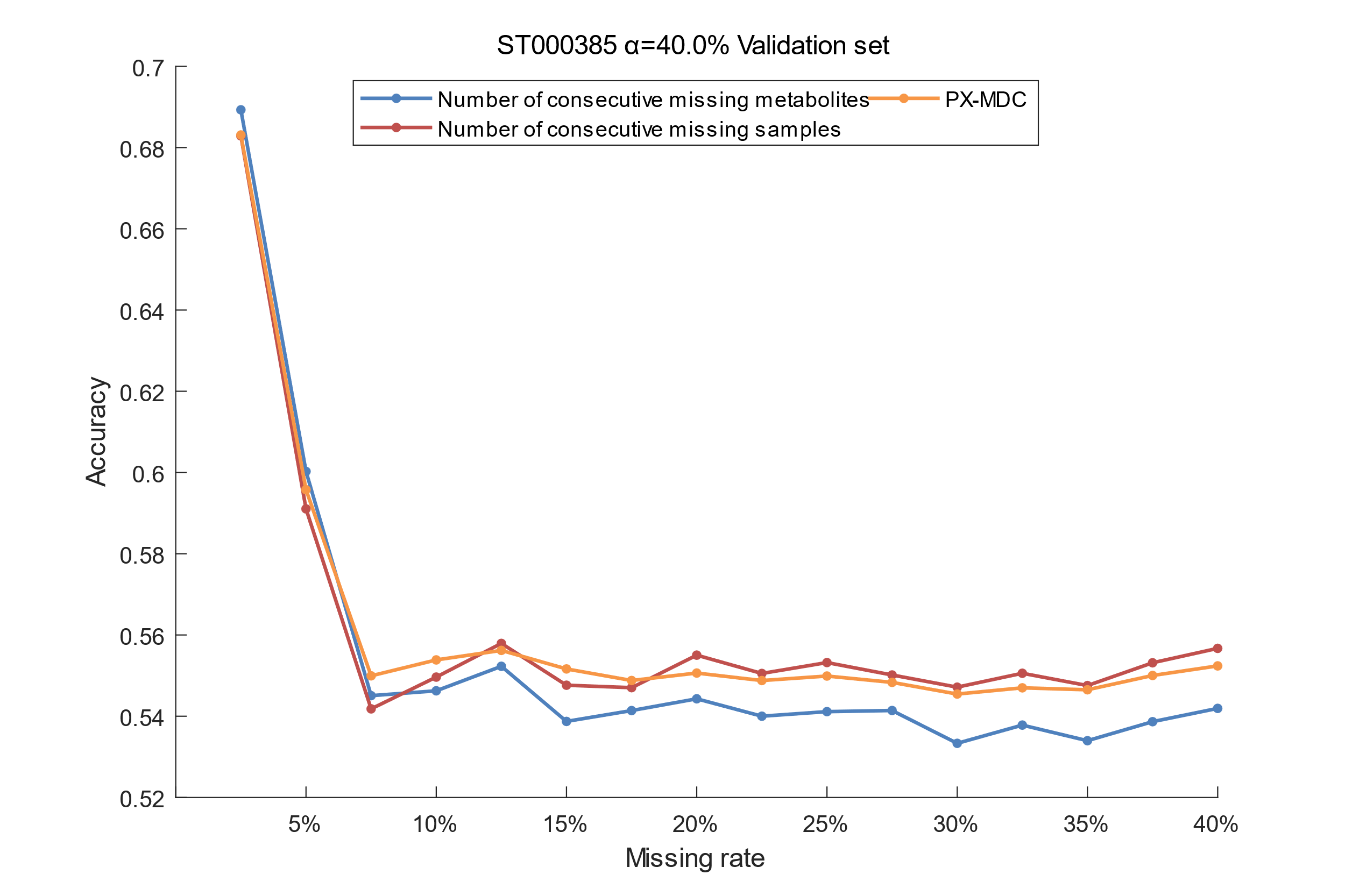 |
| 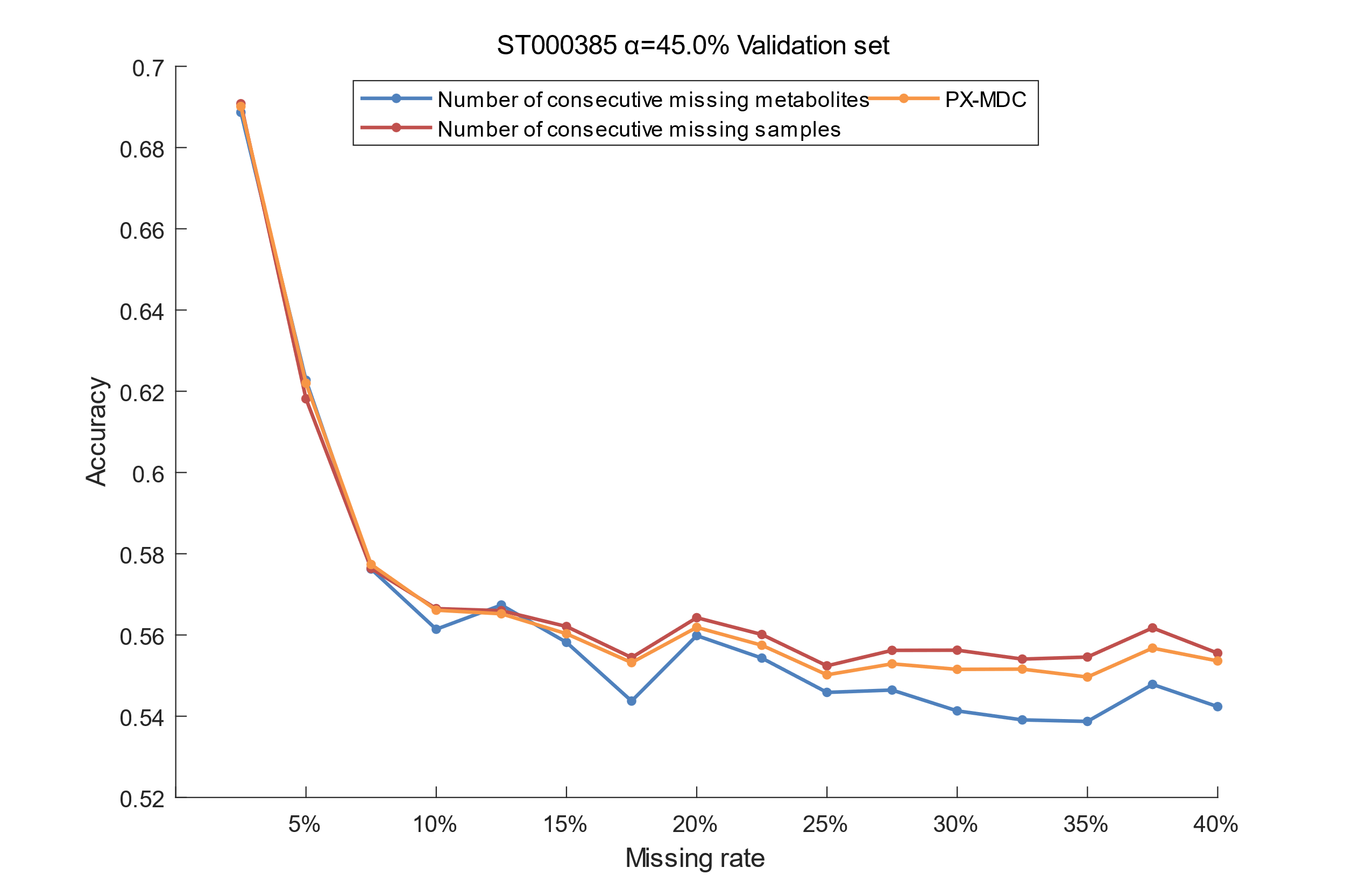 | 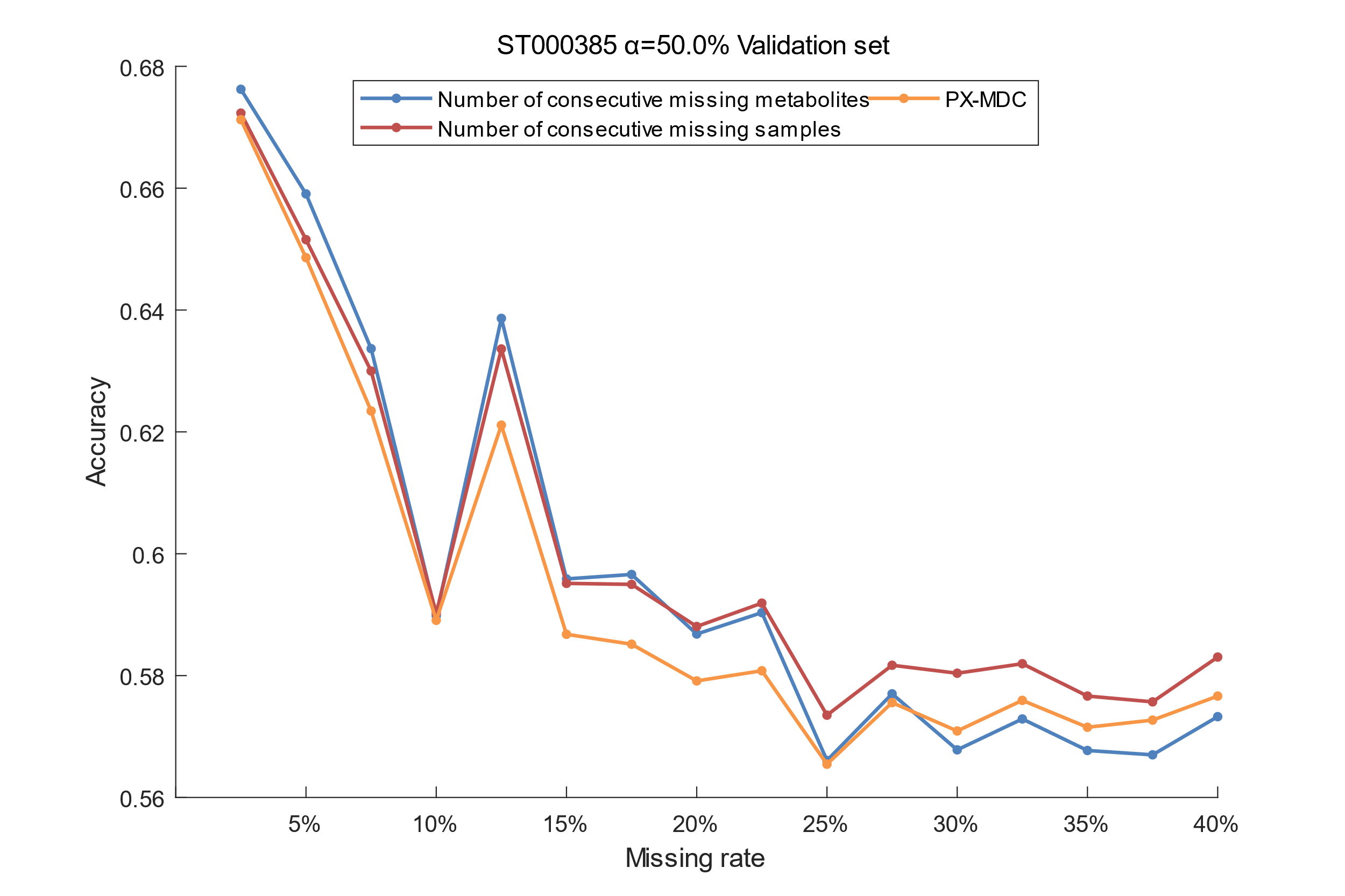 | 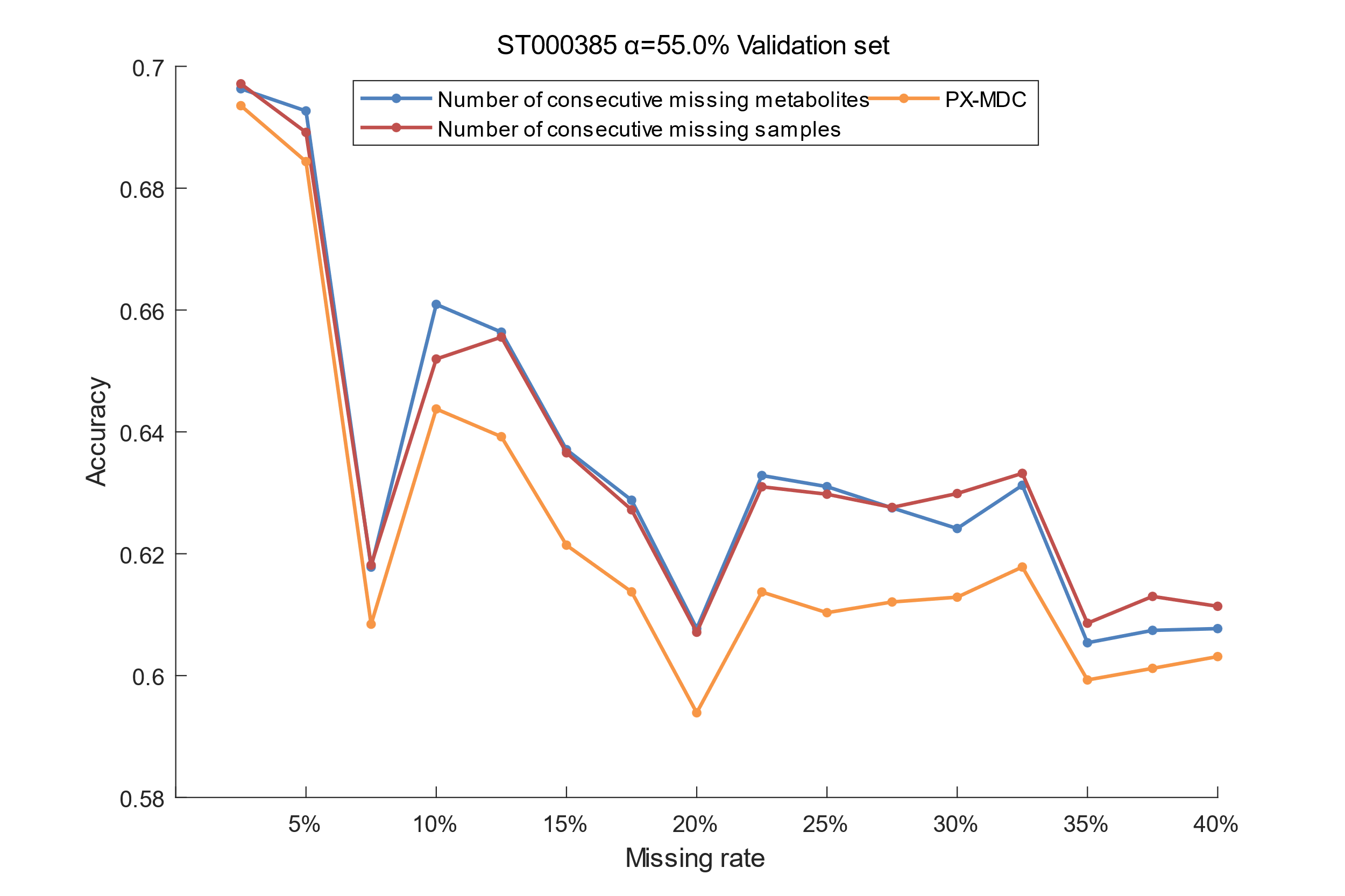 |
| 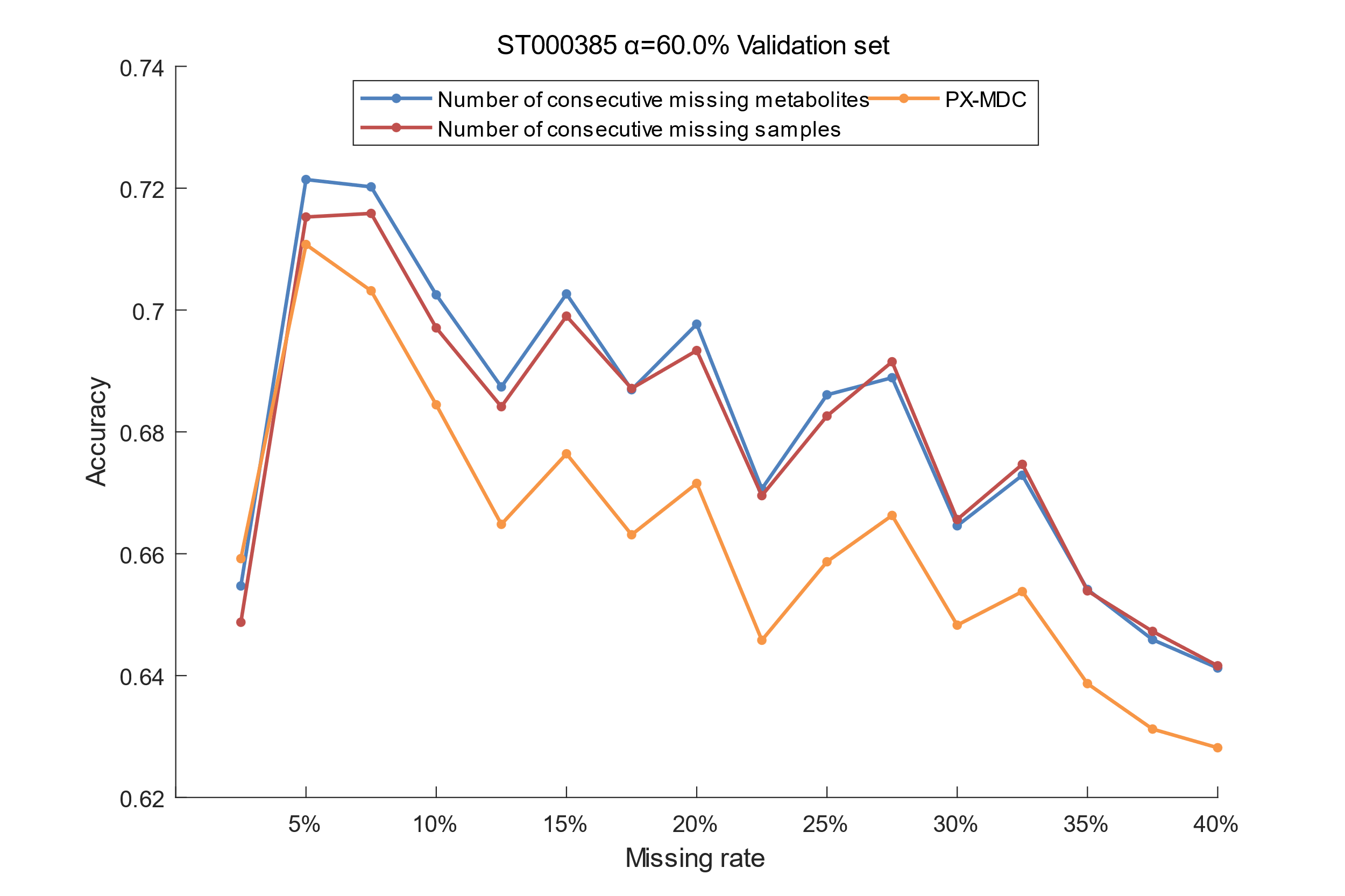 | 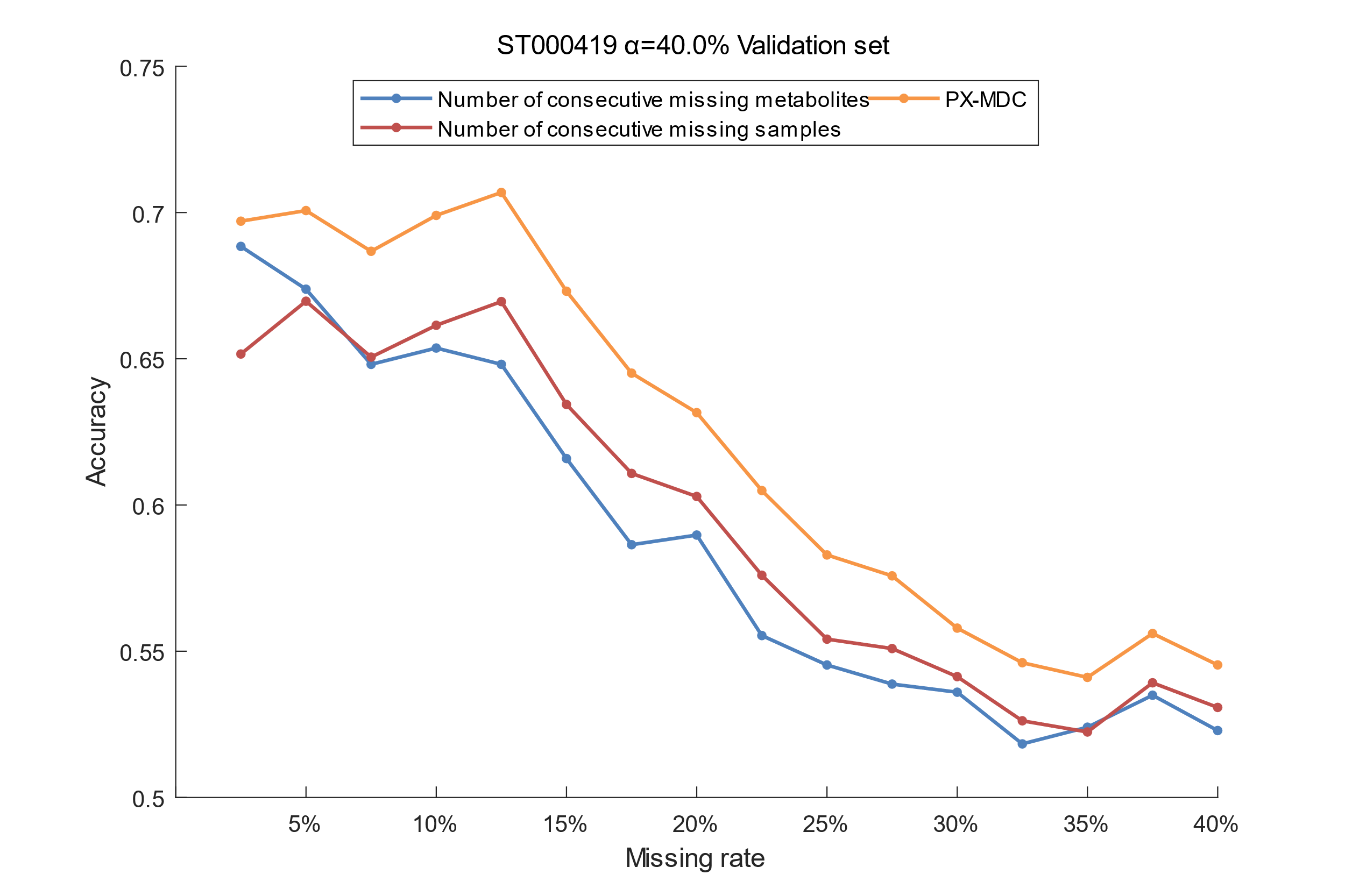 | 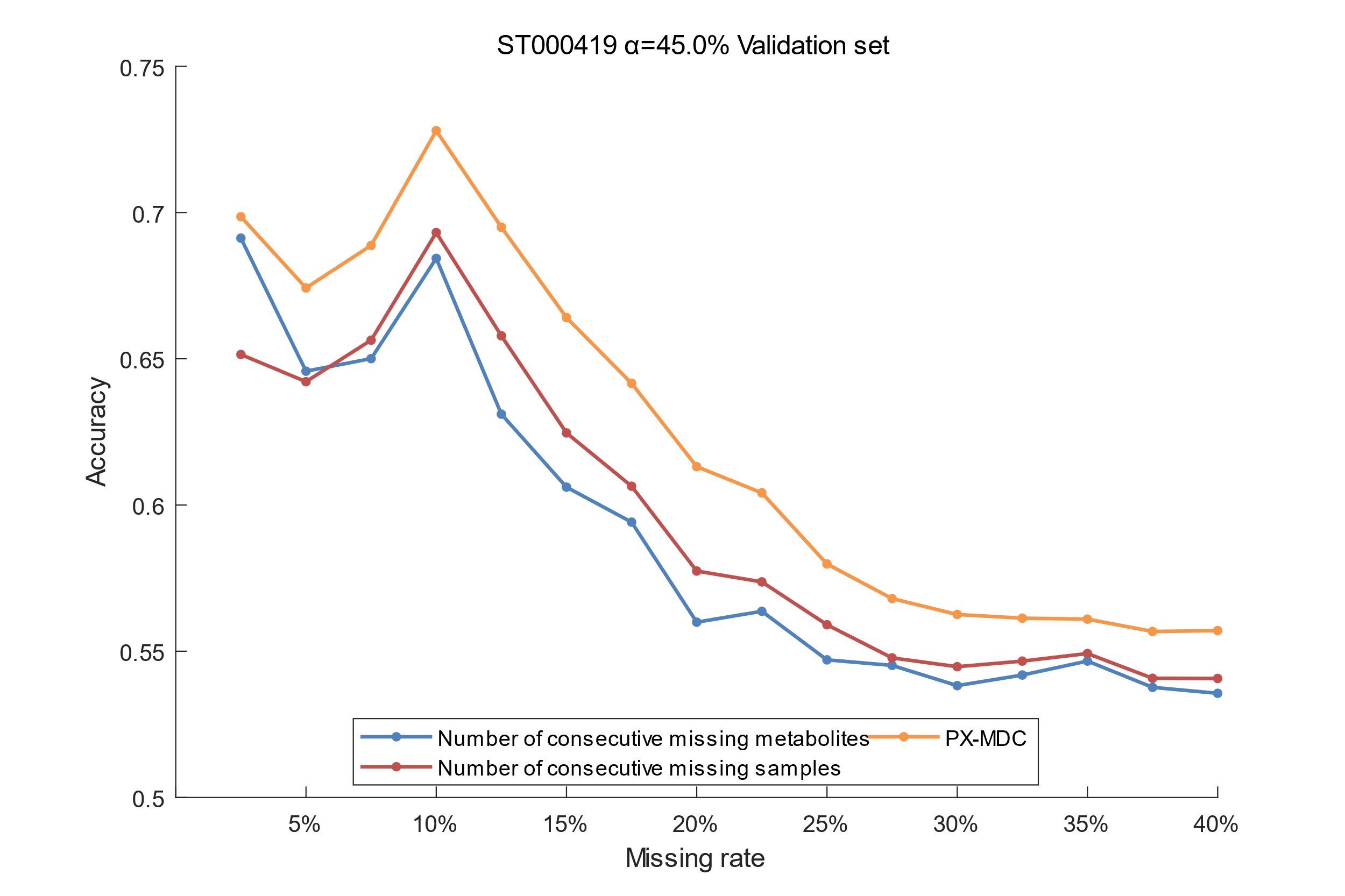 |
| 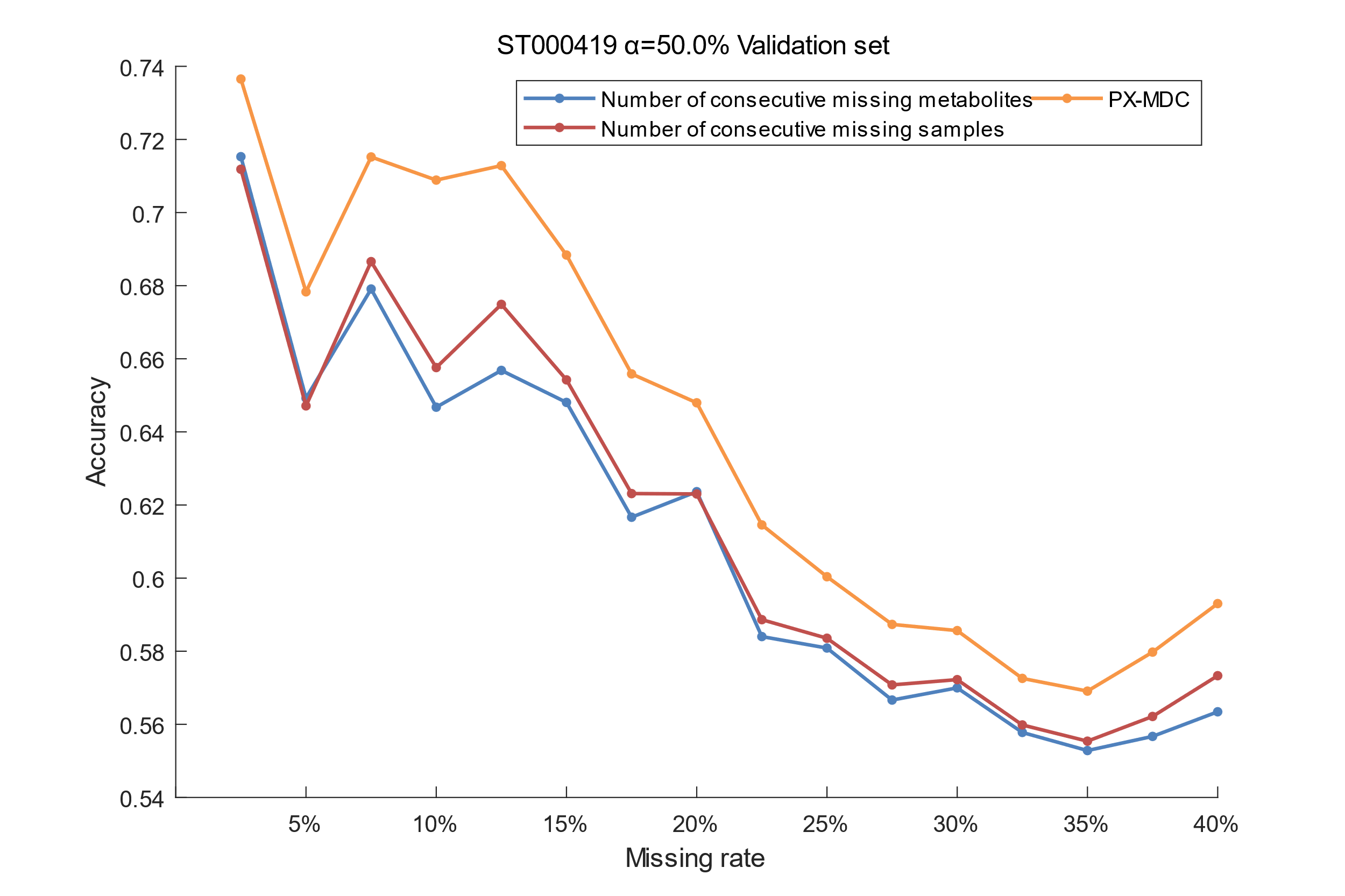 | 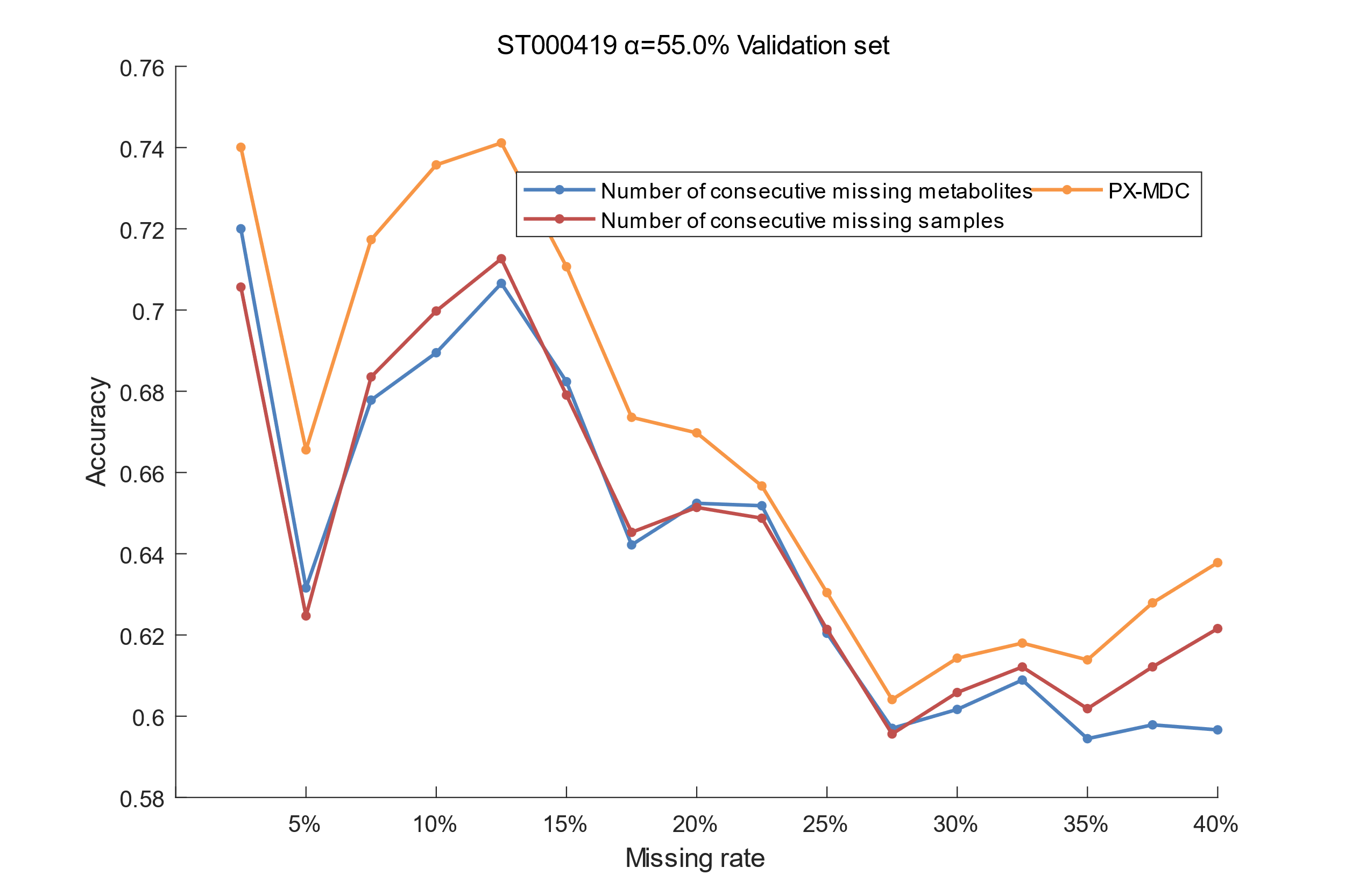 | 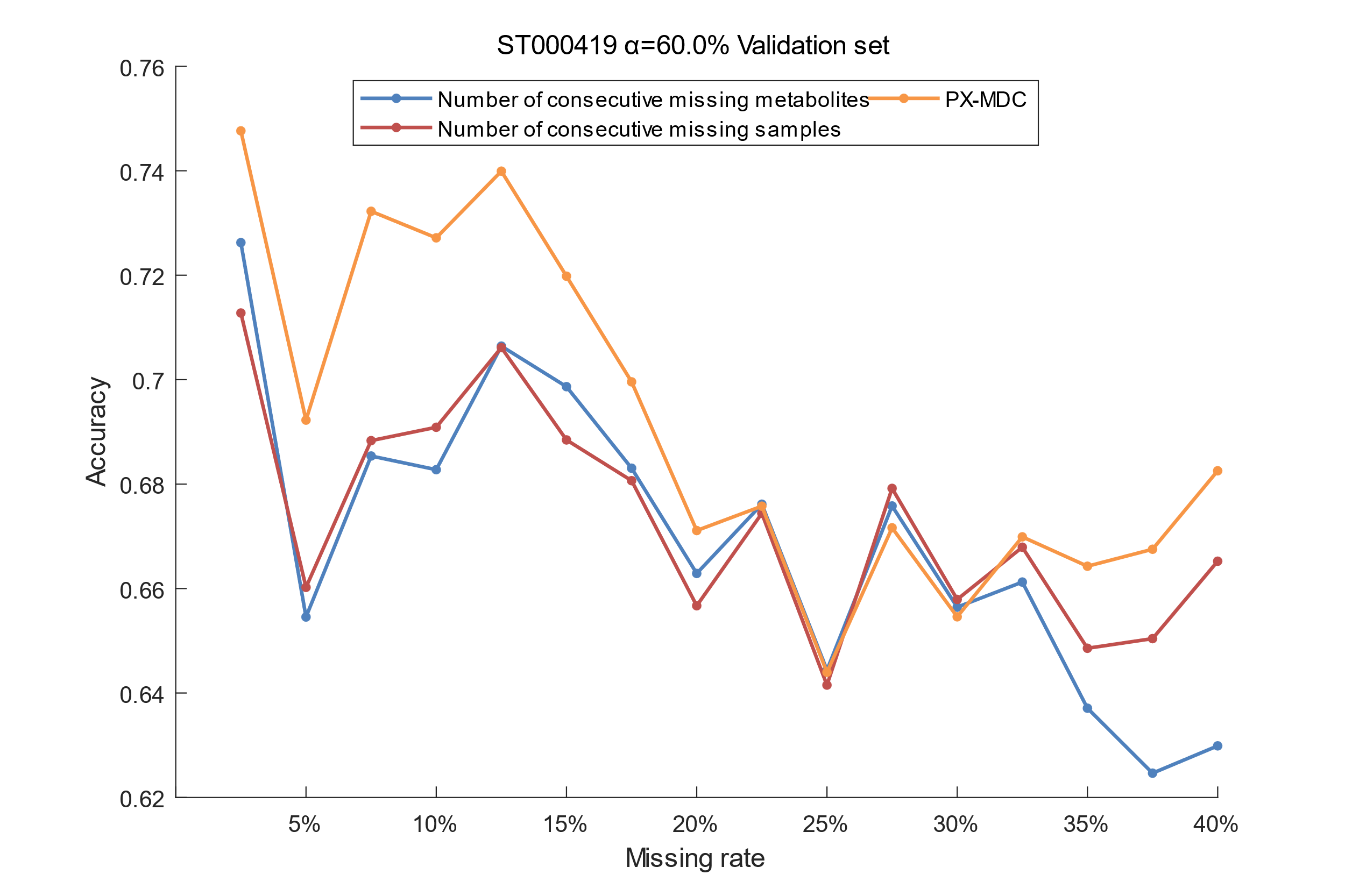 |

**Supplementary Figure 3.** Accuracy of validation sets in RF models for different feature.

| 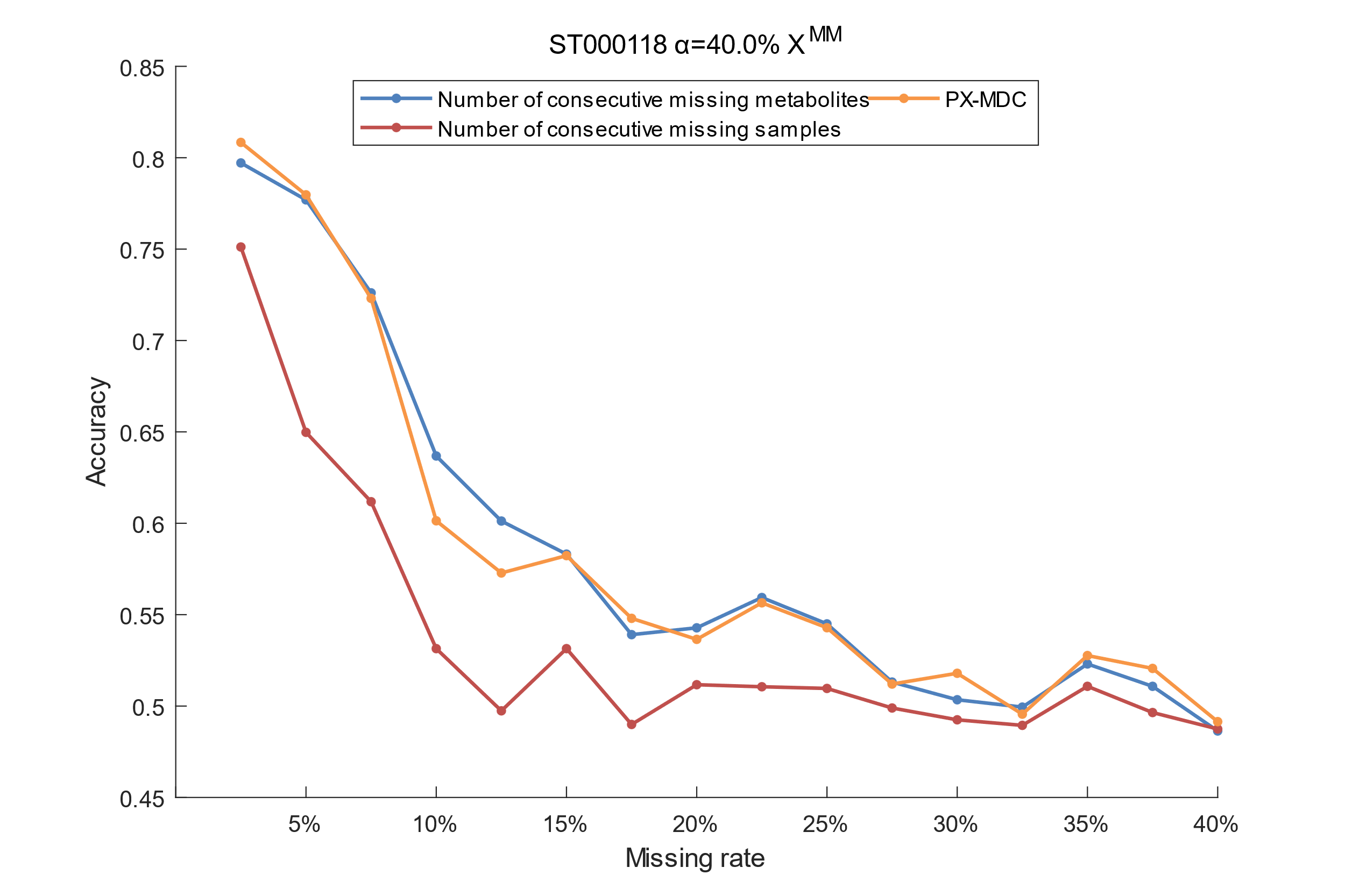 | 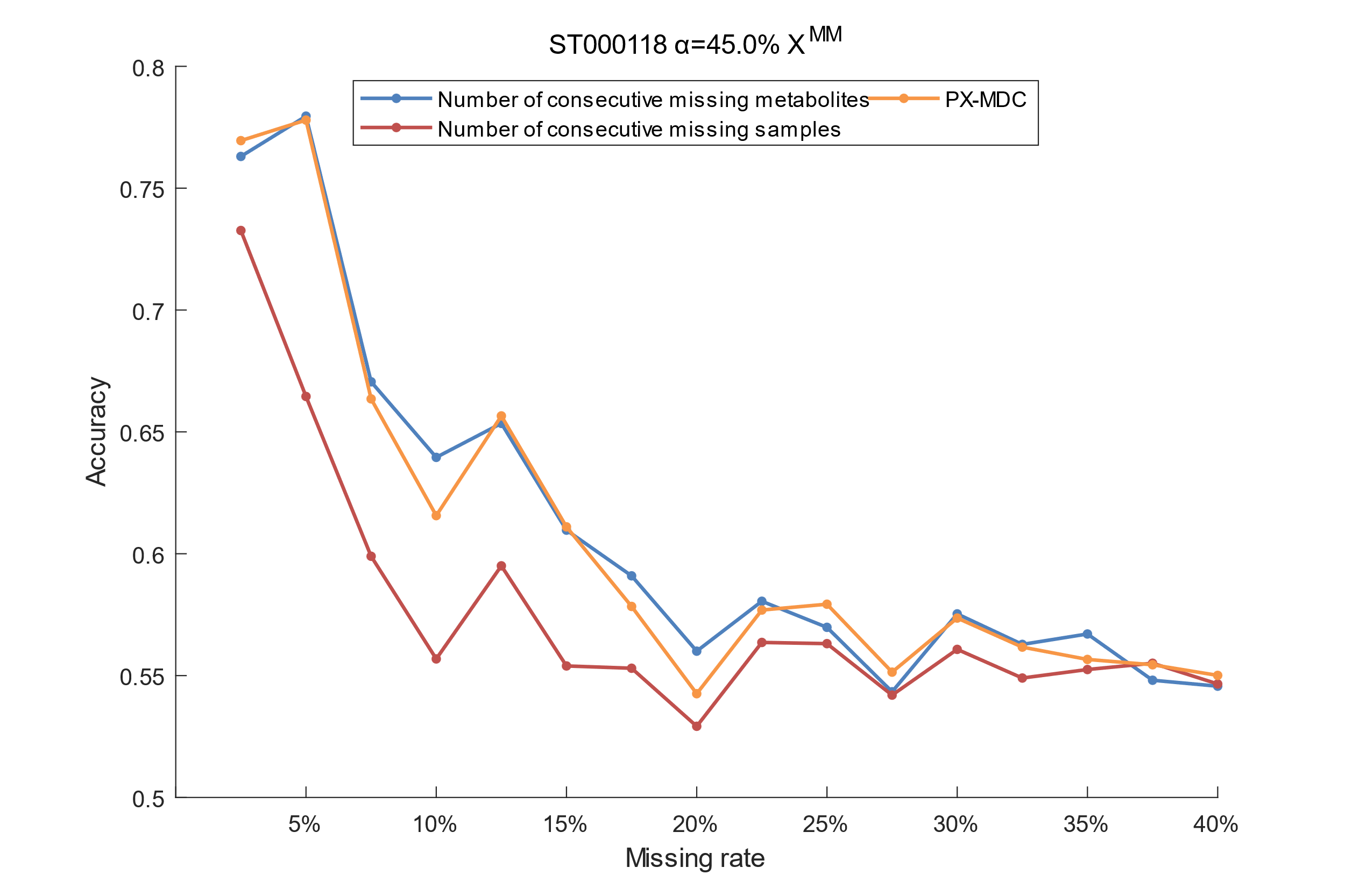 | 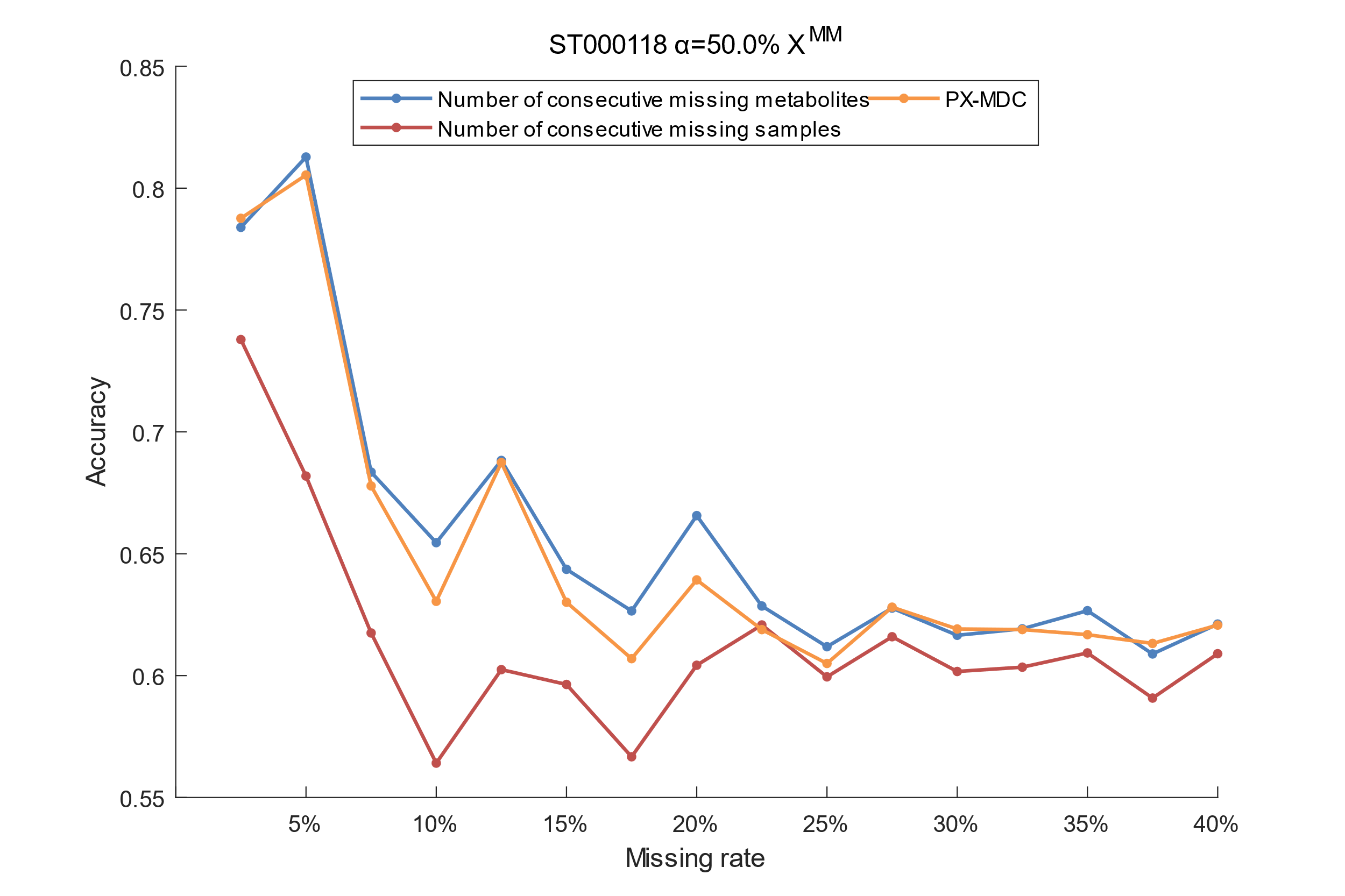 |
| --- | --- | --- |
| 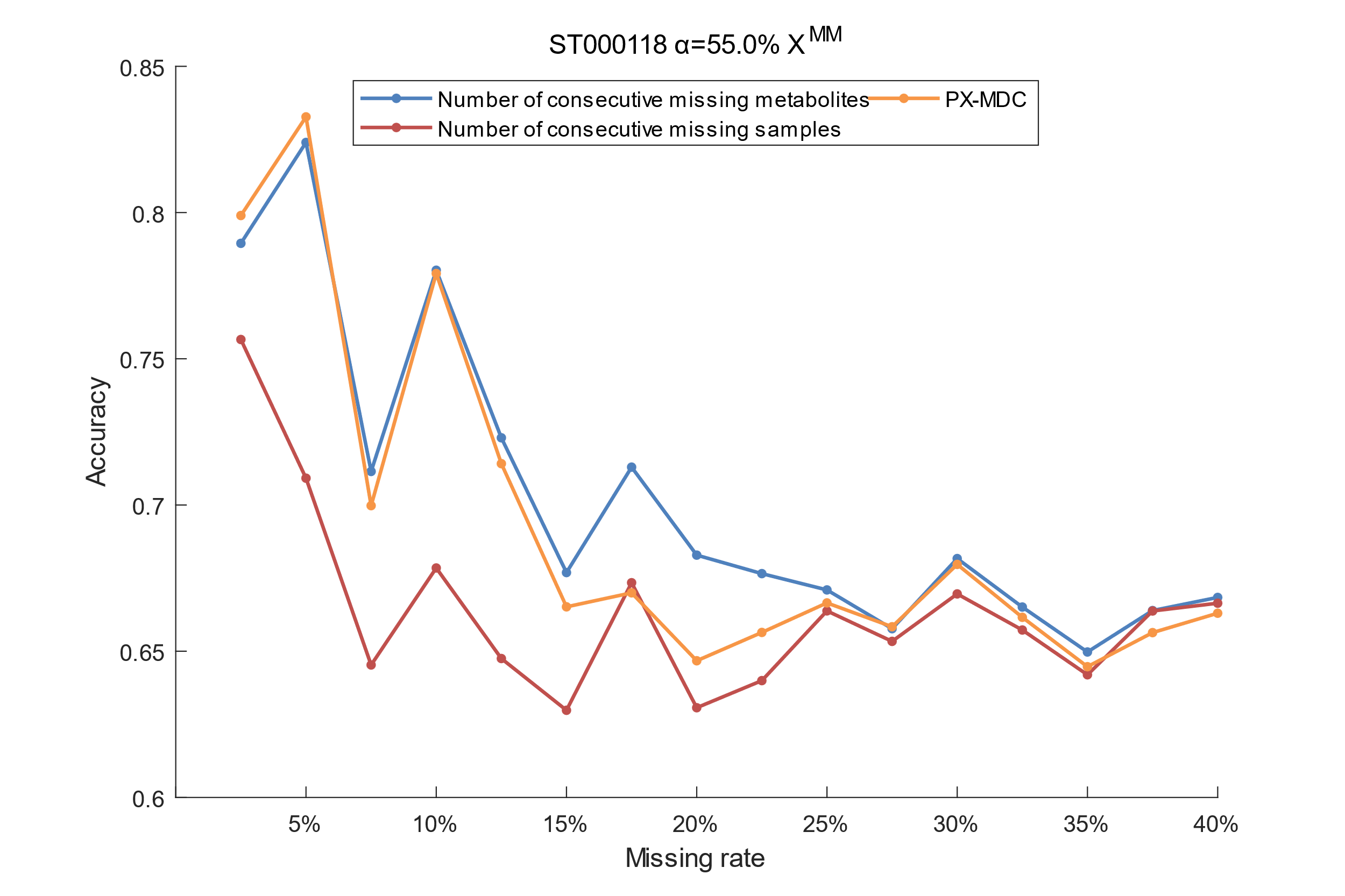 | 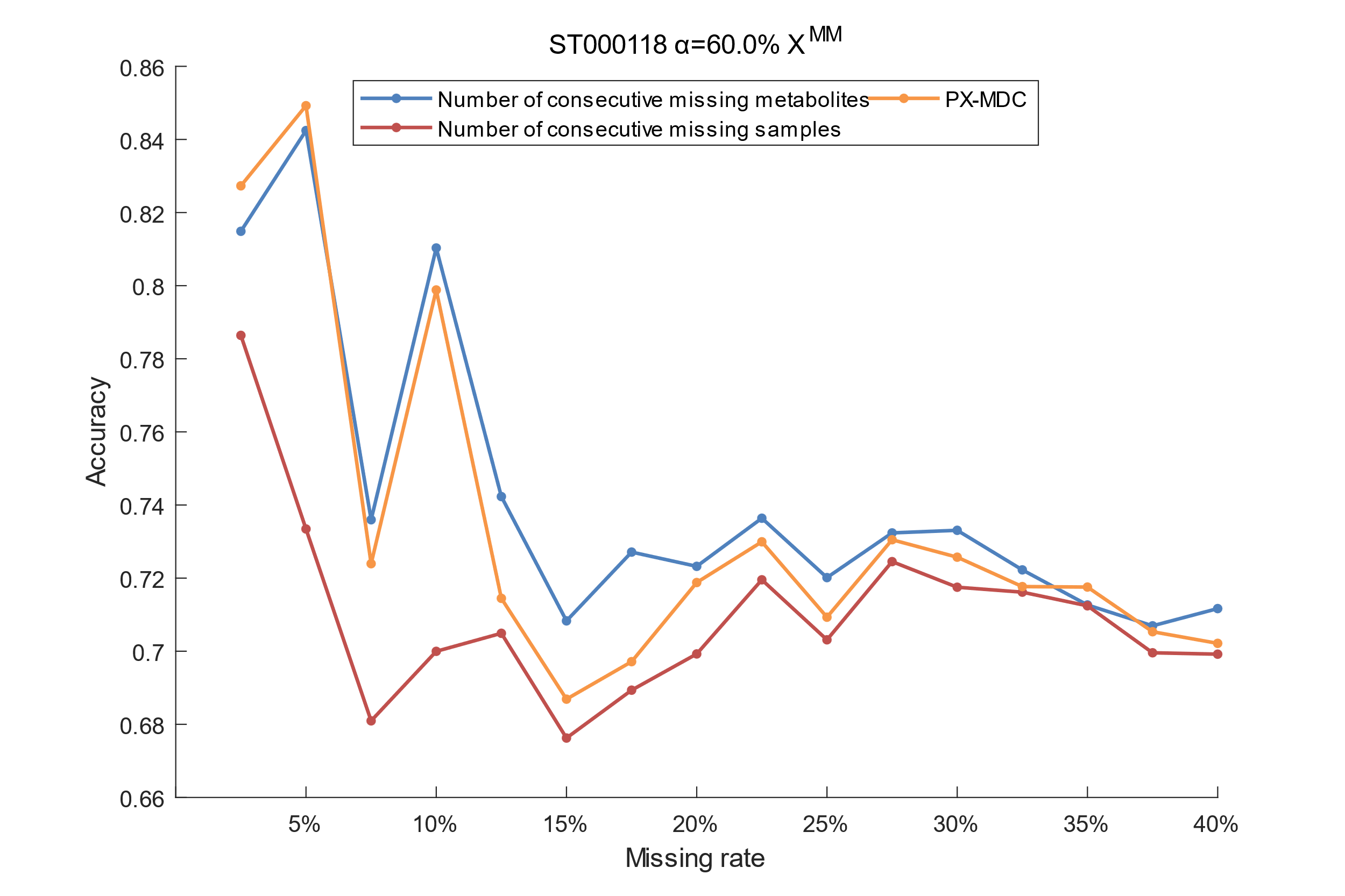 | 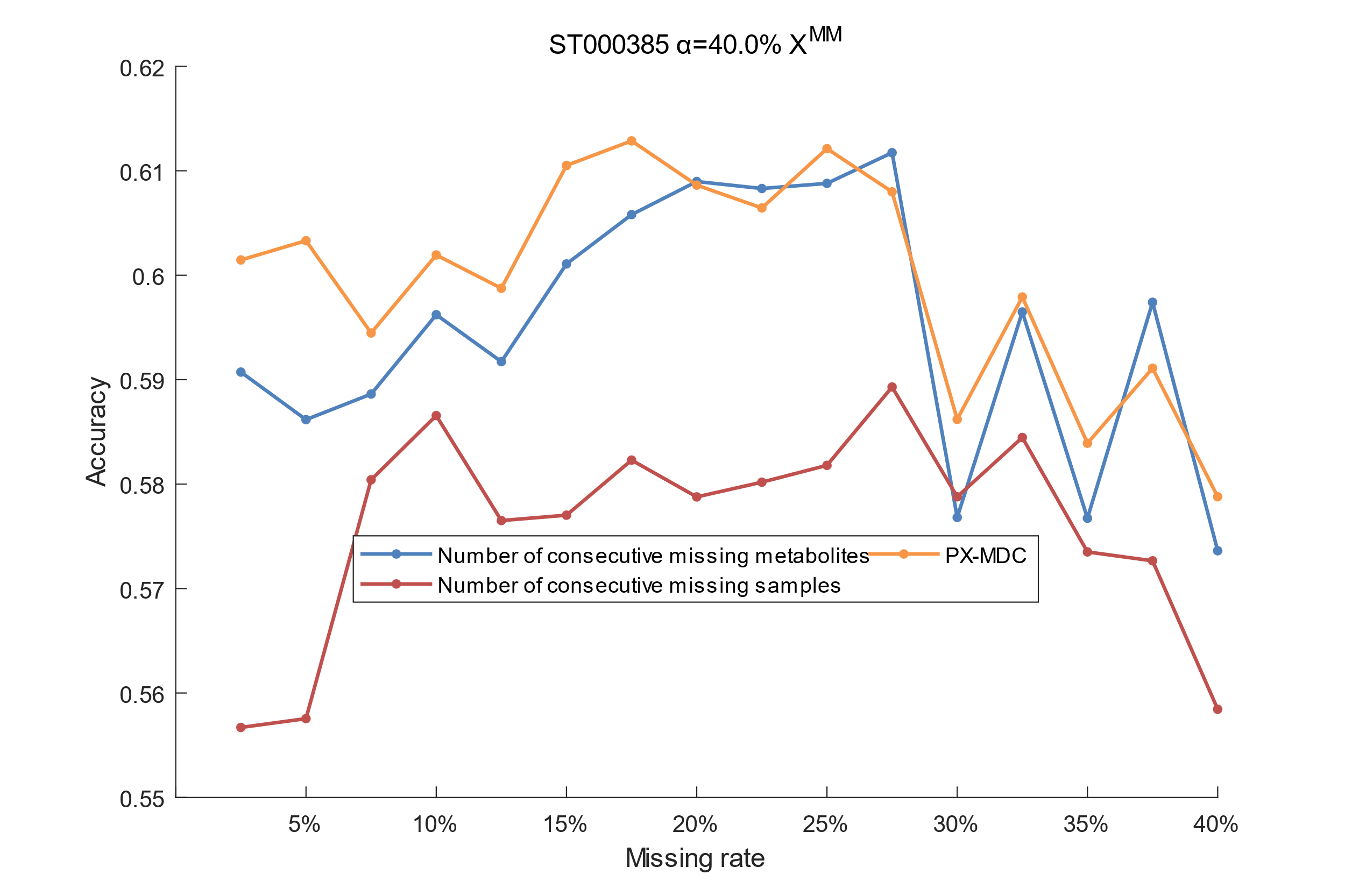 |
| 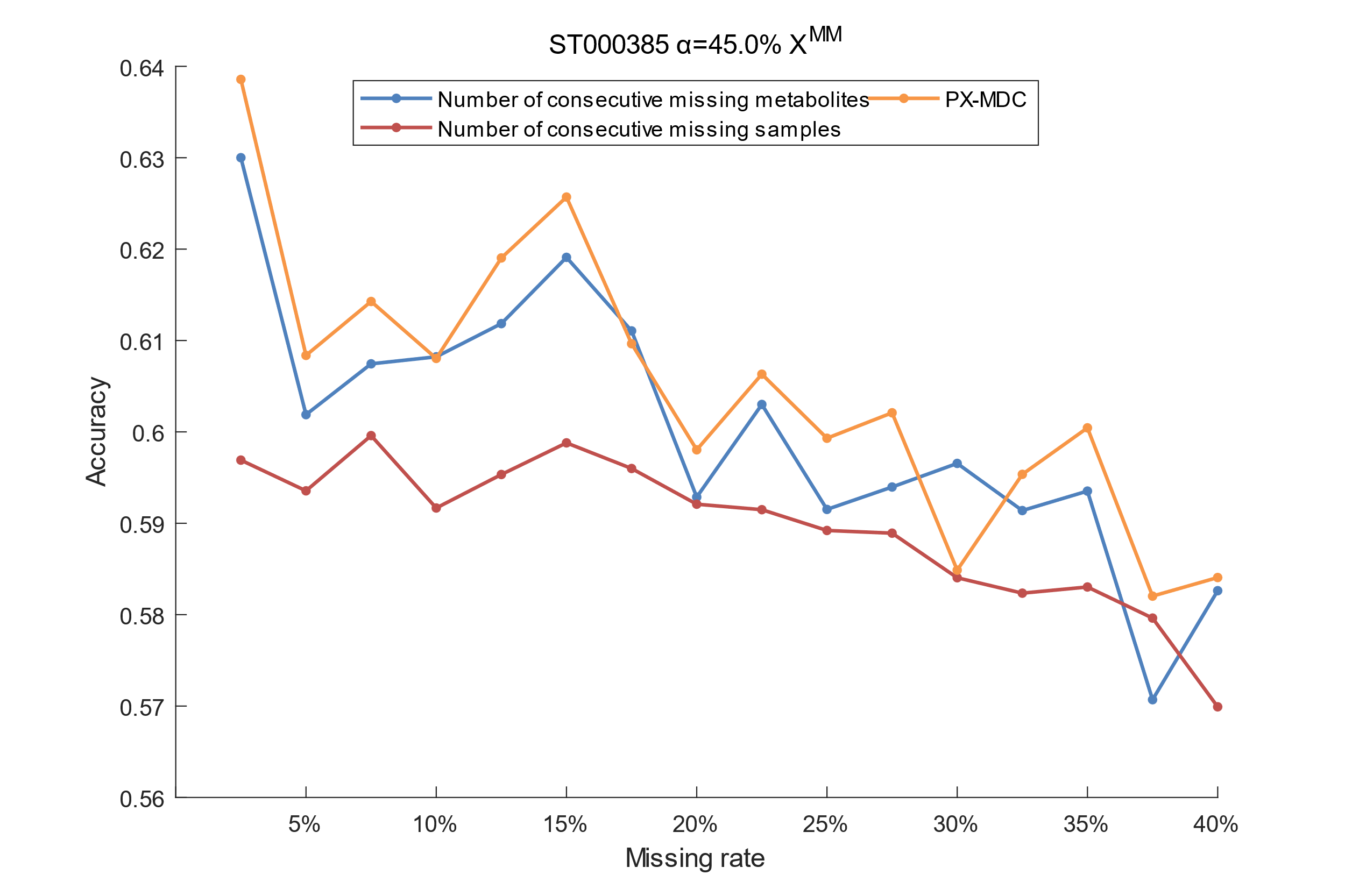 | 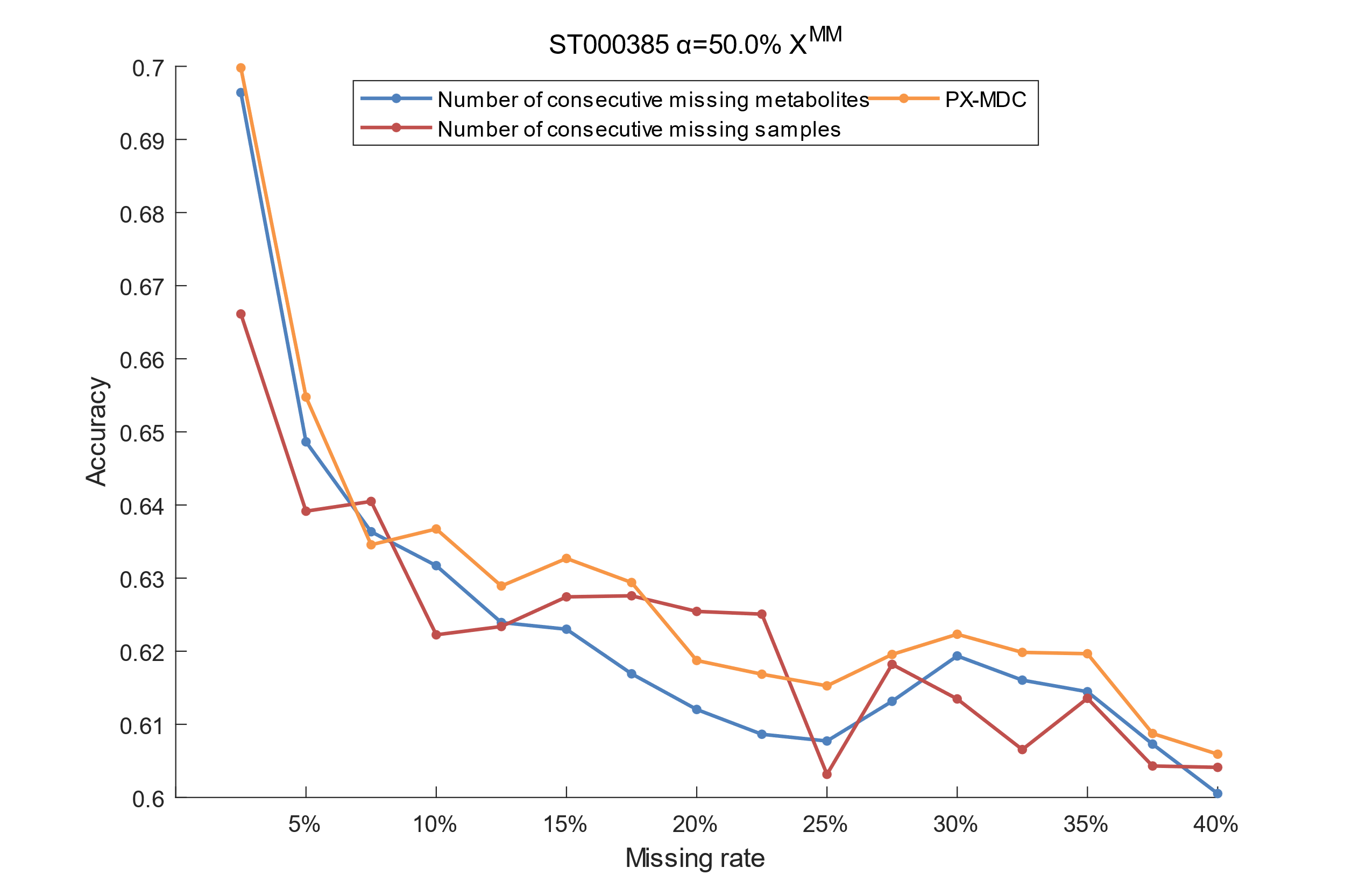 | 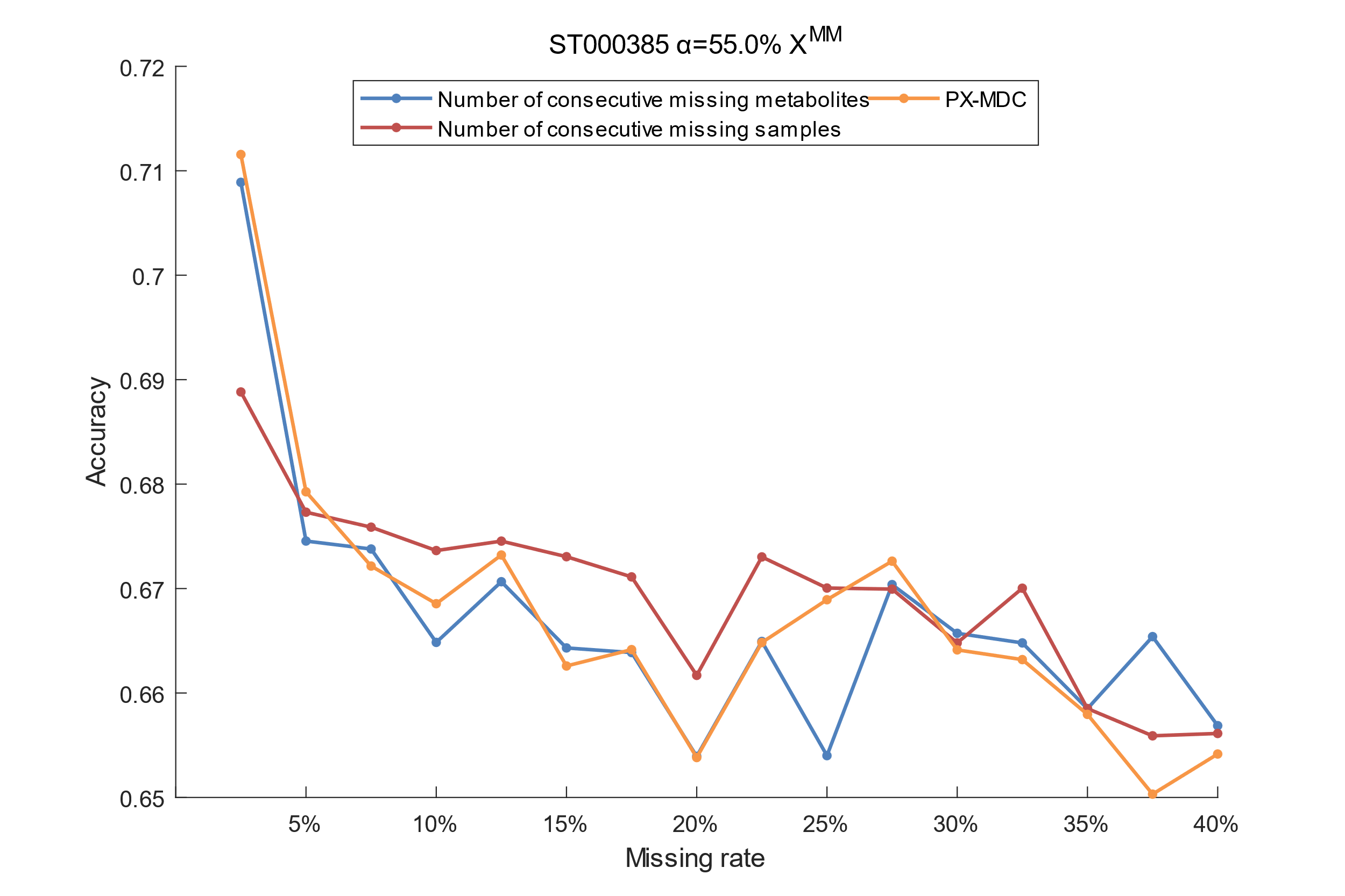 |
| 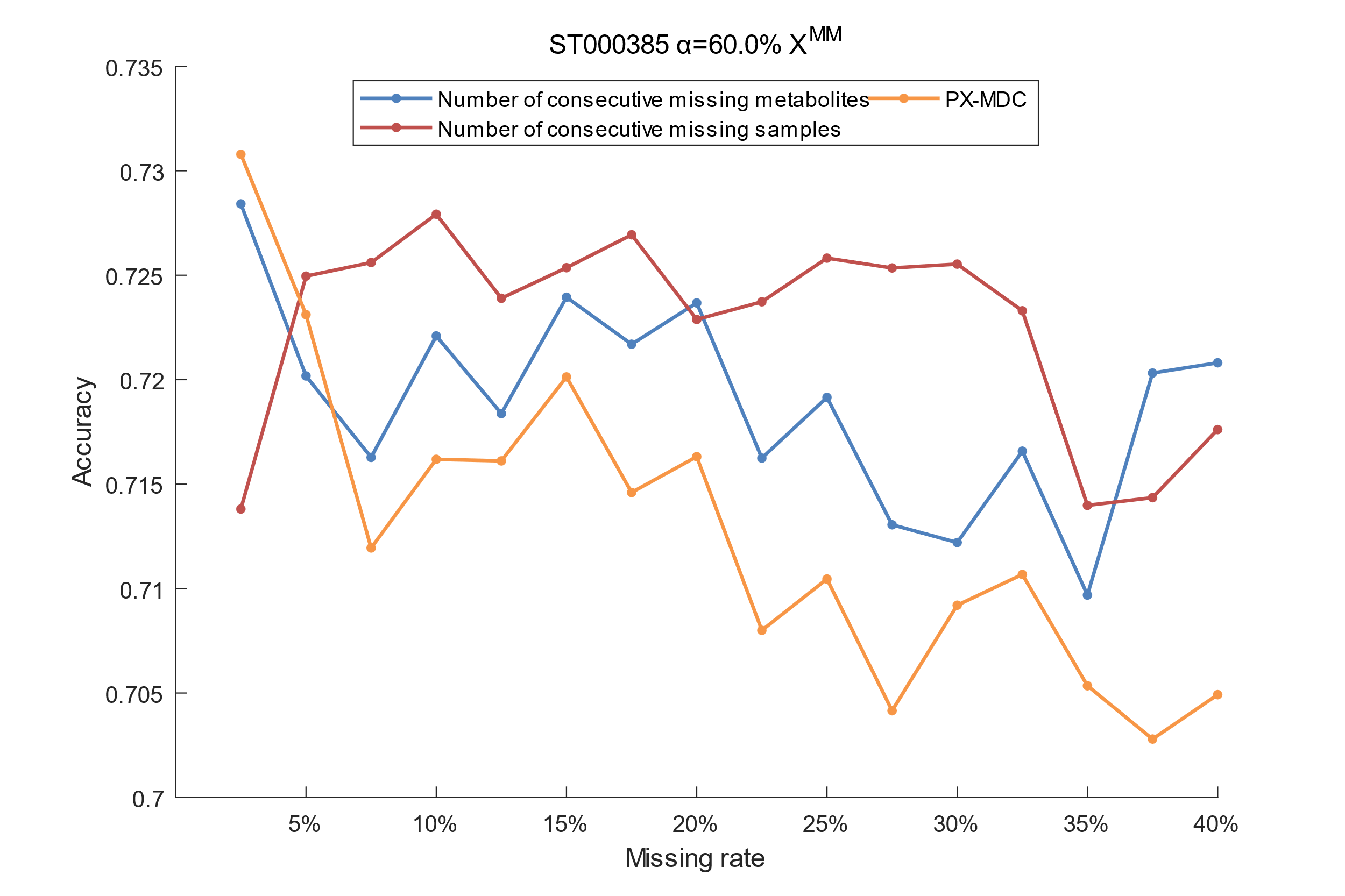 | 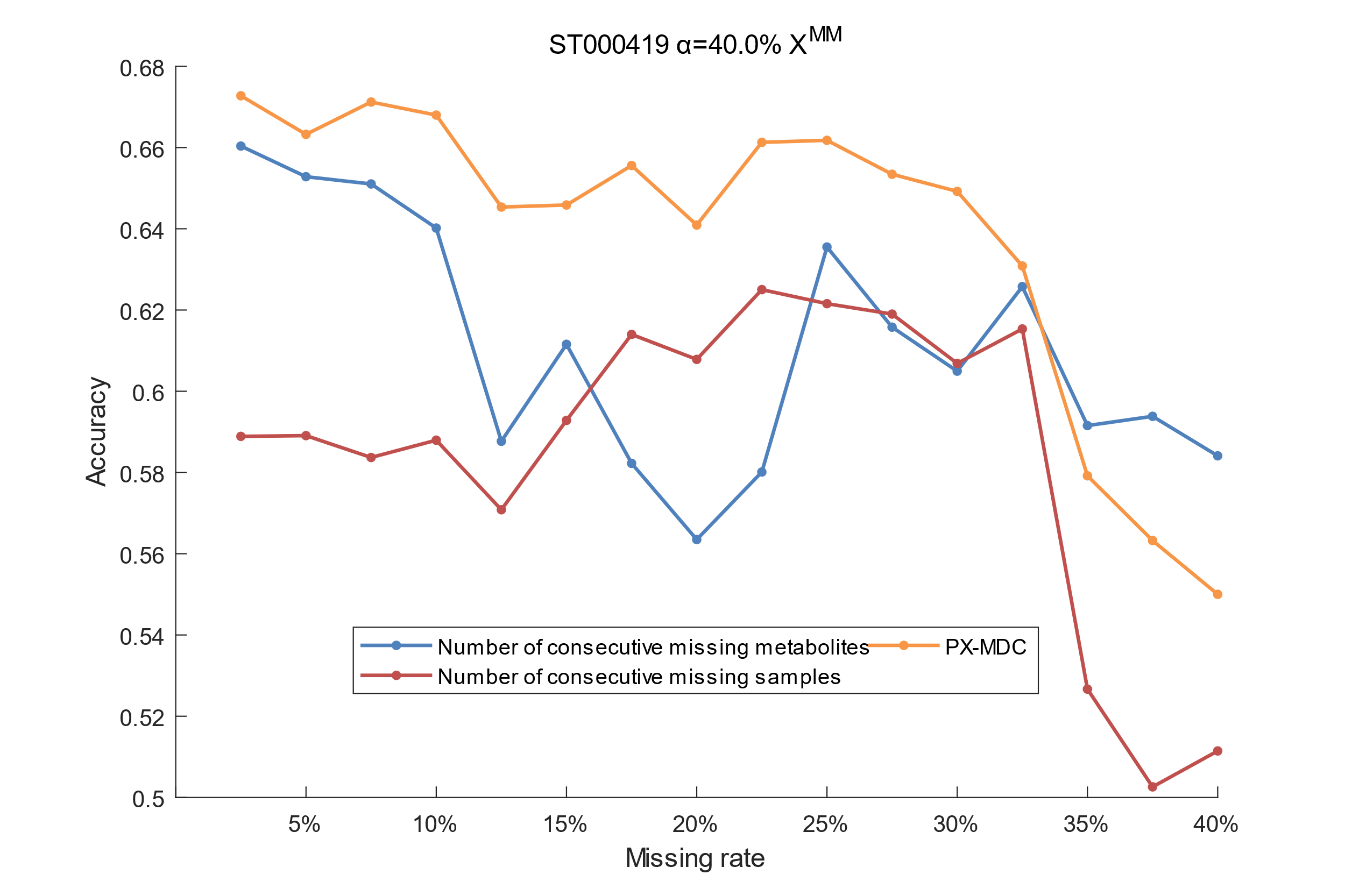 | 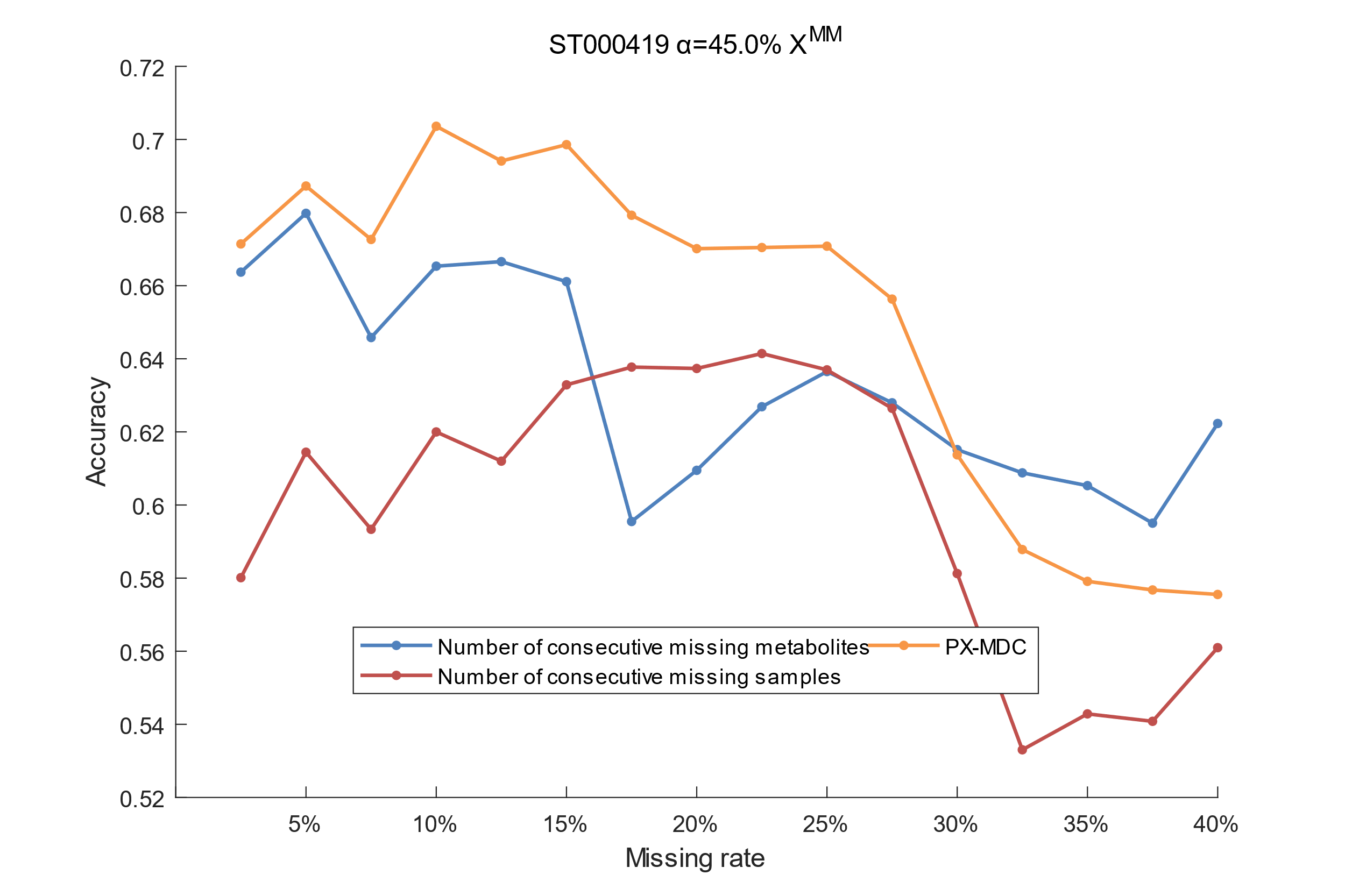 |
| 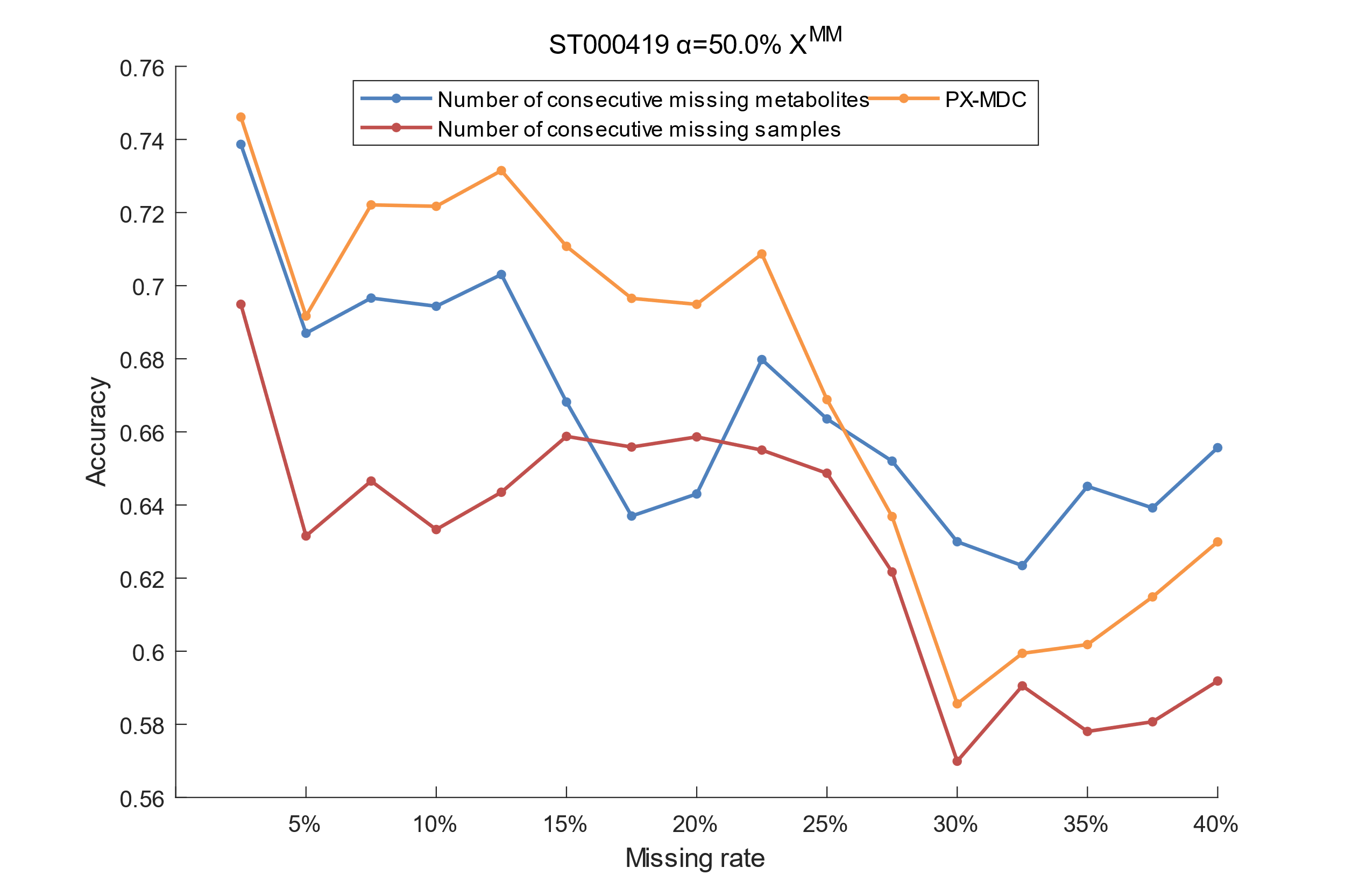 | 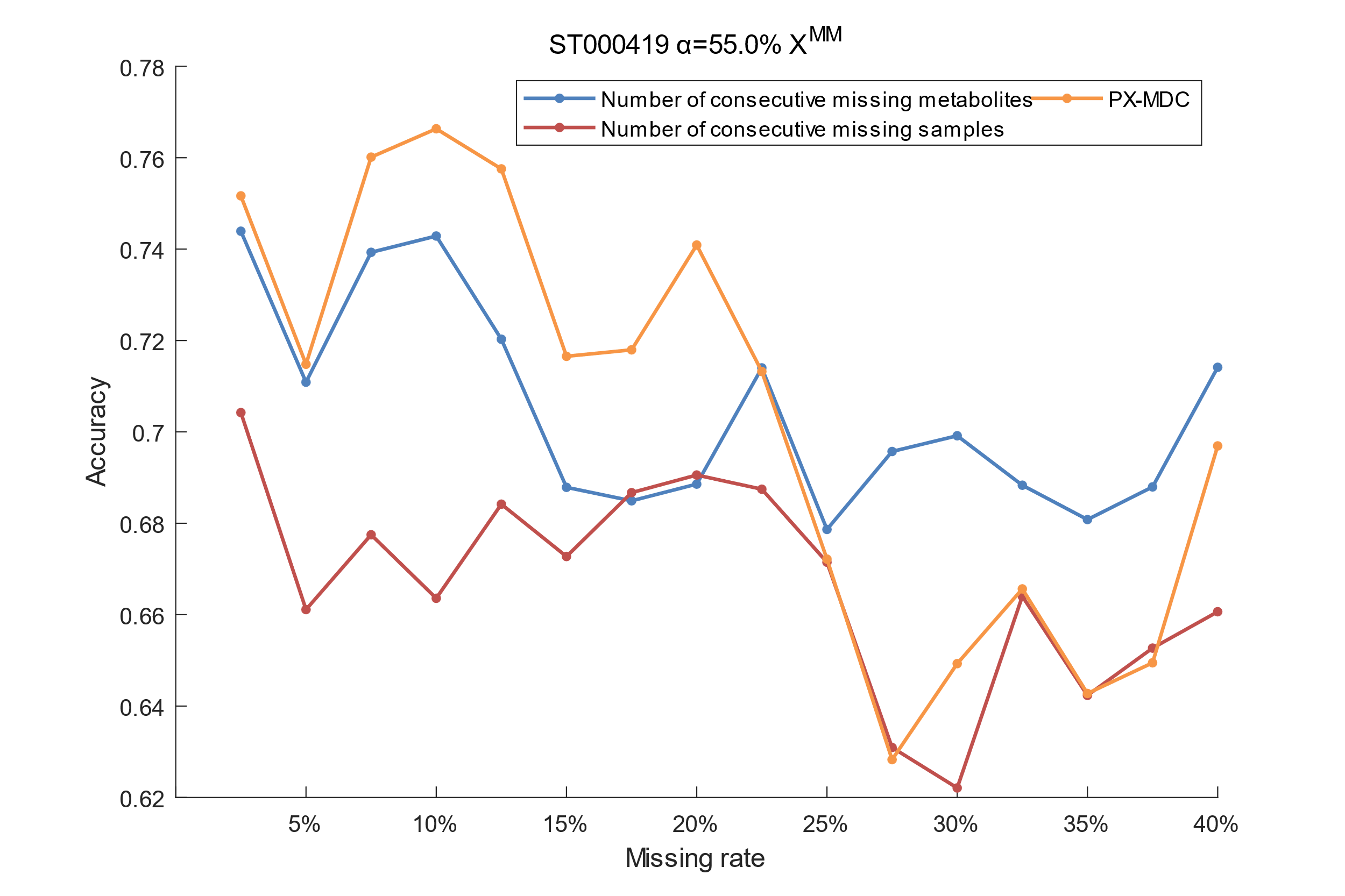 | 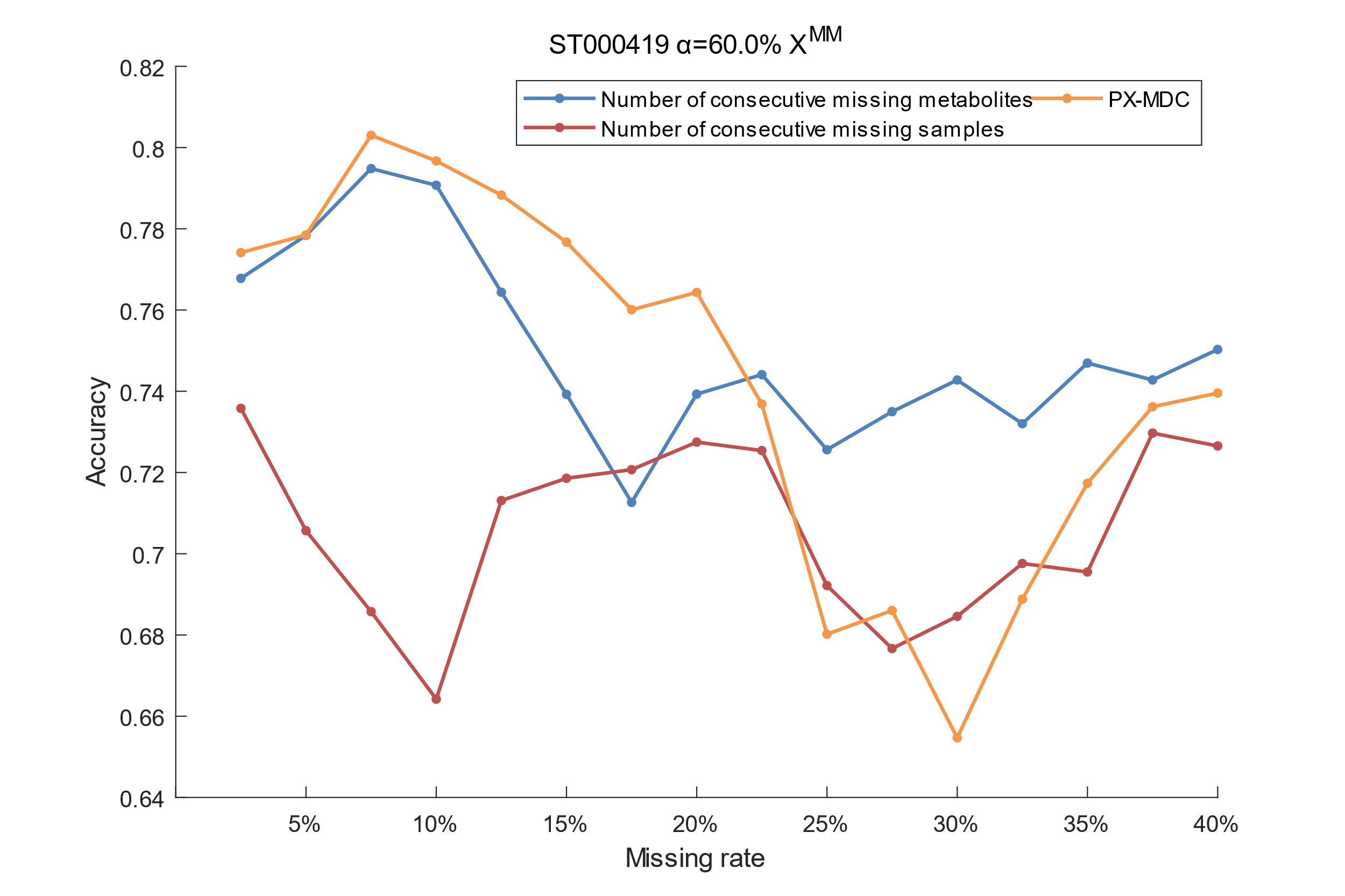 |

**Supplementary Figure 4.** Accuracy of X^MM^ in RF model for different features.

| 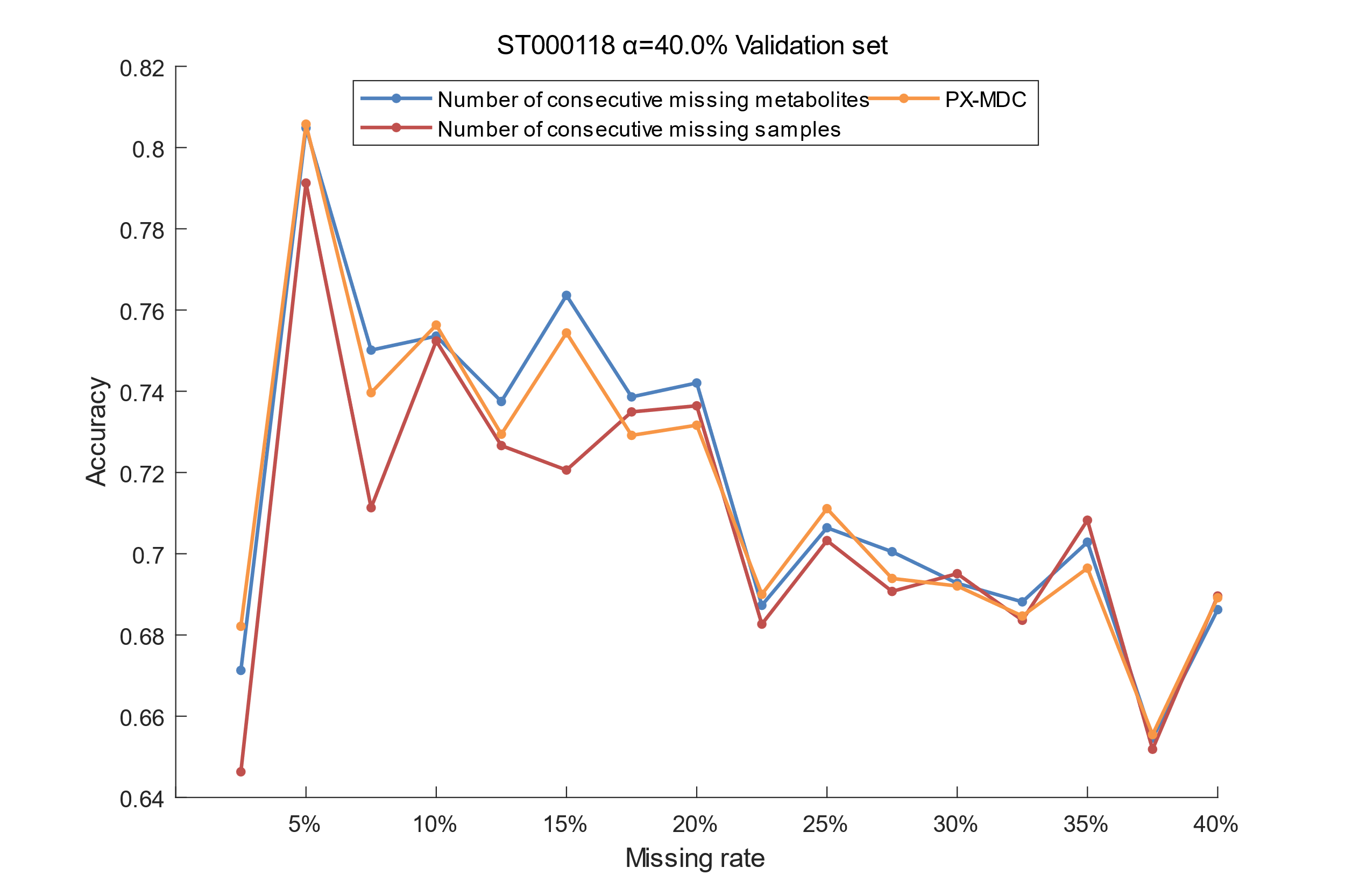 | 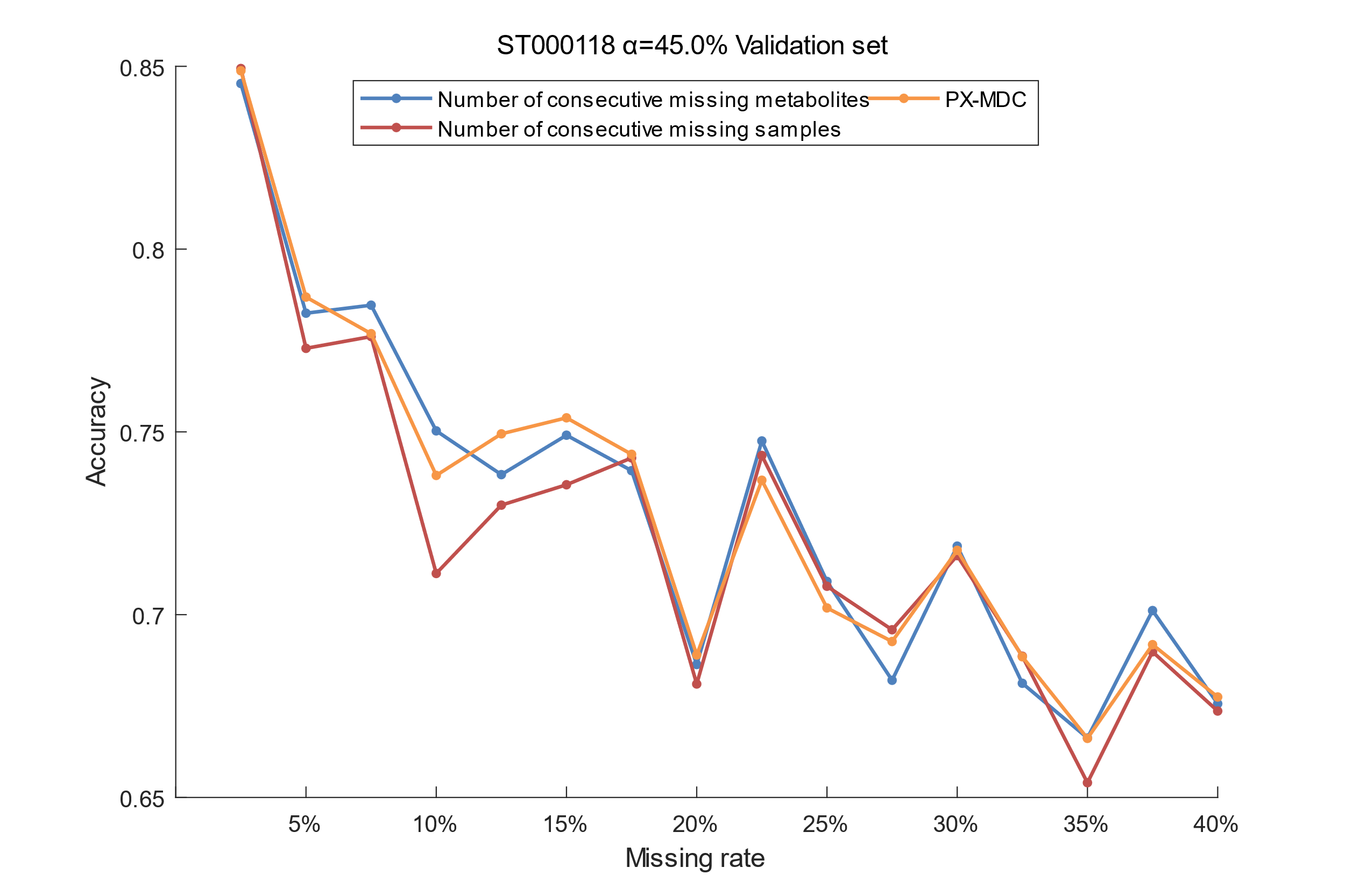 | 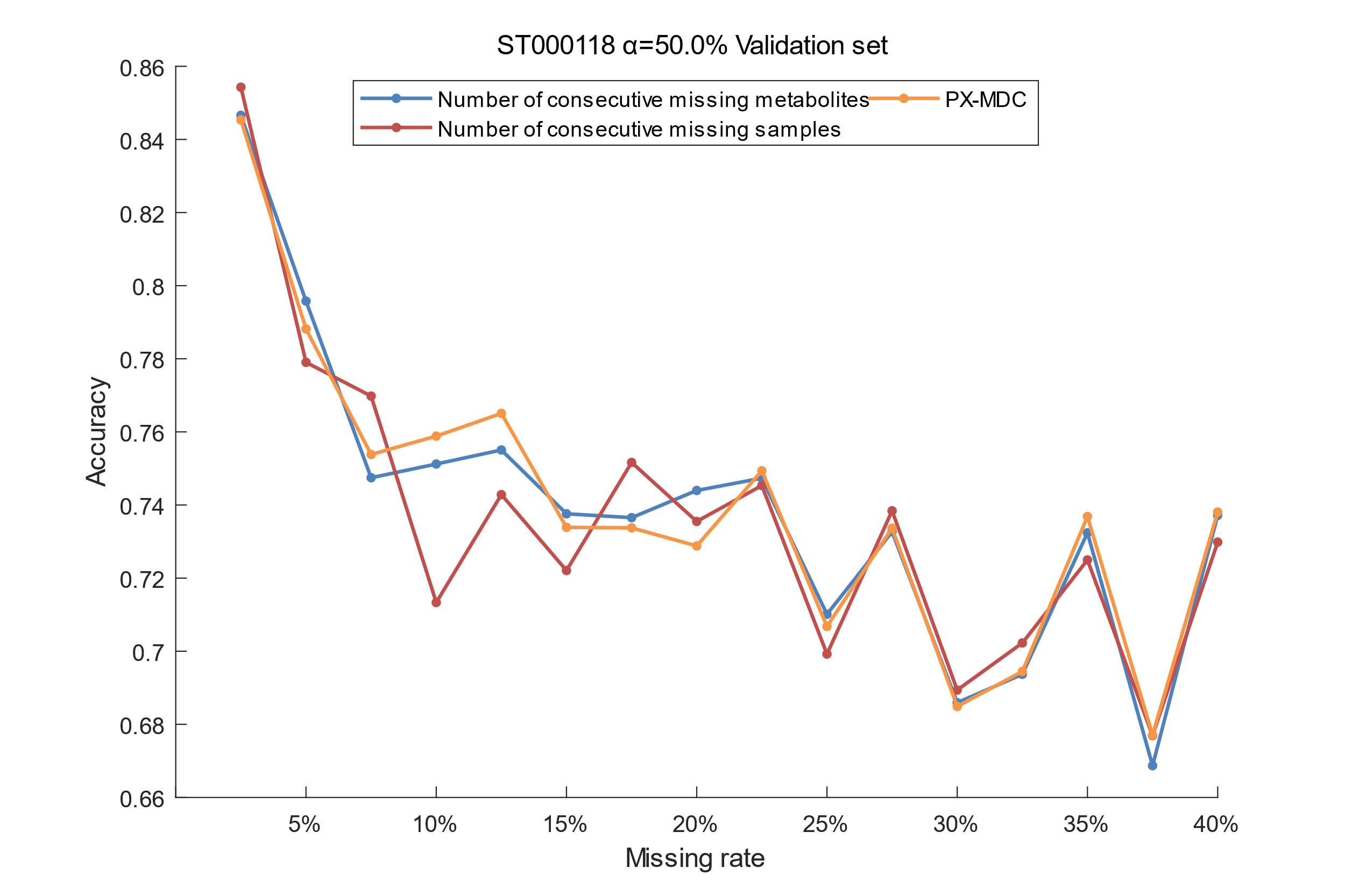 |
| --- | --- | --- |
| 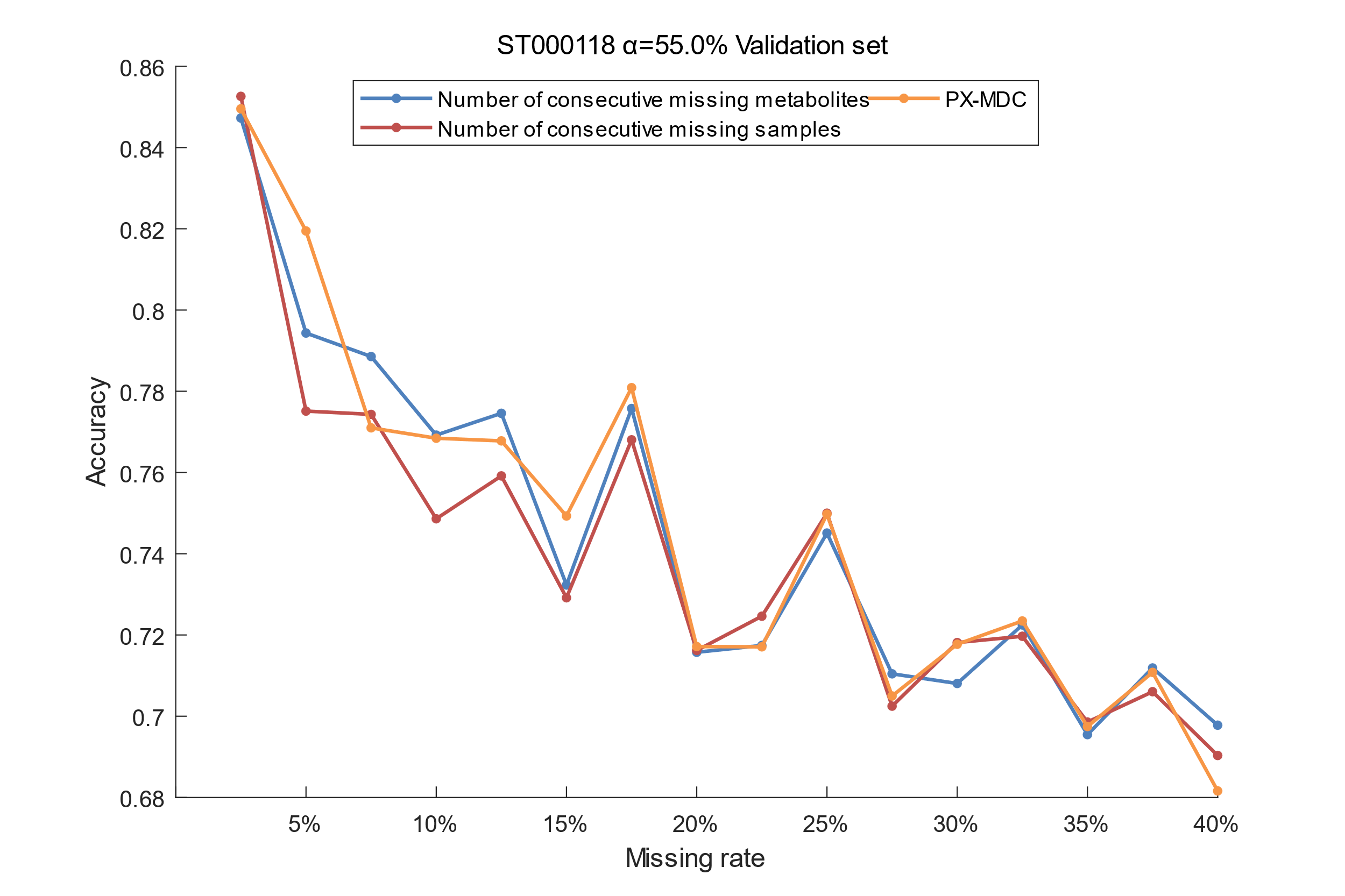 | 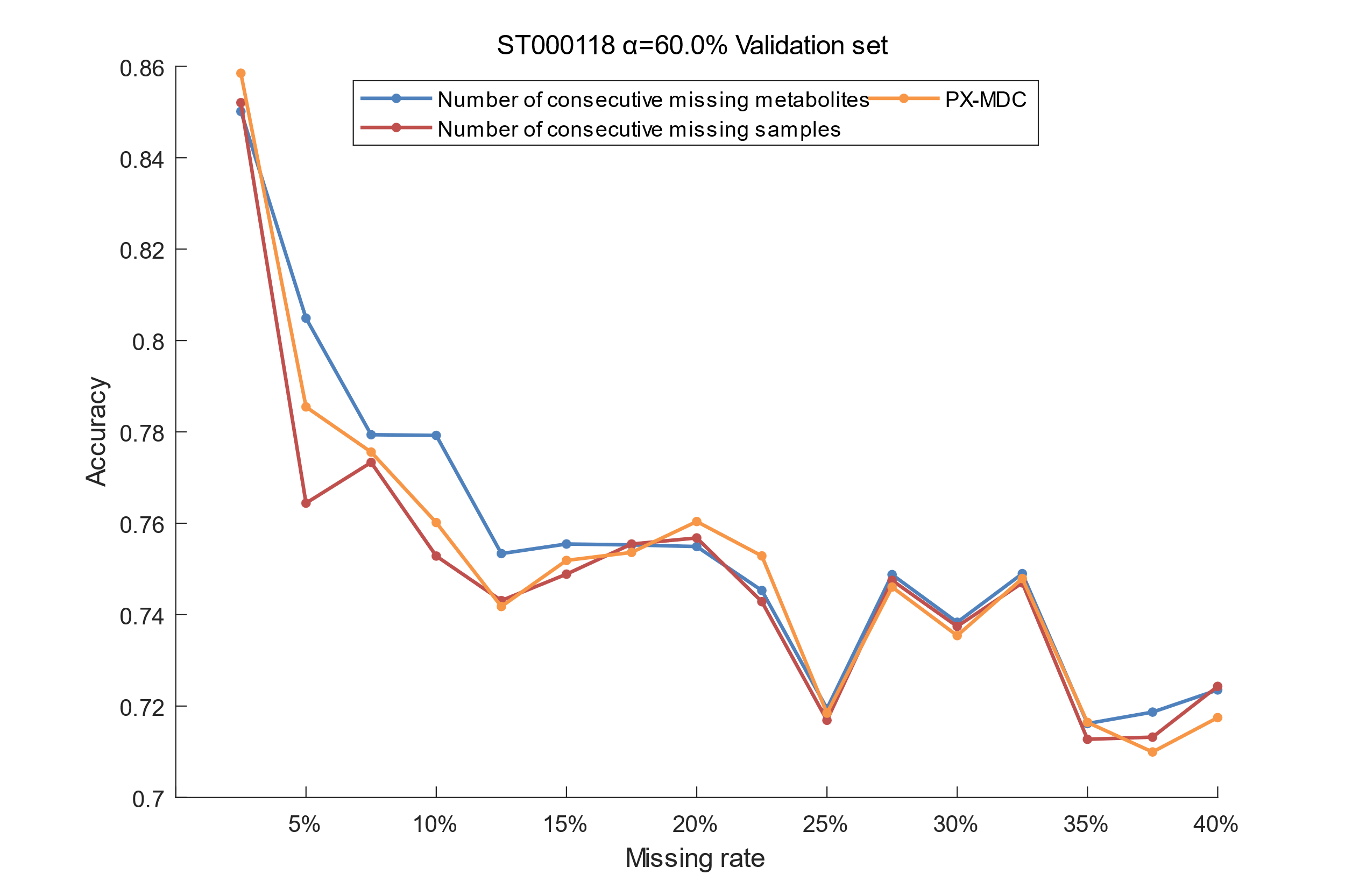 | 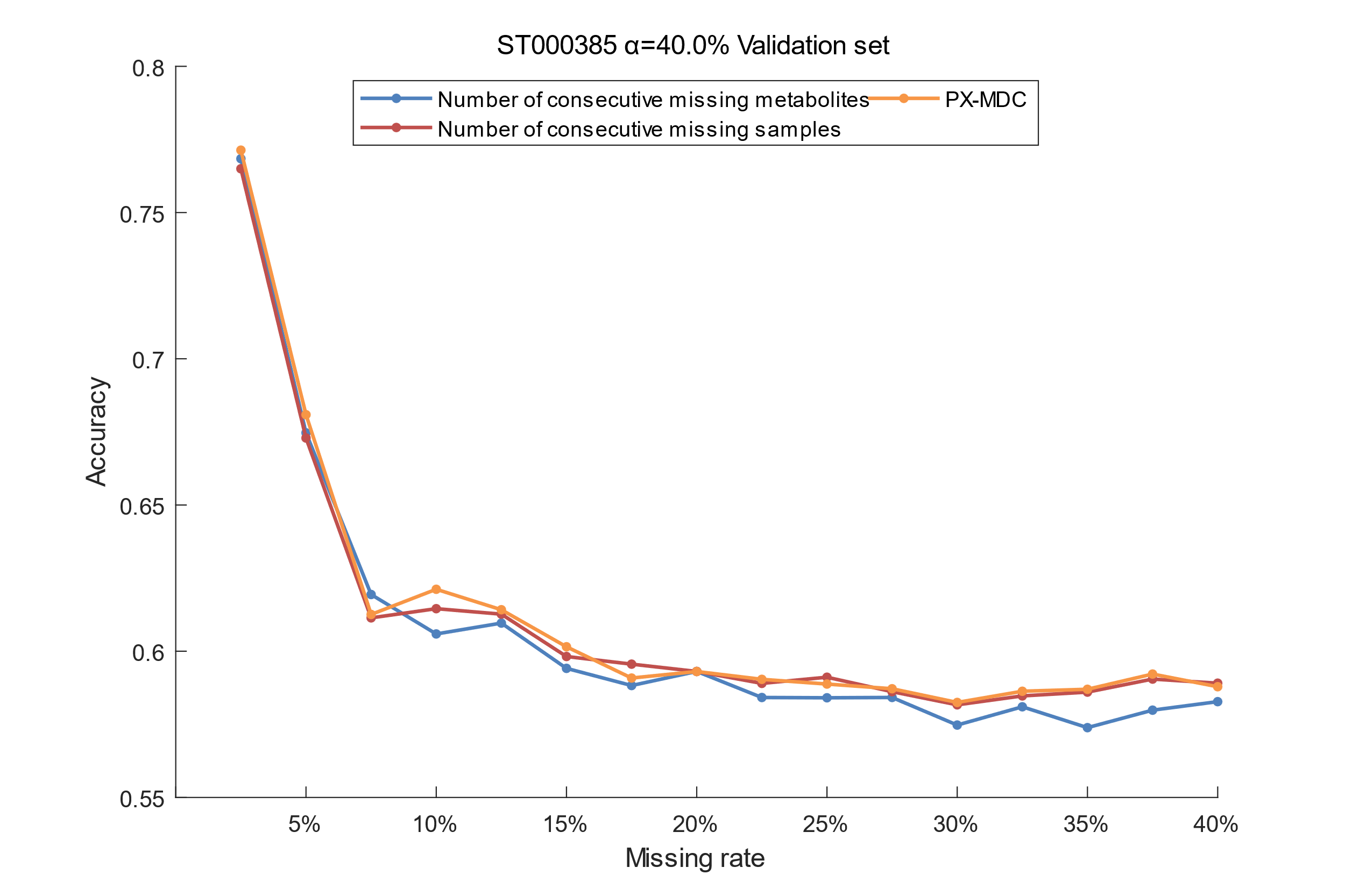 |
| 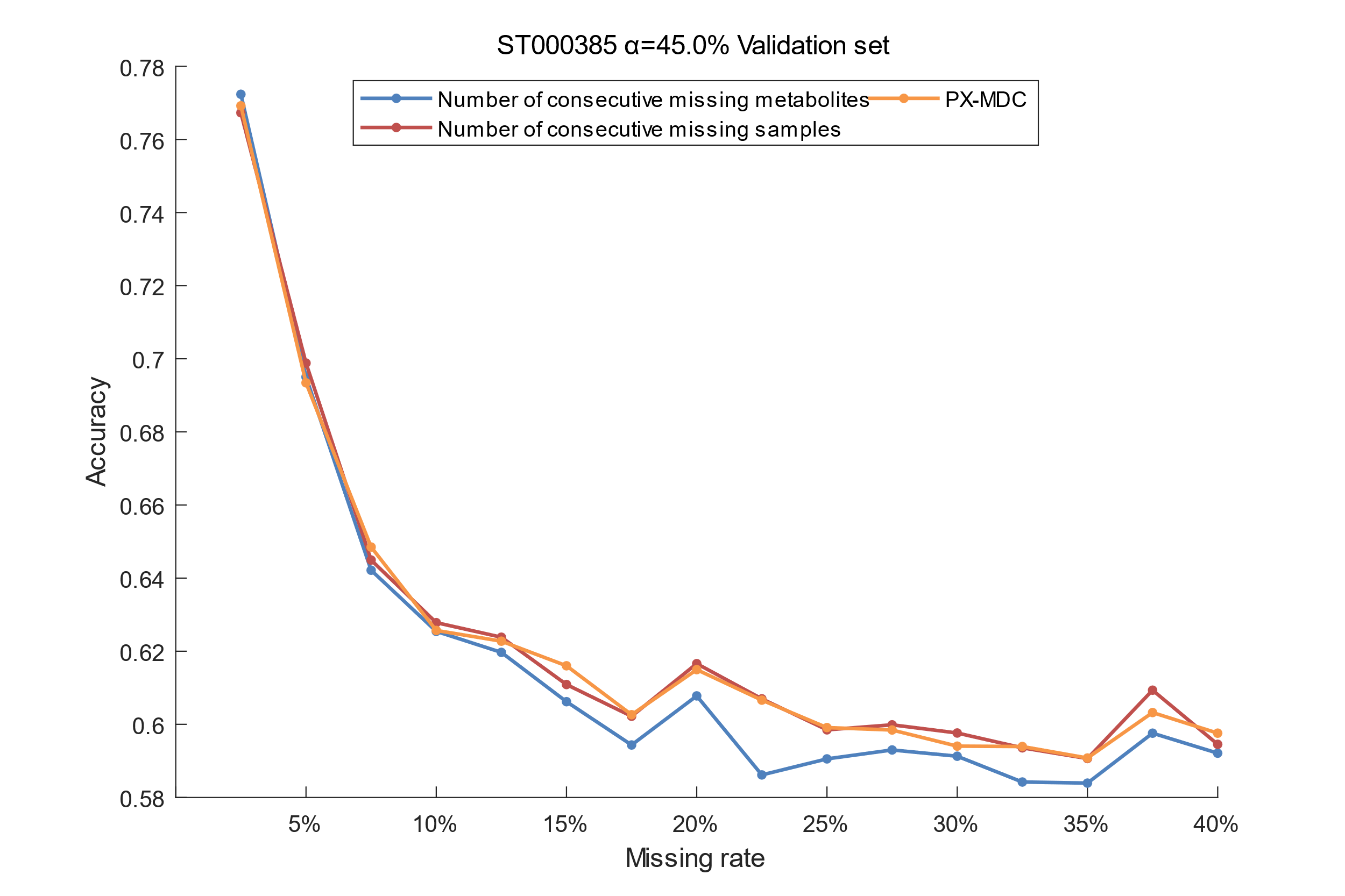 | 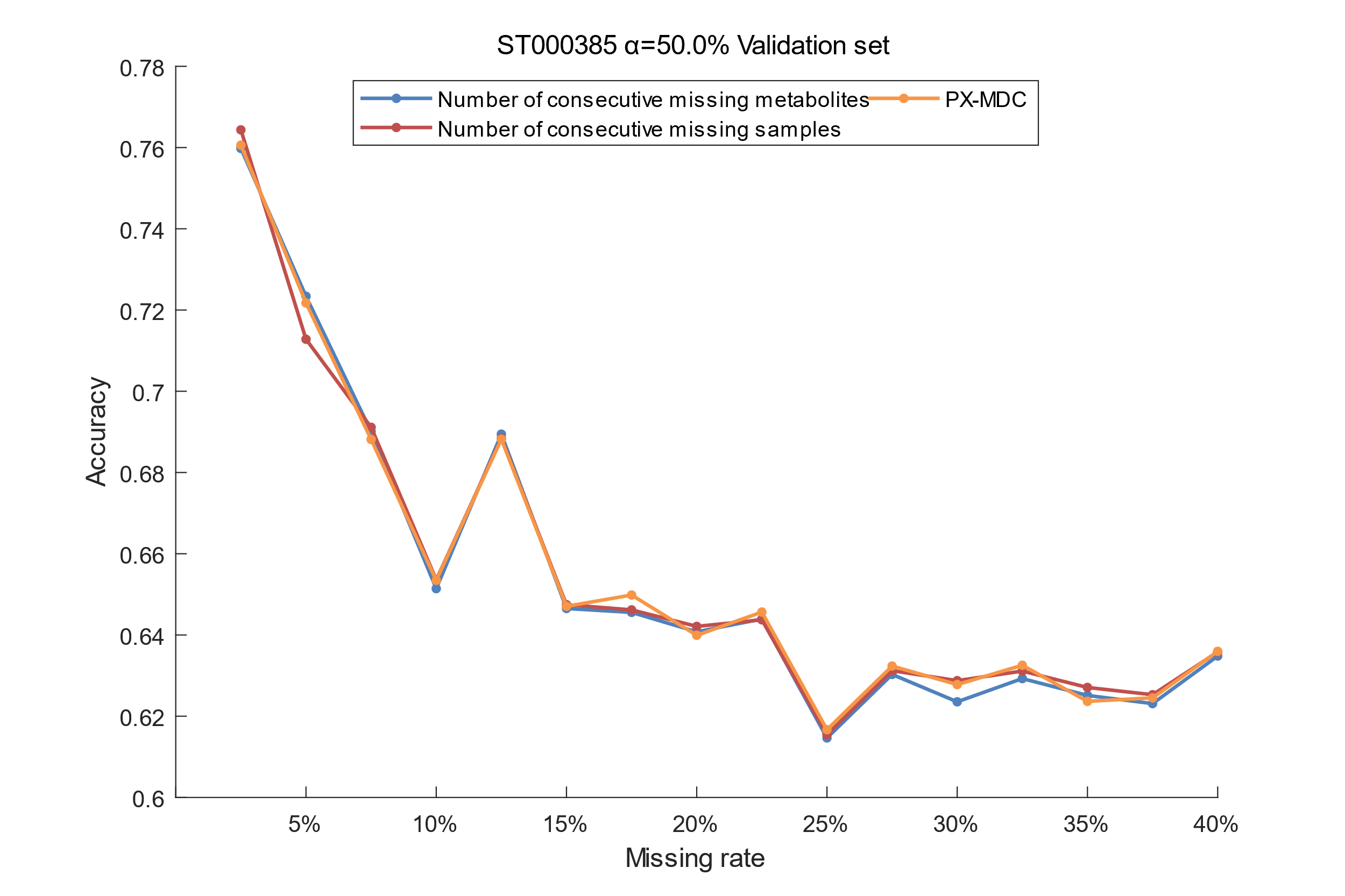 | 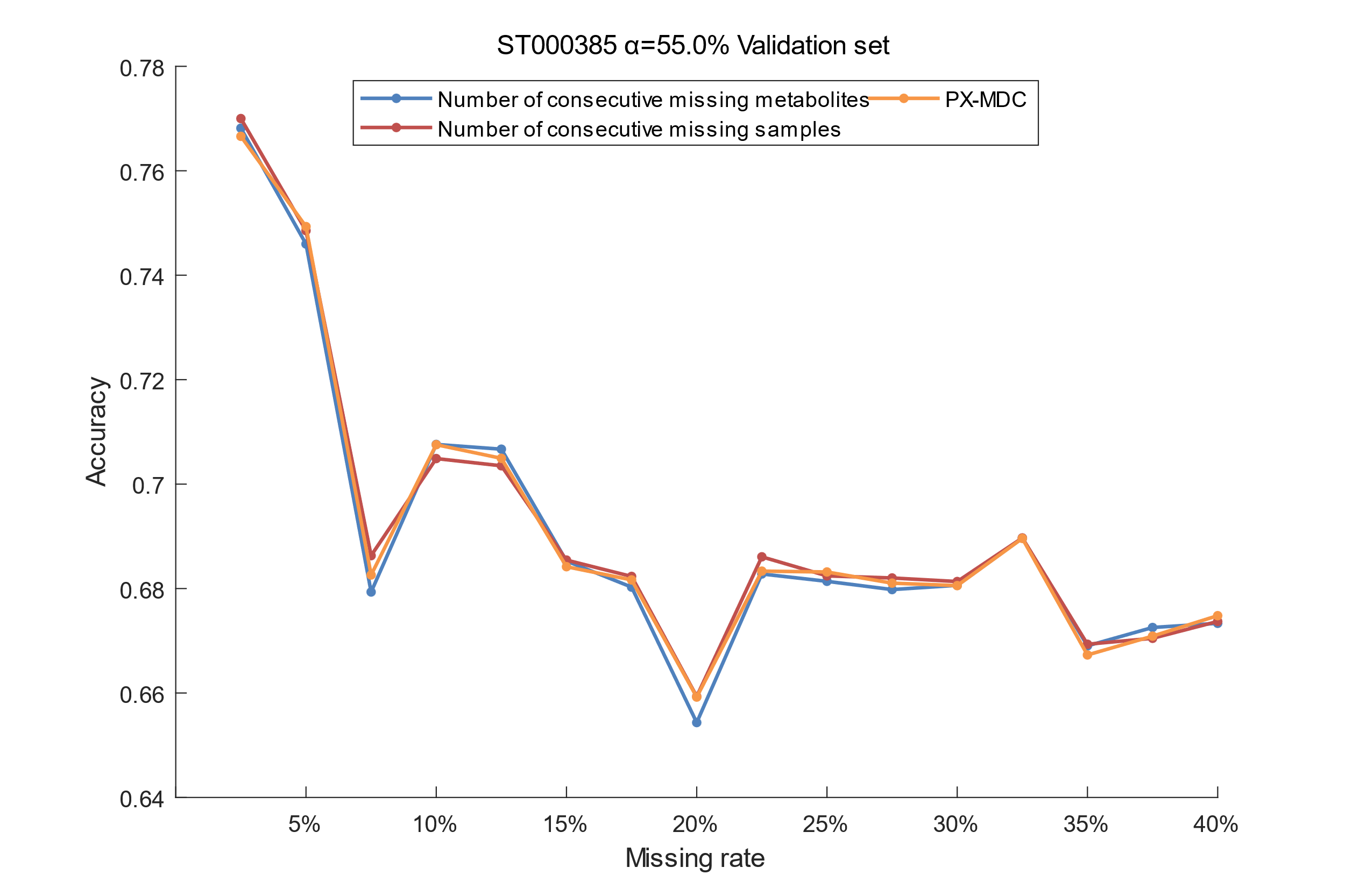 |
| 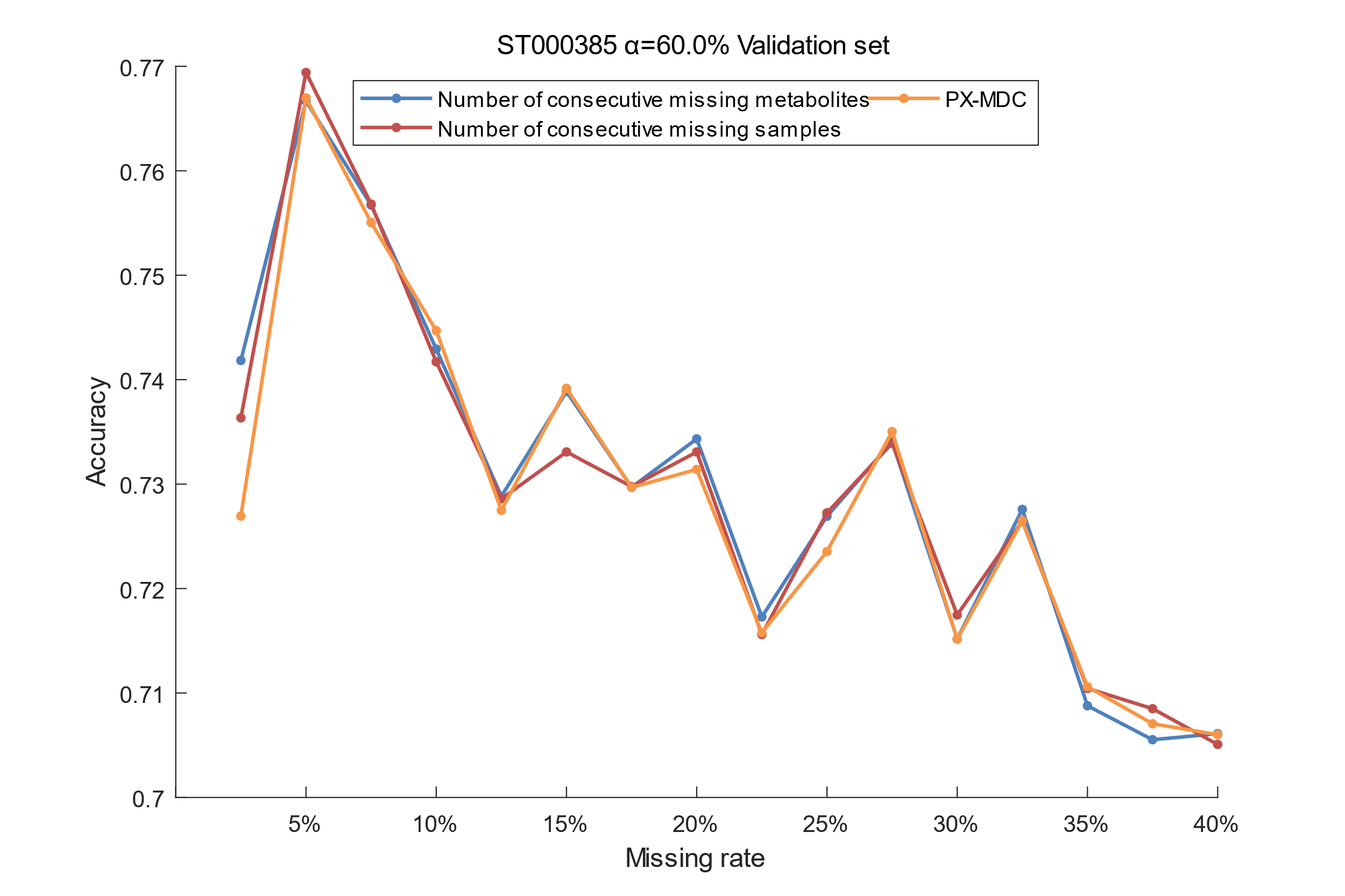 | 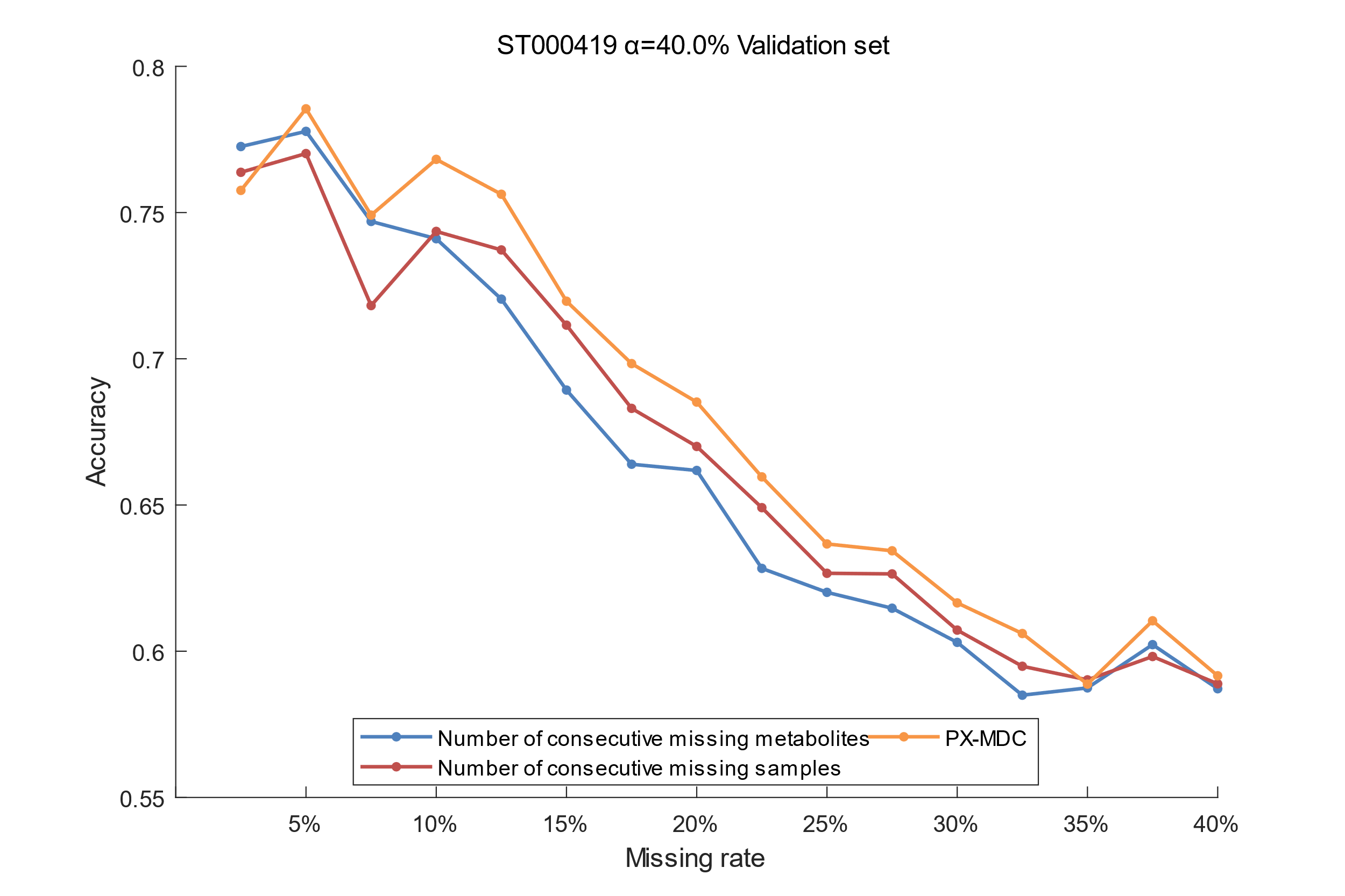 | 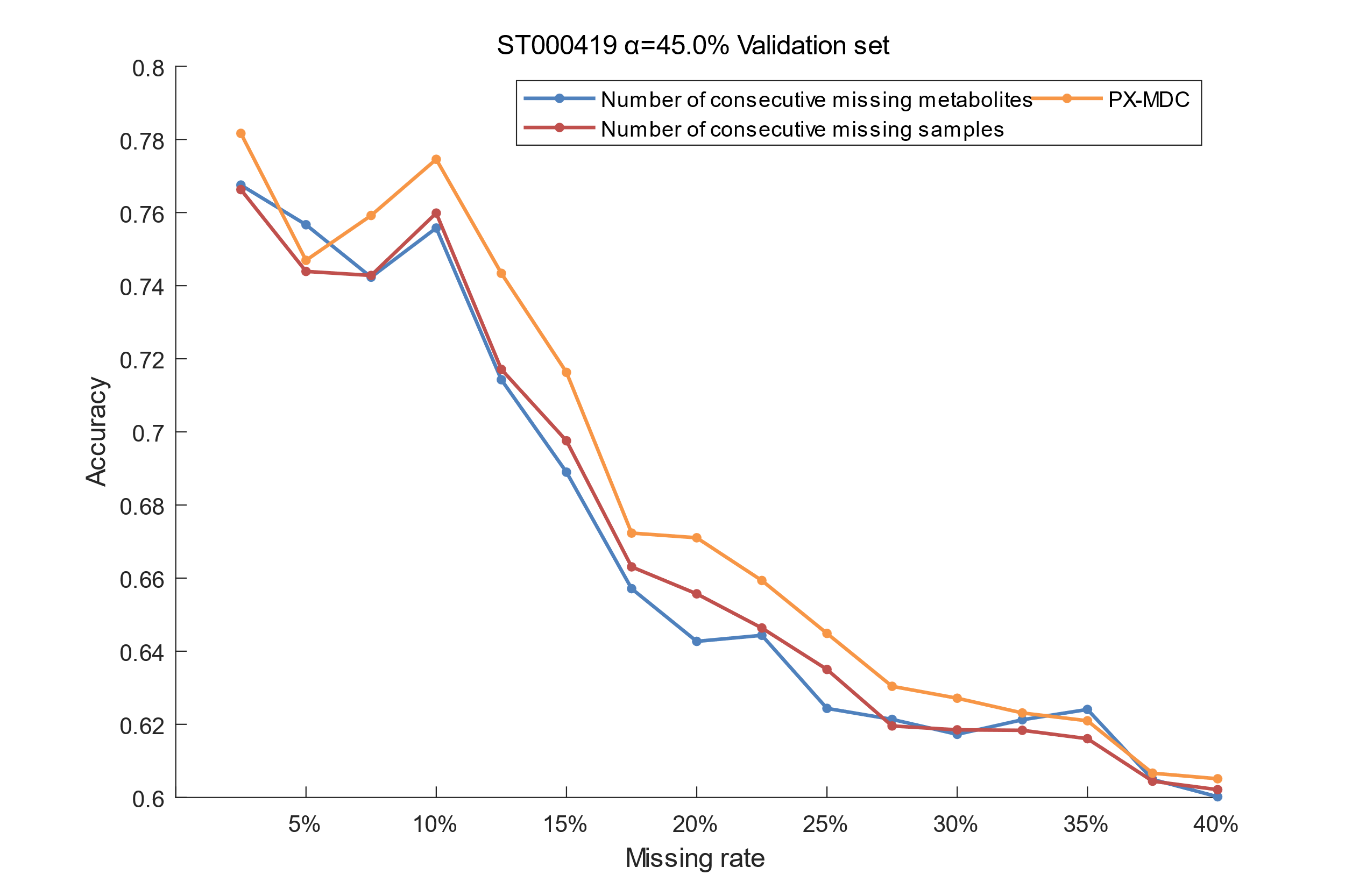 |
| 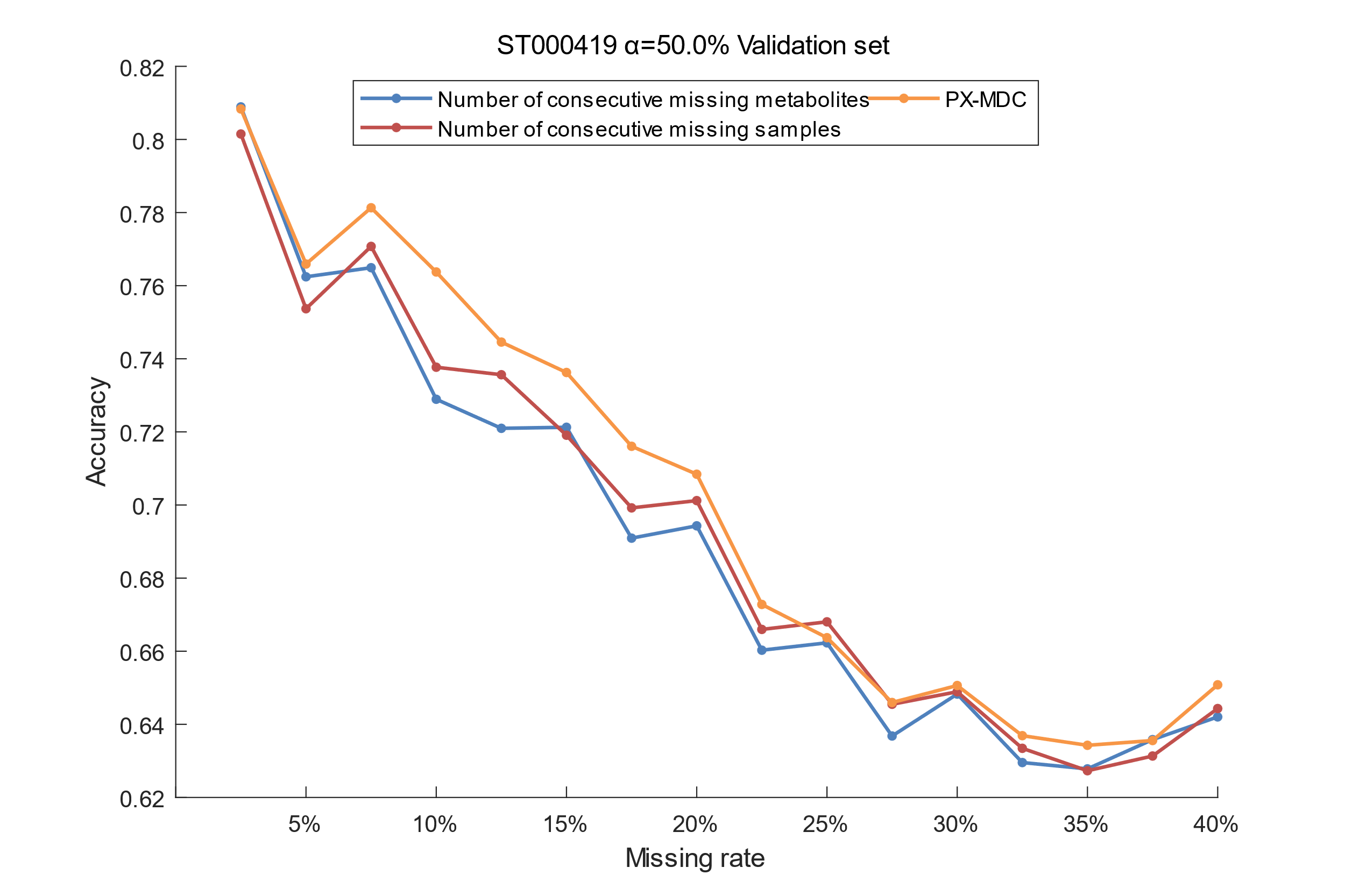 | 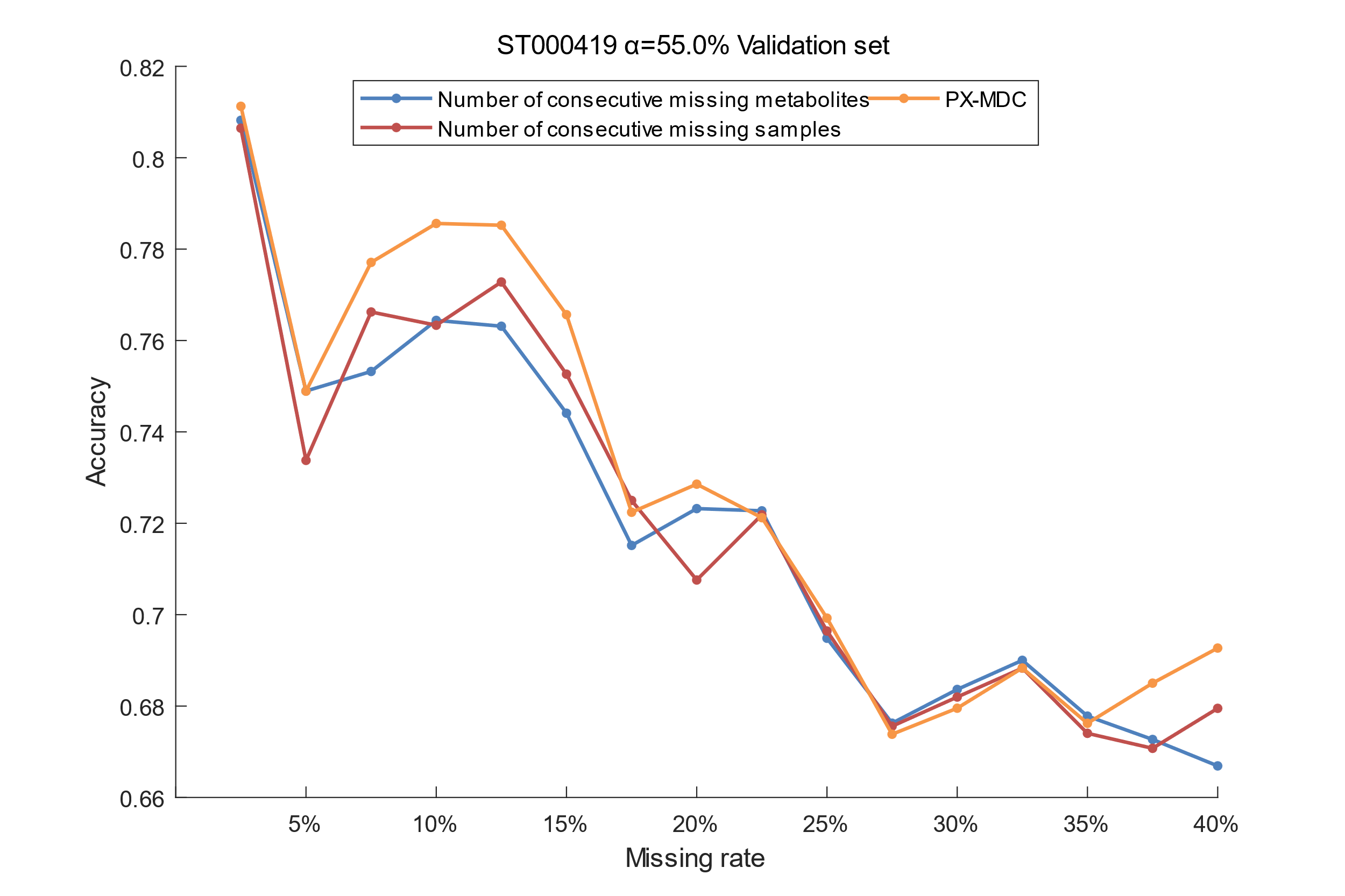 | 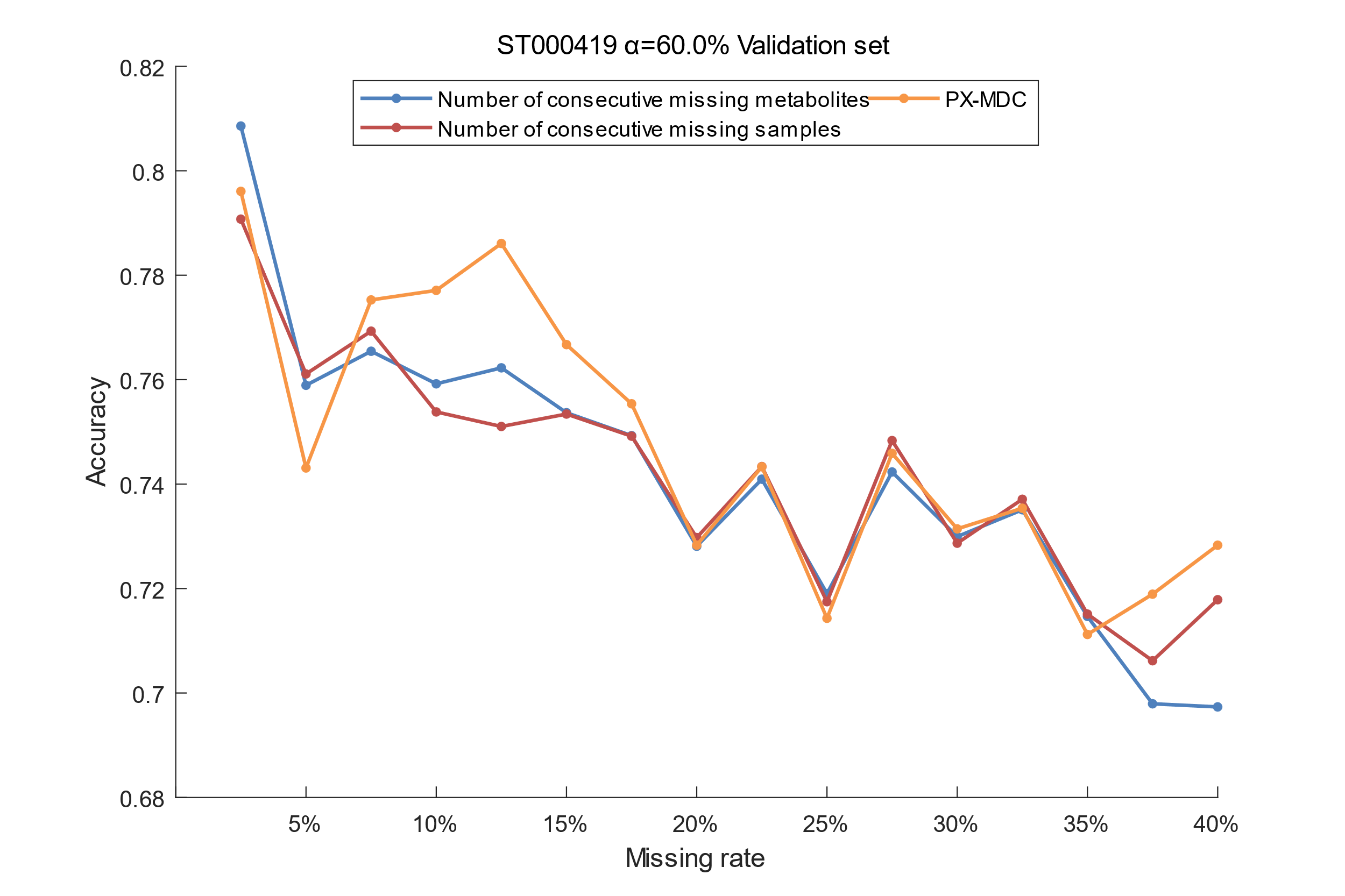 |

**Supplementary Figure 5.** Accuracy of validation sets in BP Neural Network models for different feature.

| 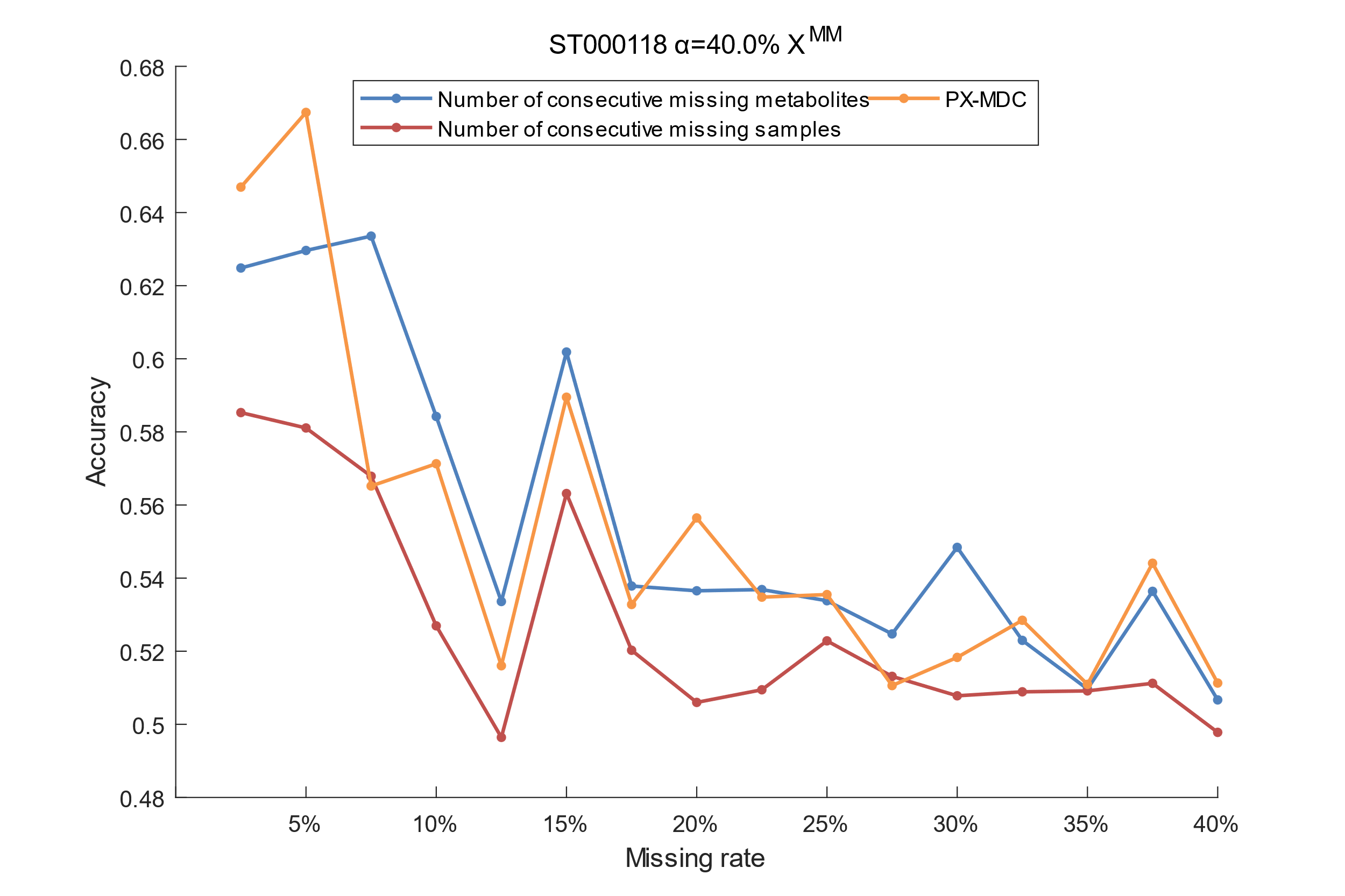 | 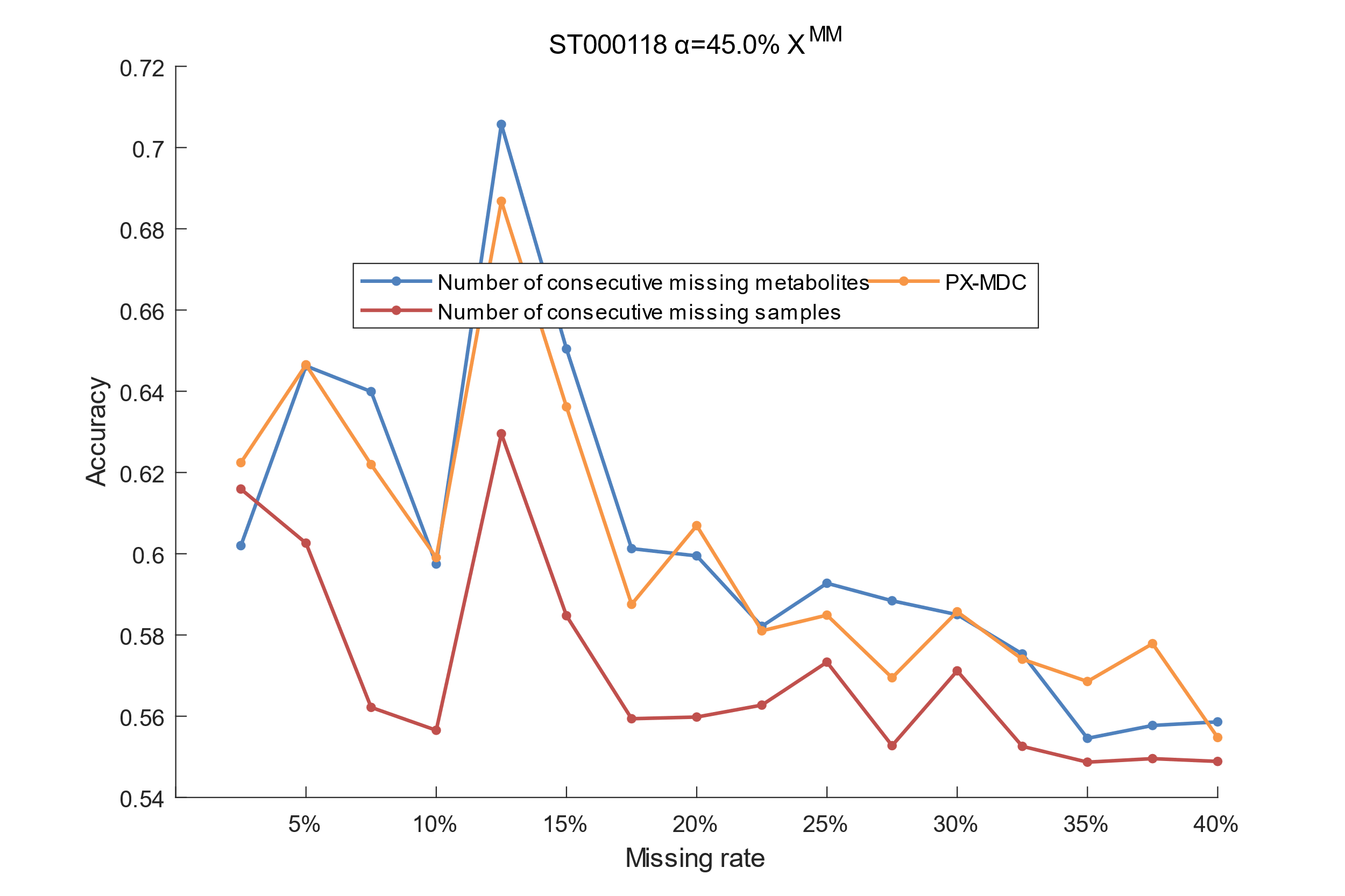 | 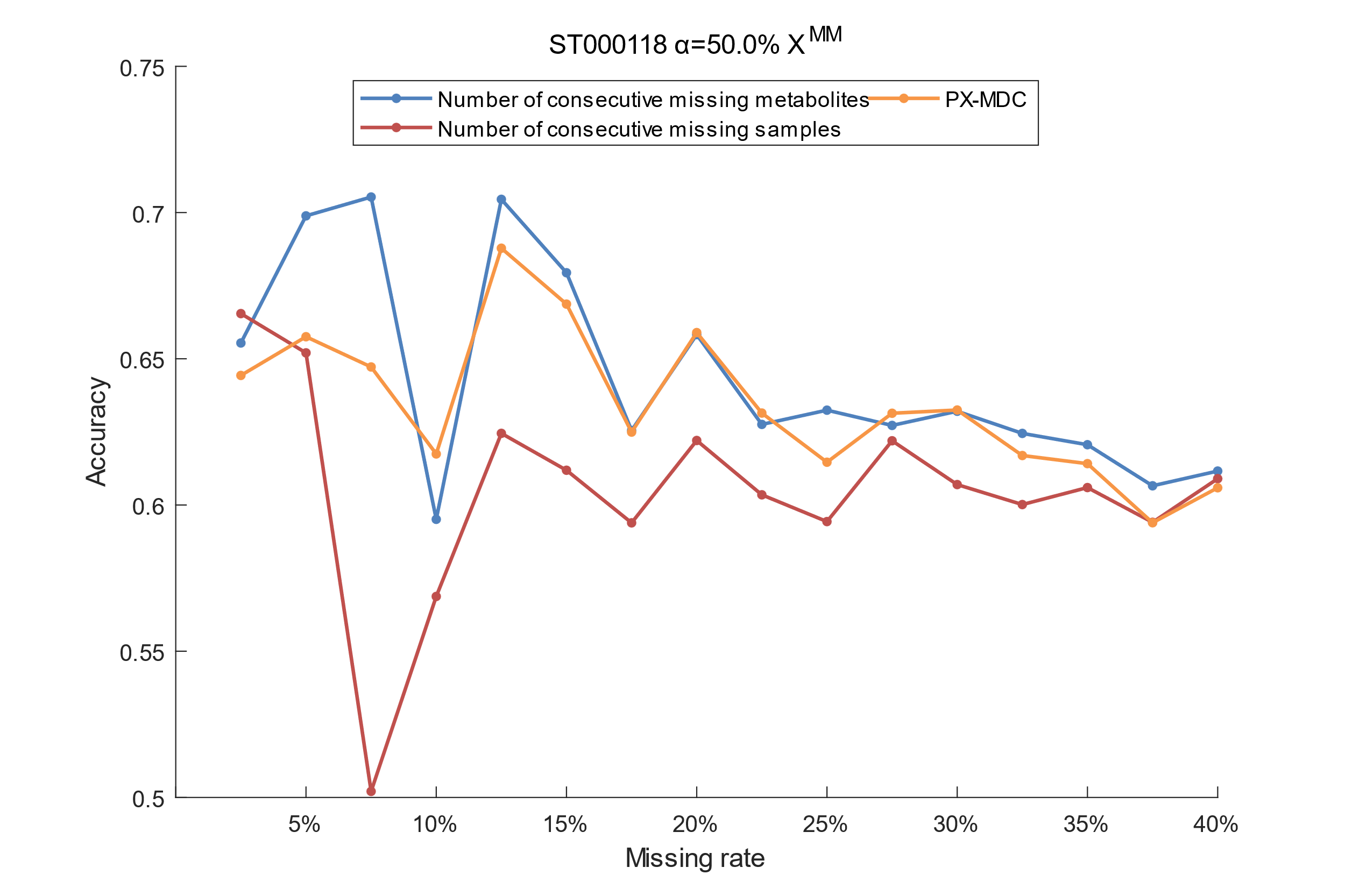 |
| --- | --- | --- |
| 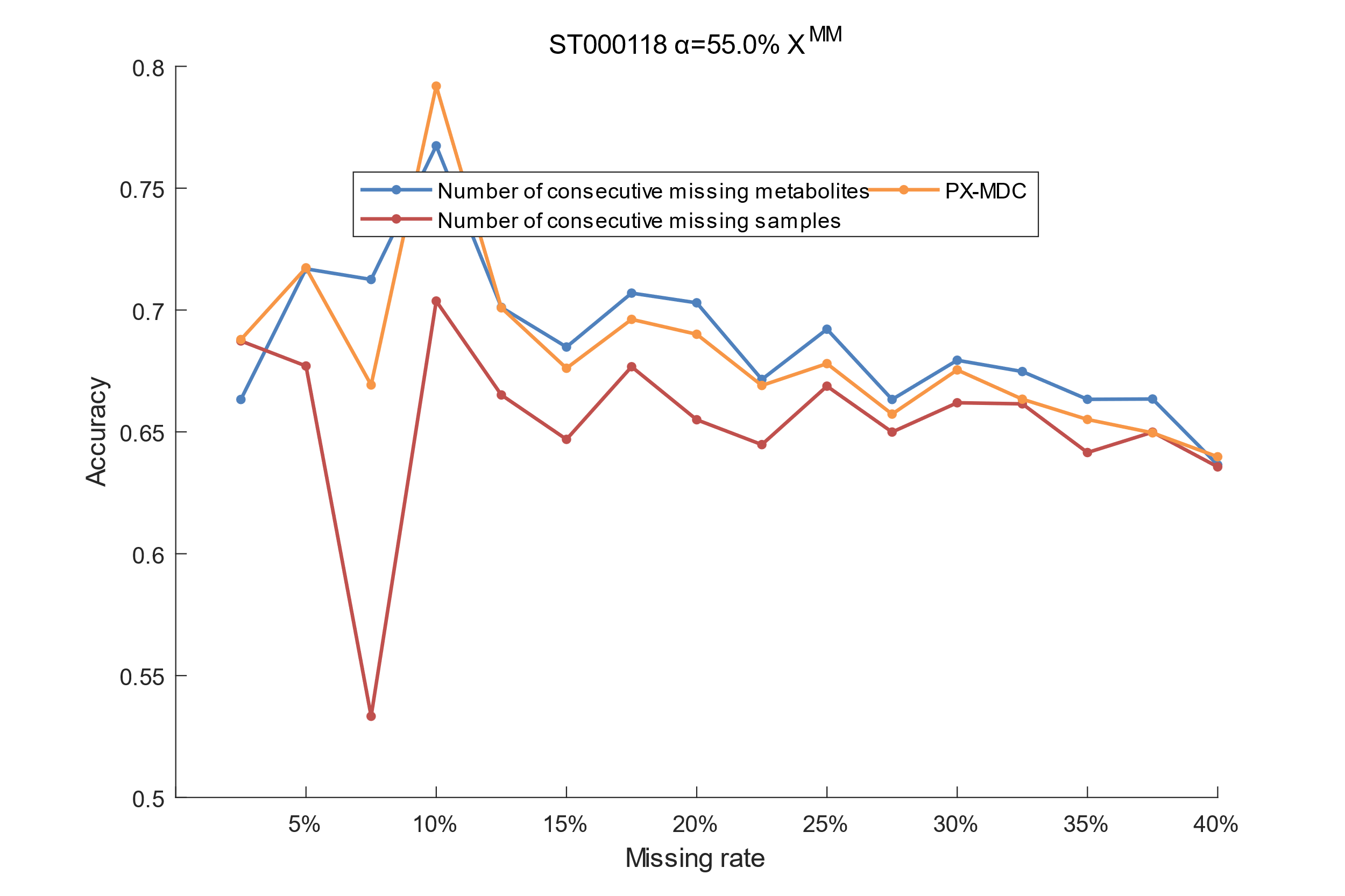 | 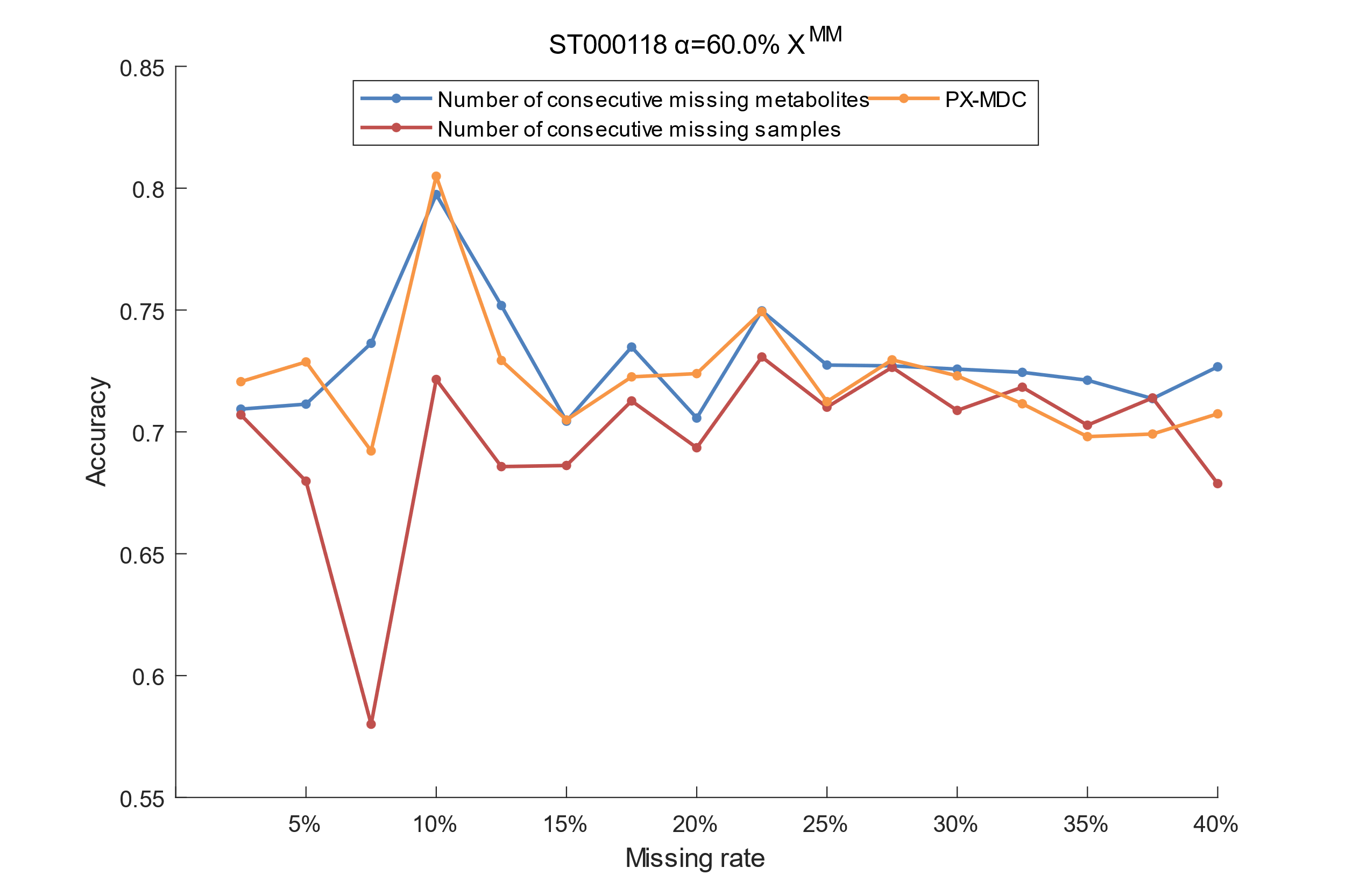 | 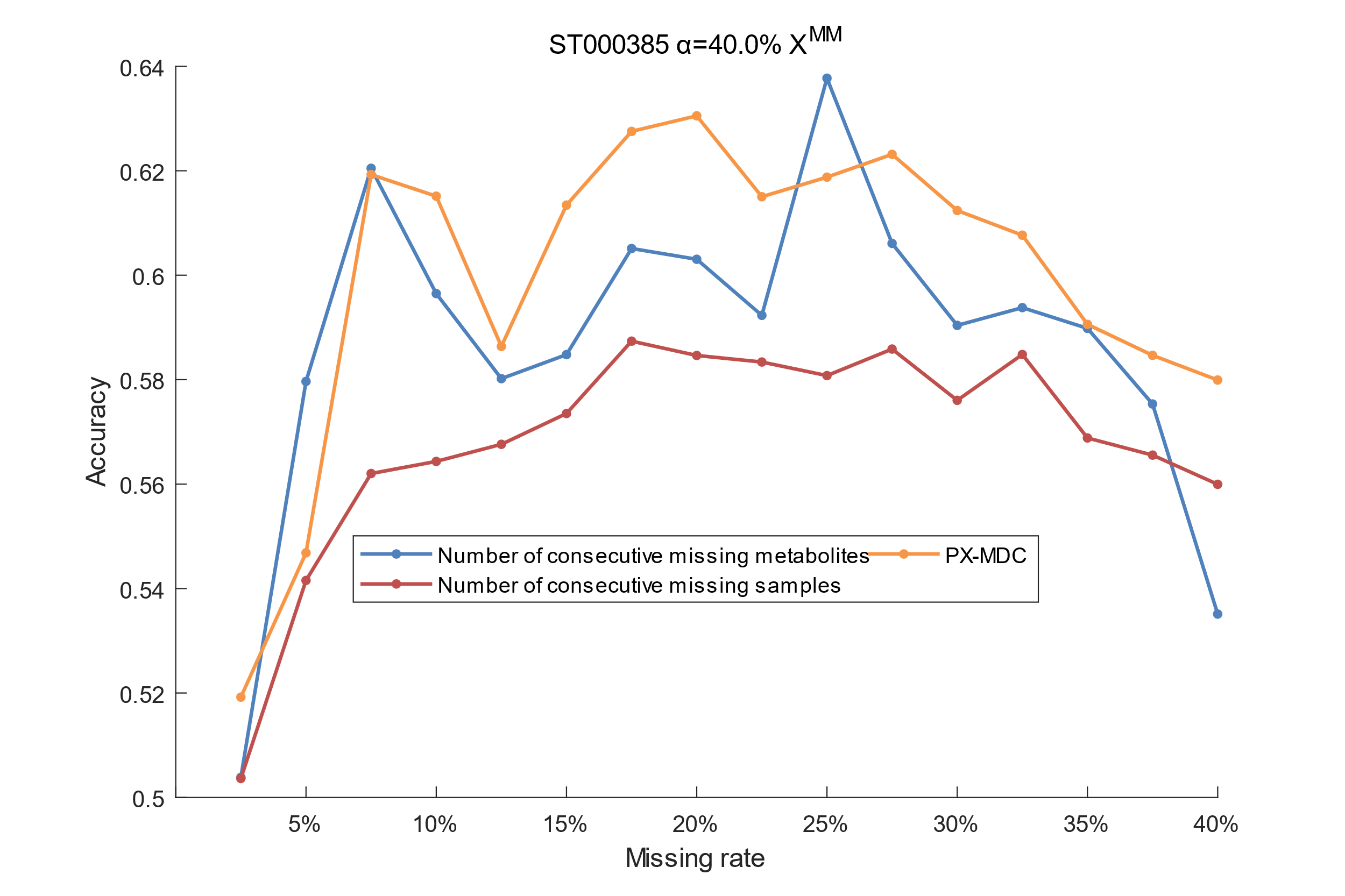 |
| 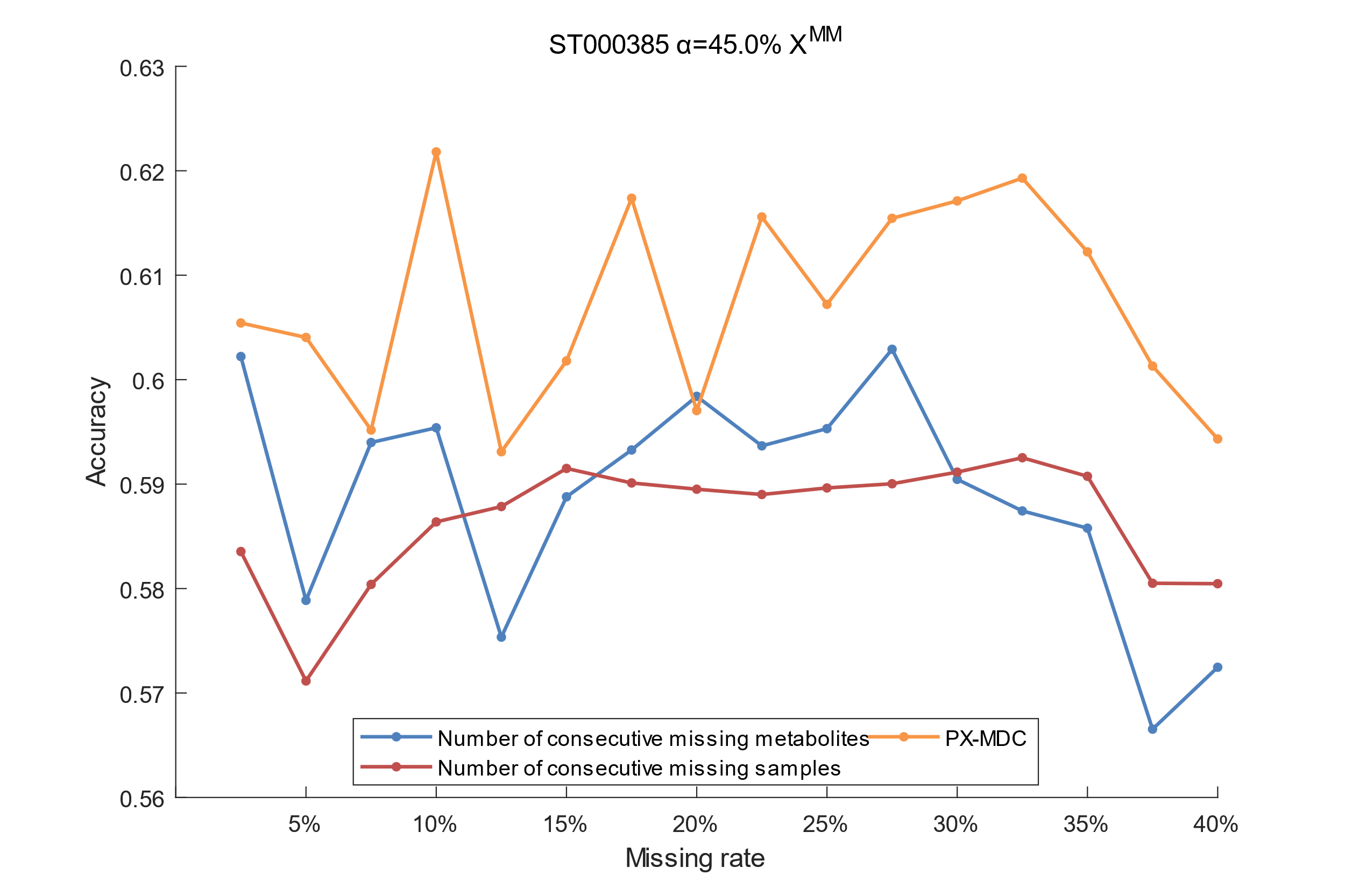 | 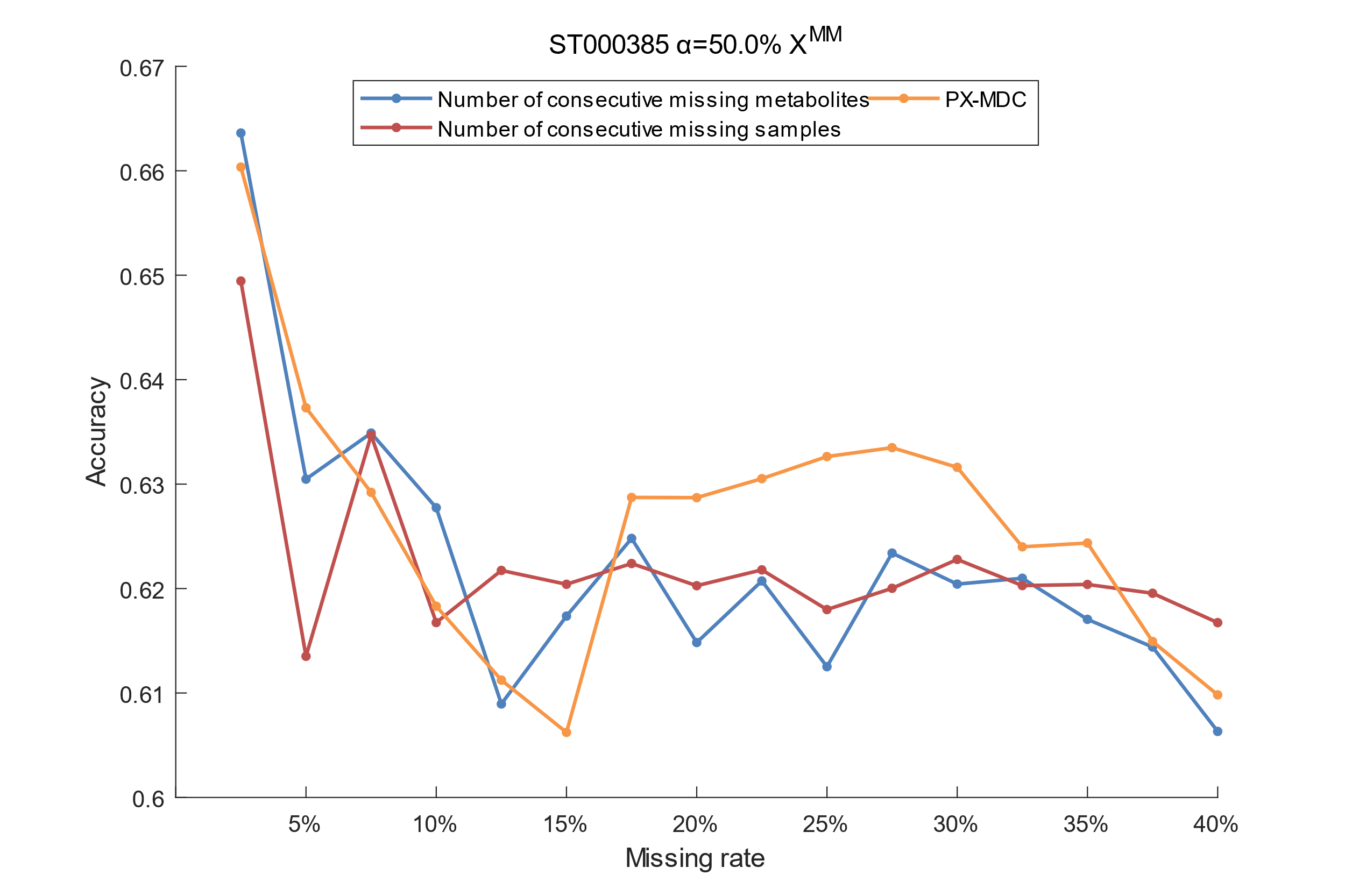 | 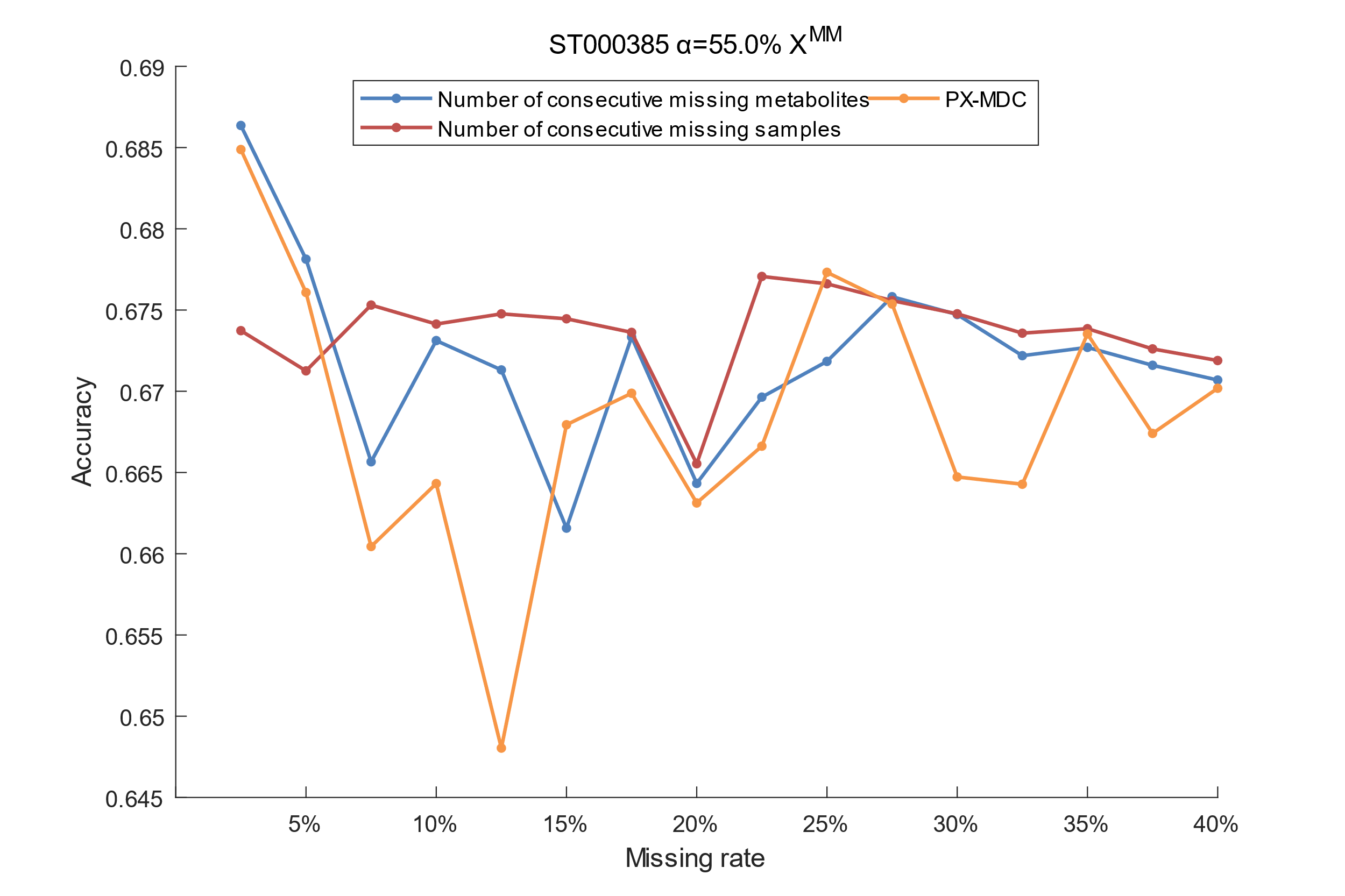 |
| 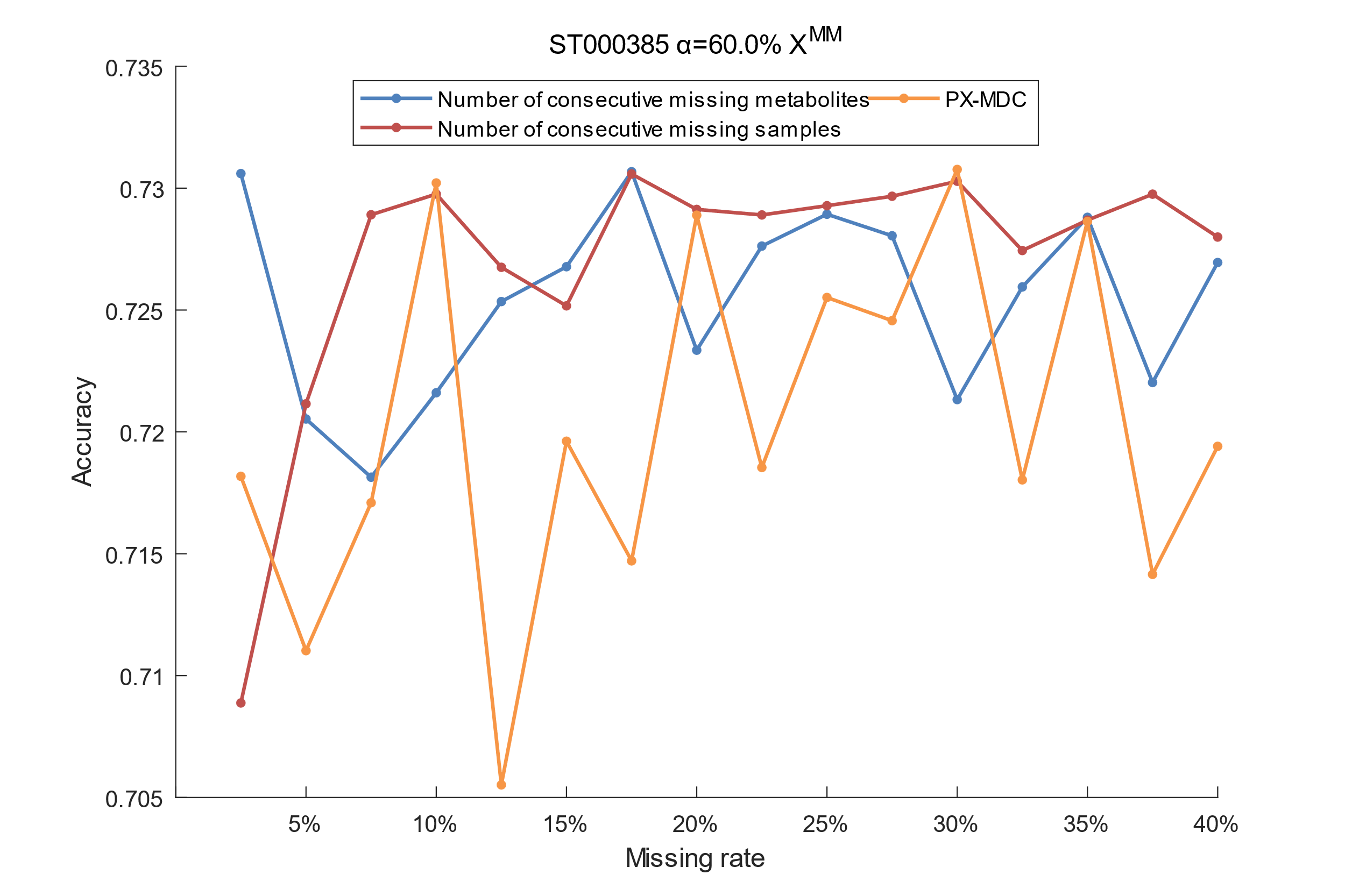 | 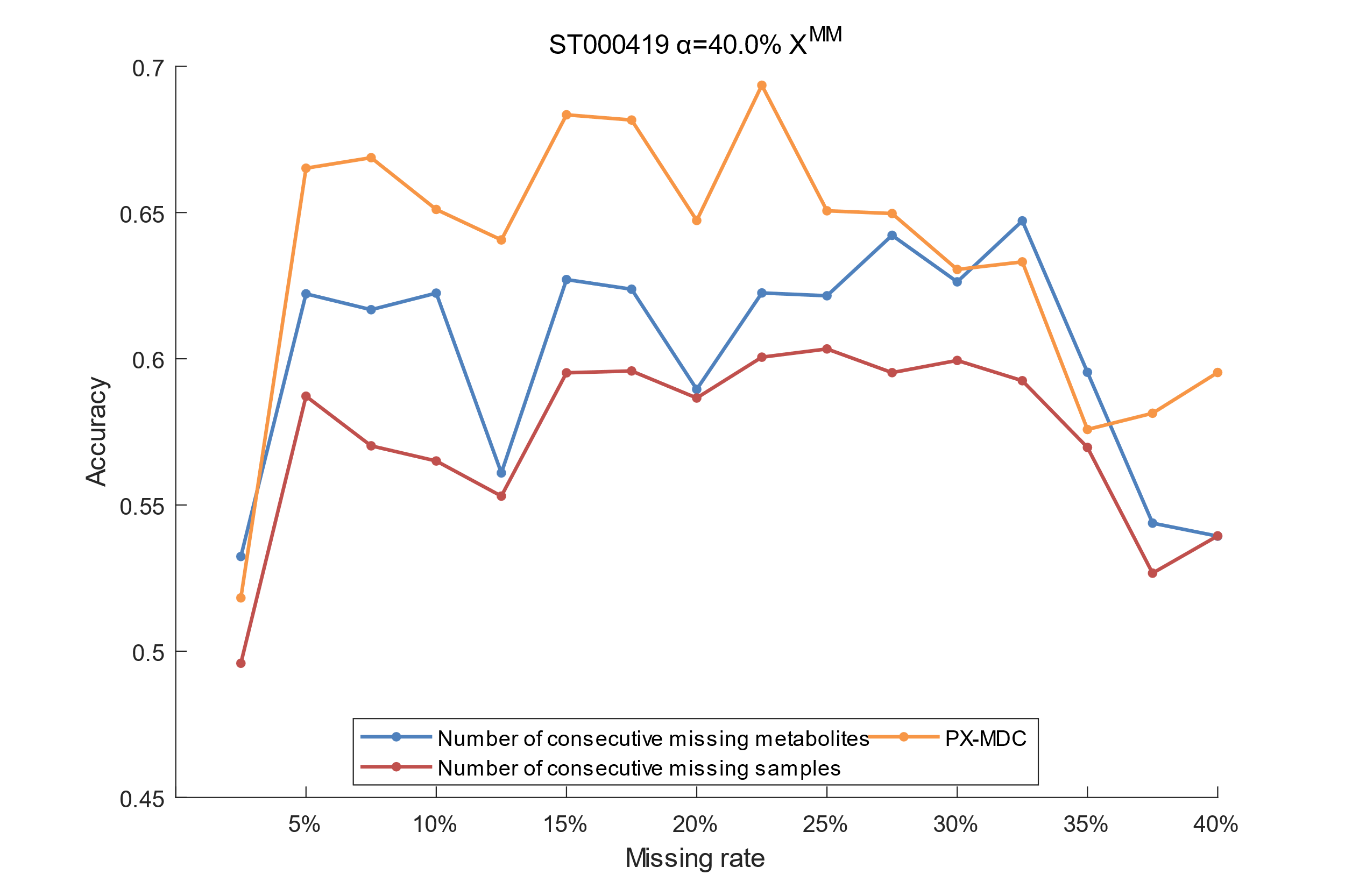 | 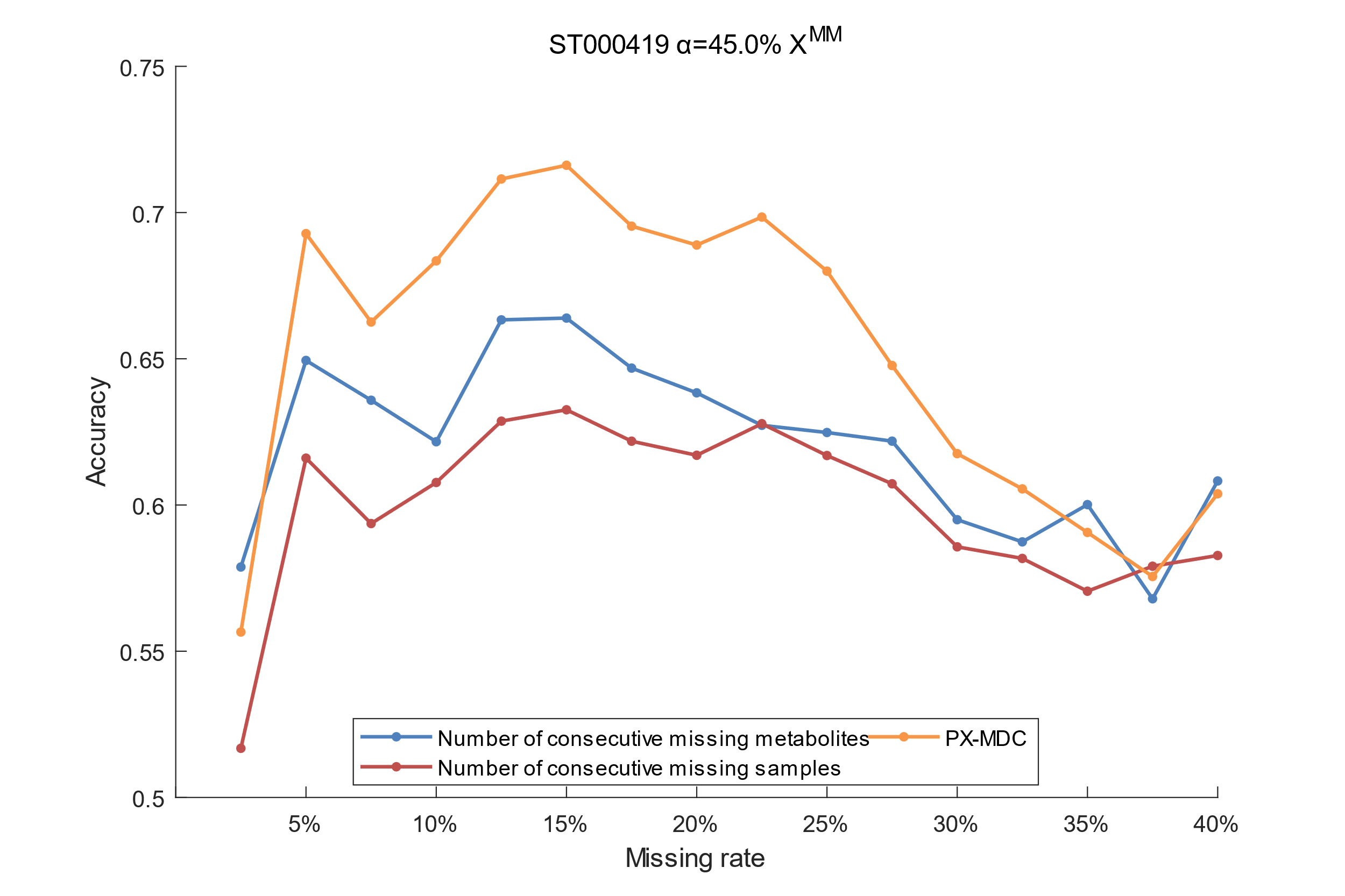 |
| 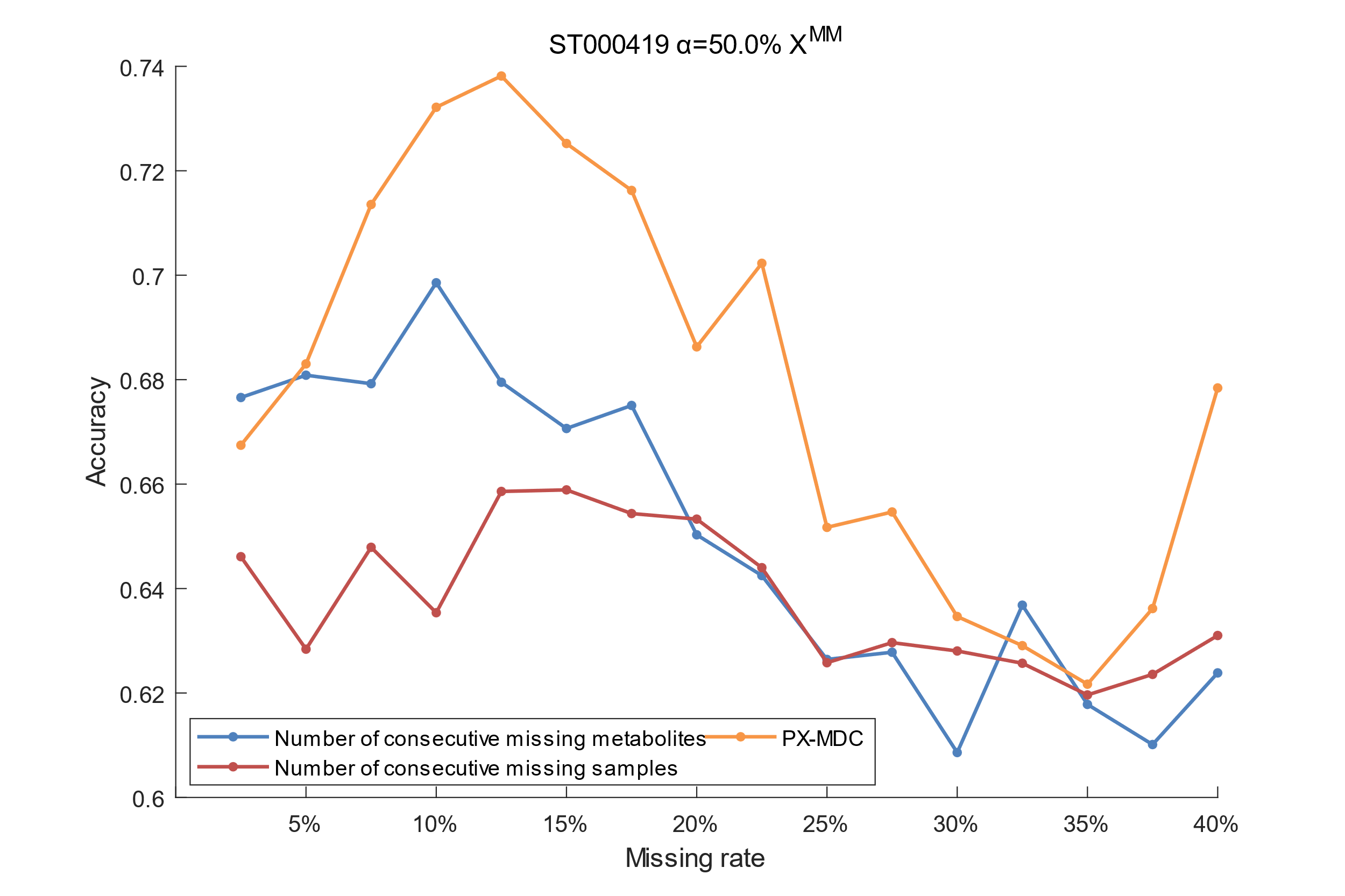 | 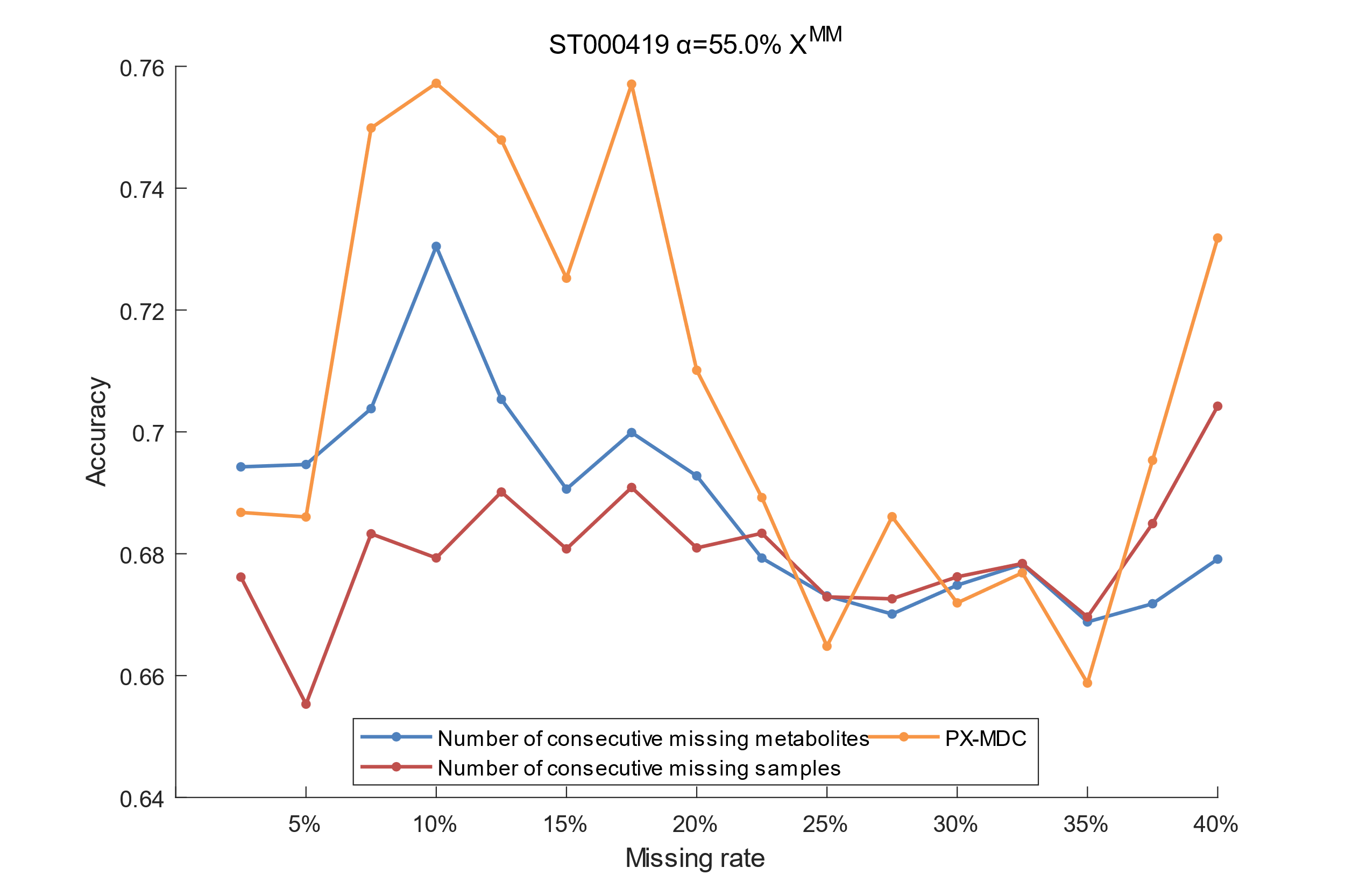 | 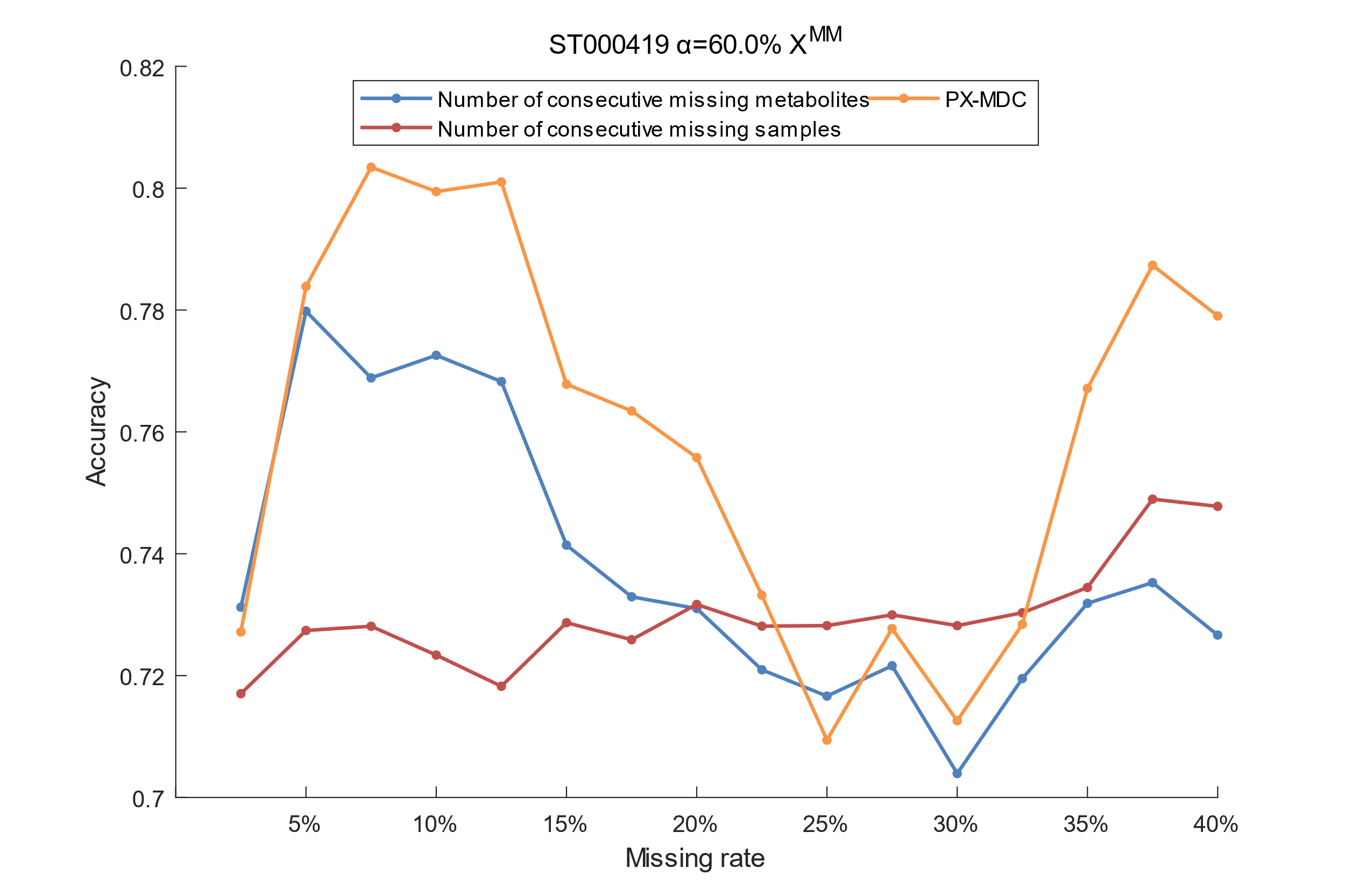 |

**Supplementary Figure 6.** Accuracy of X^MM^ in BP Neural Network model for different features.

| 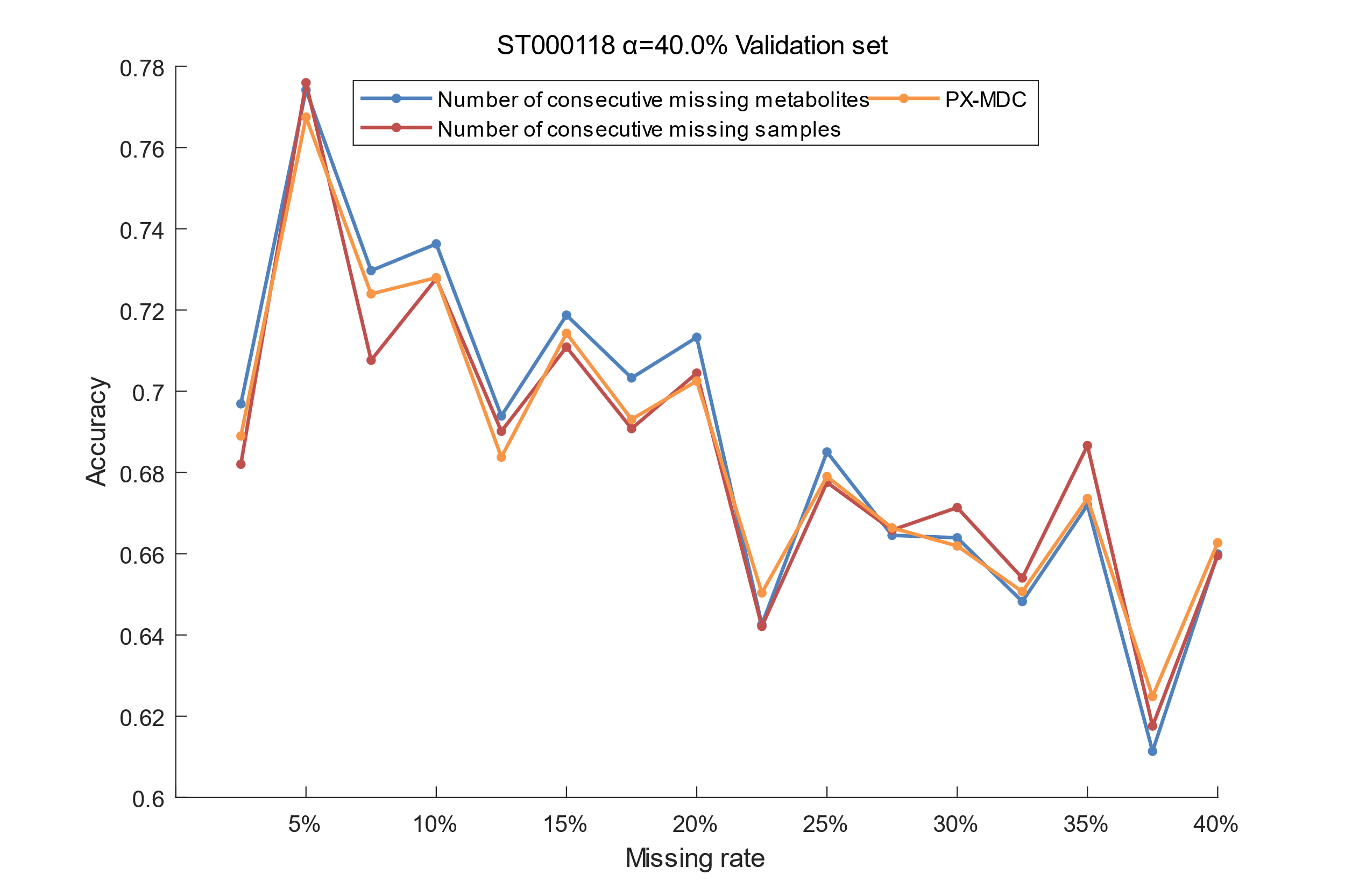 | 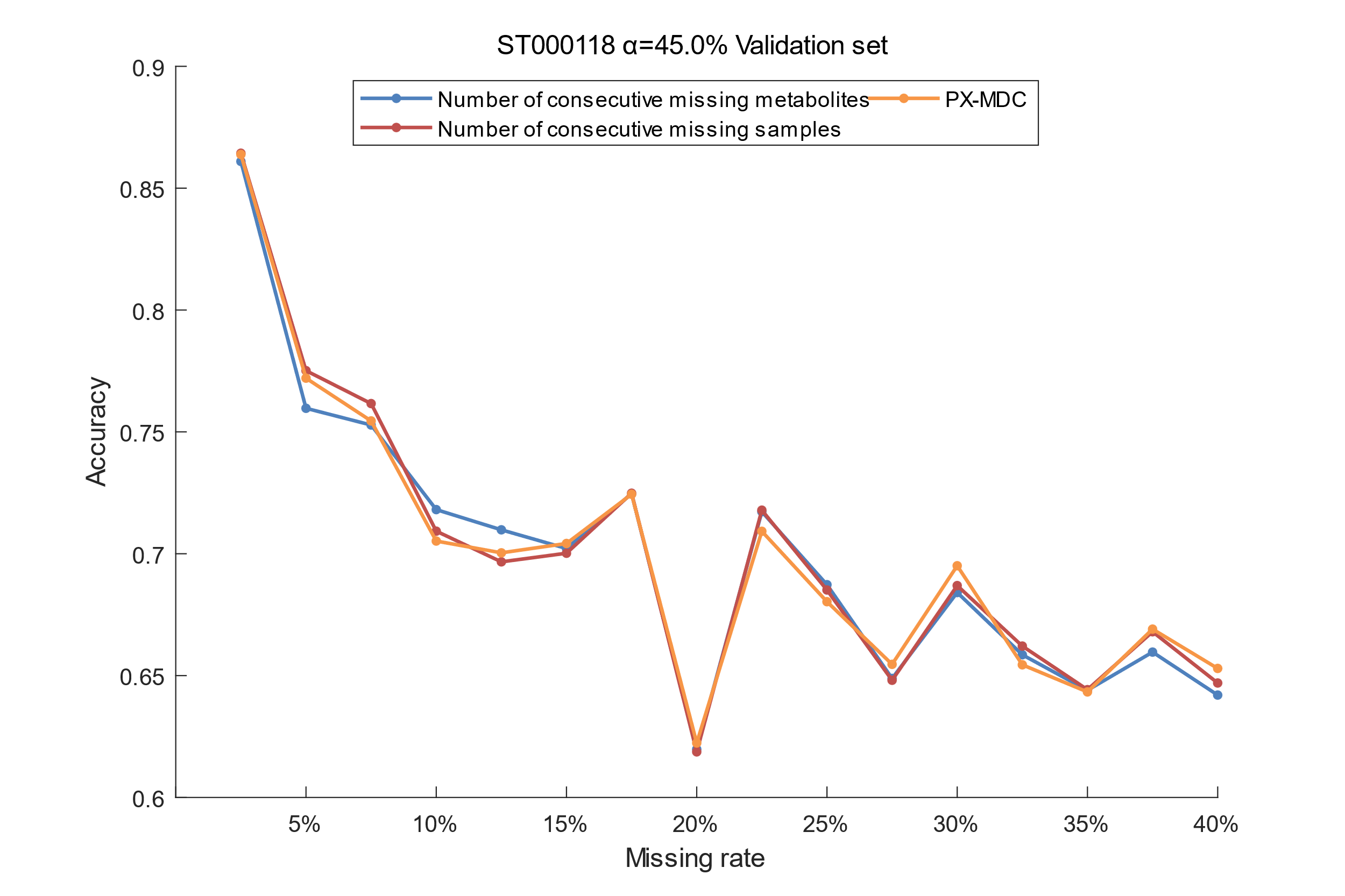 | 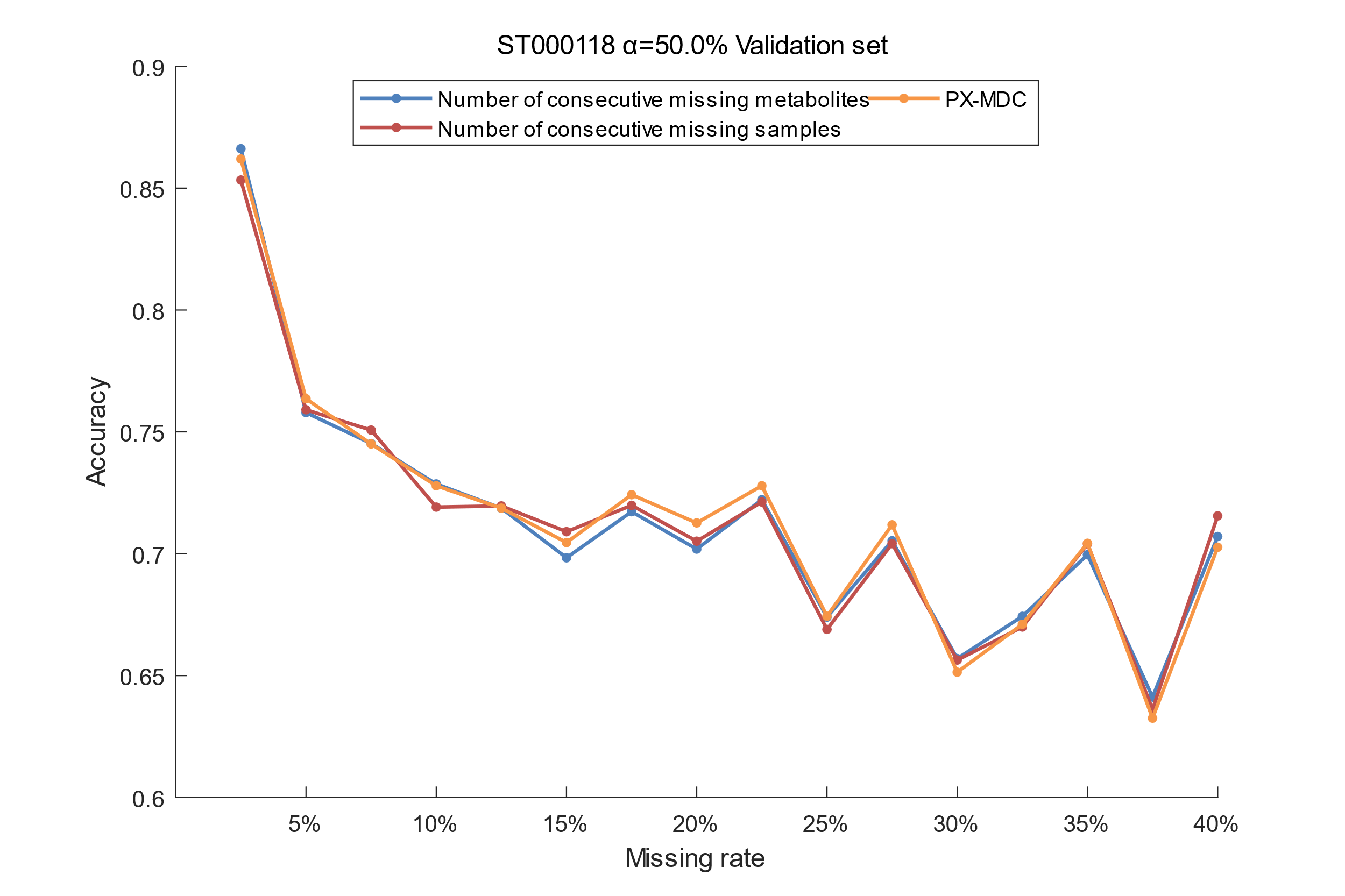 |
| --- | --- | --- |
| 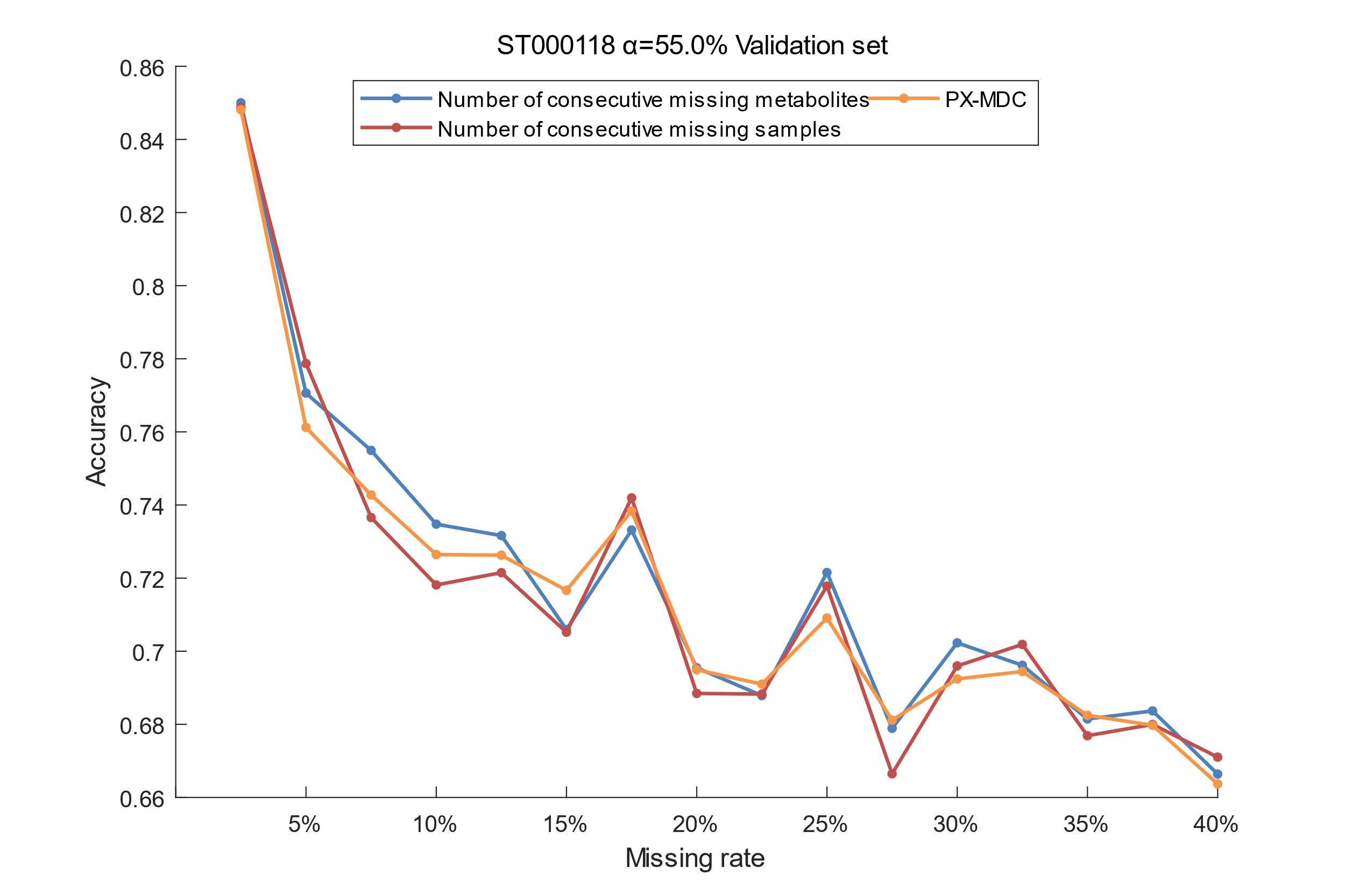 | 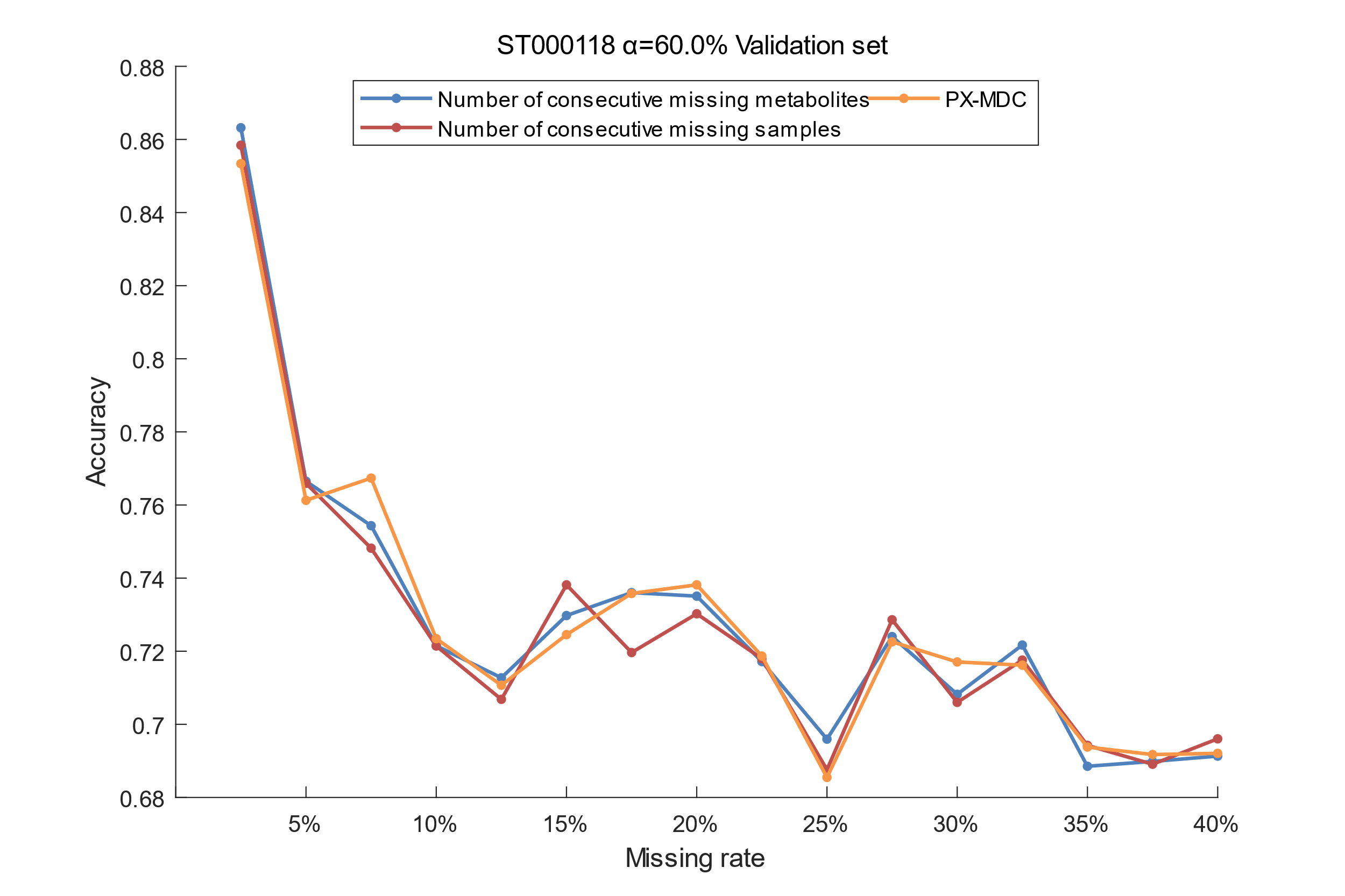 | 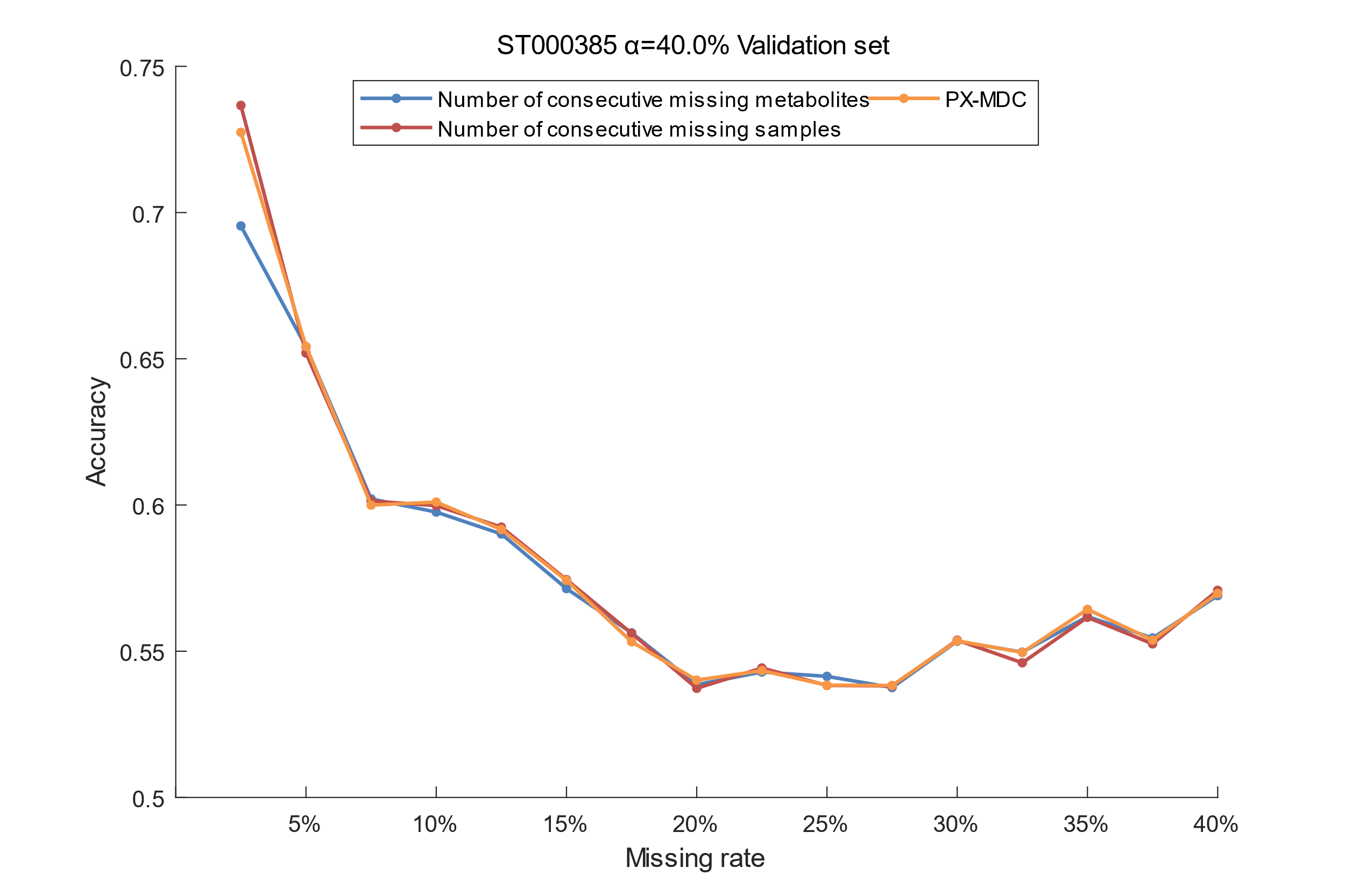 |
| 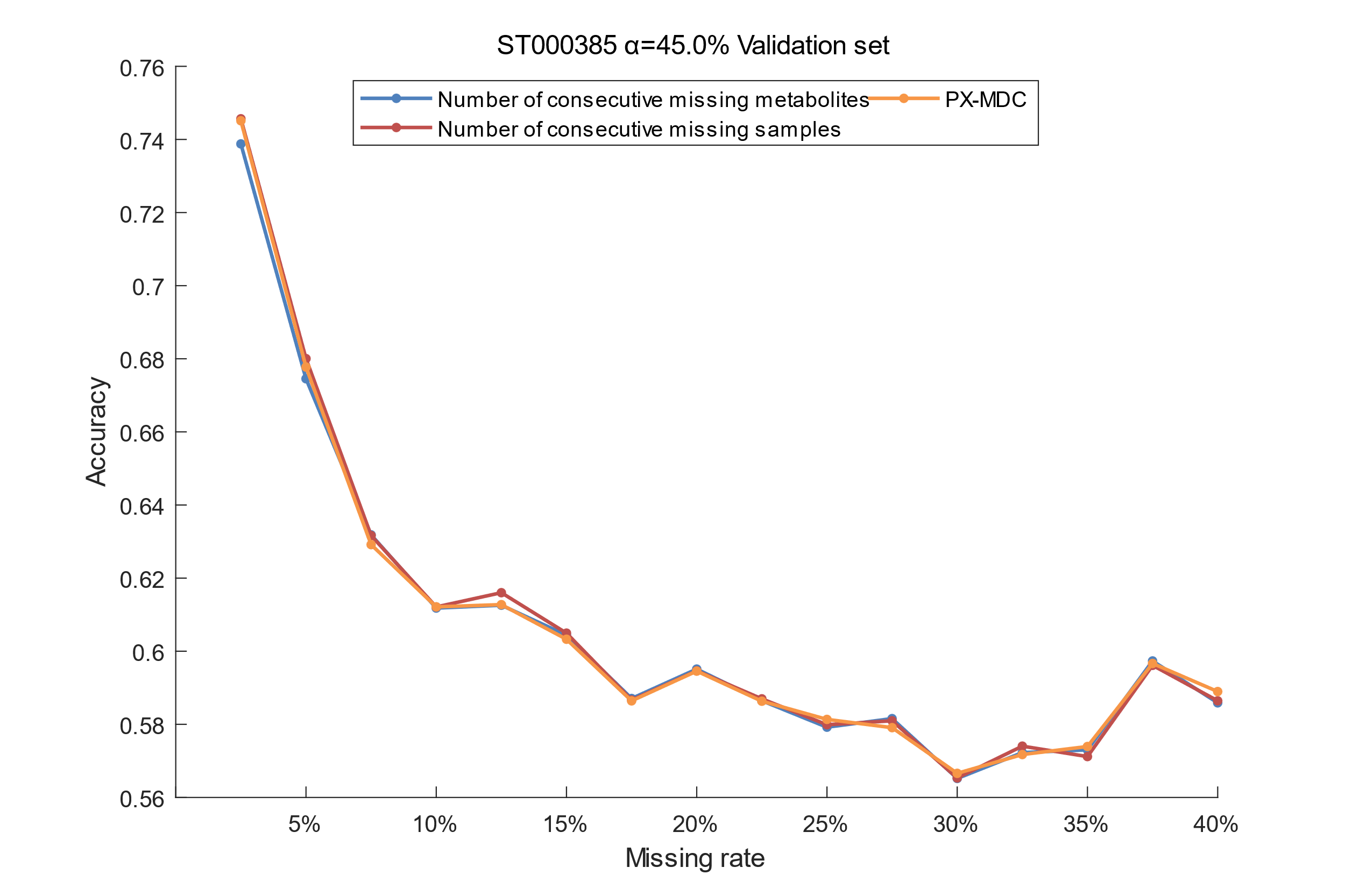 | 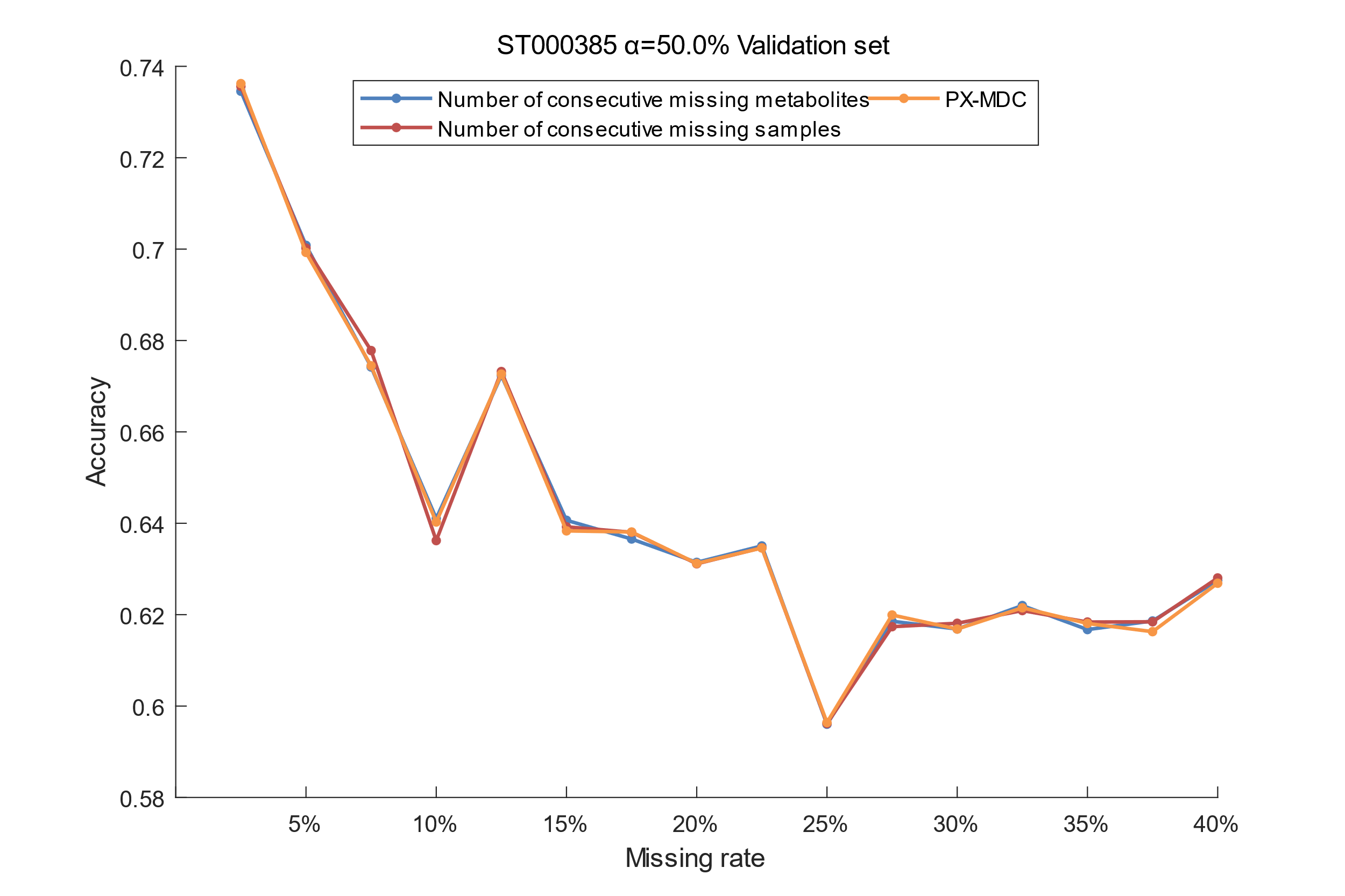 | 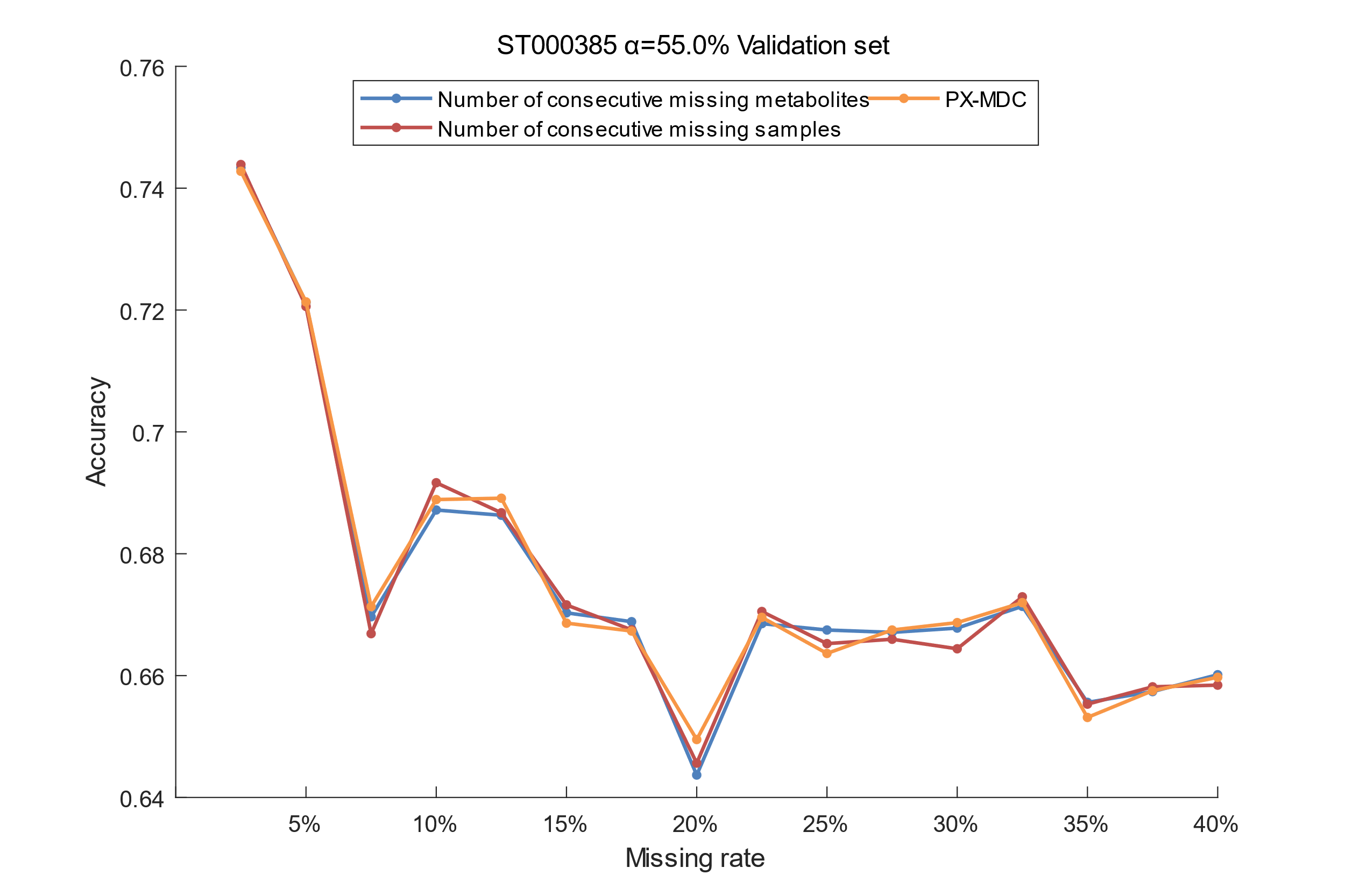 |
| 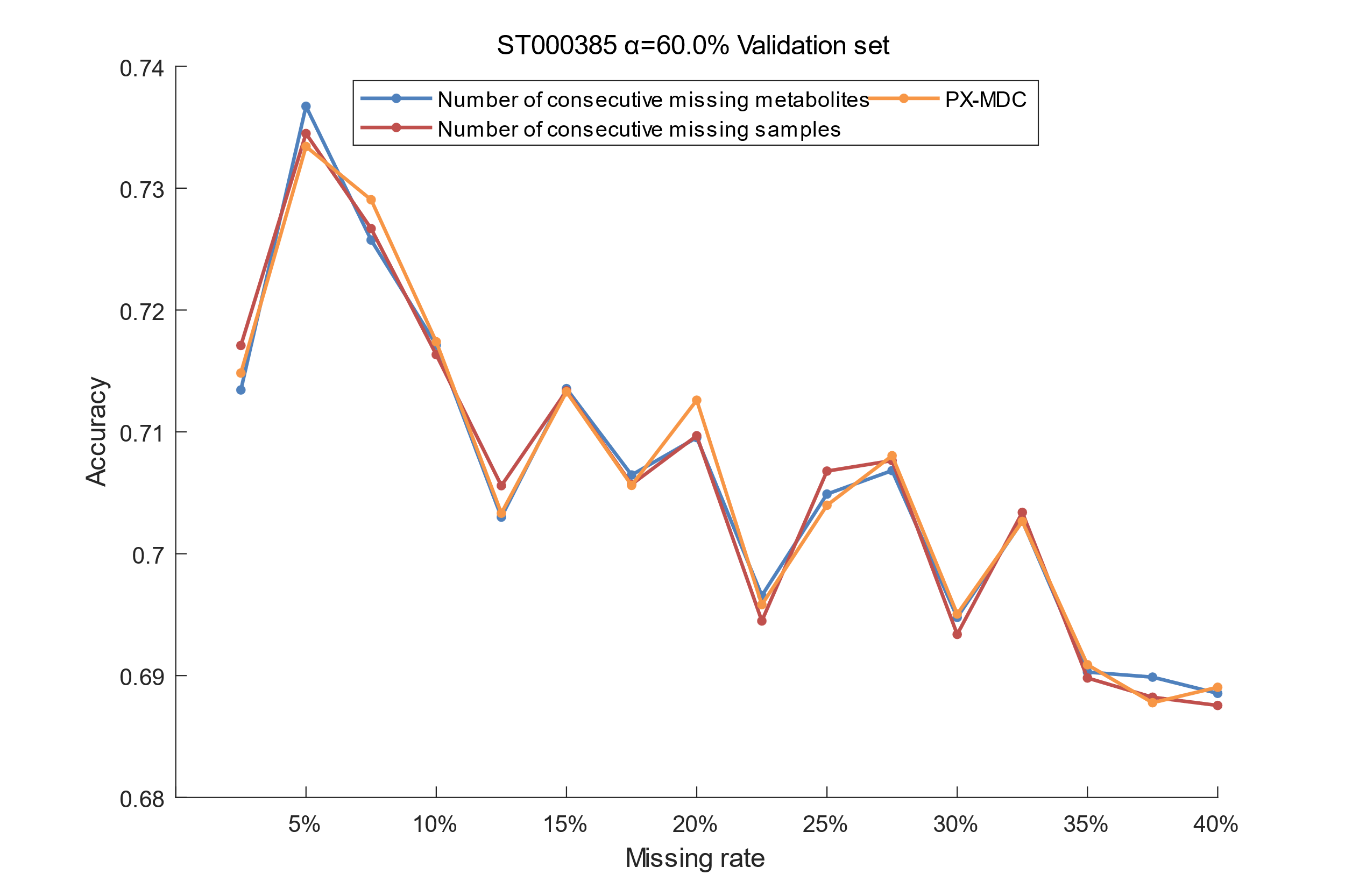 |  |  |
|  |  |  |

**Supplementary Figure 7.** Accuracy of validation sets in Naive Bayes models for different feature.

|  |  |  |
| --- | --- | --- |
|  |  |  |
|  |  |  |
|  |  |  |
|  |  |  |

**Supplementary Figure 8.** Accuracy of X^MM^ in Naive Bayes model for different features.

In Supplementary Figure 9 and Supplementary Figure 10, the prediction accuracies of four different sets of features on XGBoost classifier are compared. Where Supplementary Figure 9 shows the accuracy on the validation set and Supplementary Figure 10 shows the accuracy on X^MM^.

Delete metabolites name was characterized by the number of consecutively missing metabolites, the number of consecutively missing samples, the mean, the median, the minimum, the maximum of each metabolite, the rate of missing metabolites, and the level of metabolite concentration to which it belongs.

Delete metabolites level was characterized by the number of consecutively missing metabolites, the number of consecutively missing samples, the mean, median, minimum, and maximum values for each metabolite, the rate of missing metabolites, and the metabolite name.

Delete metabolites name and level was characterized by the number of consecutive missing metabolites, the number of consecutive missing samples, the mean, median, minimum, maximum, and rate of missing metabolites for each metabolite.

PX-MDC is characterized by the number of consecutive missing metabolites, the number of consecutive missing samples, the mean, the median, the minimum, the maximum of each metabolite, the rate of missing metabolites, the name of the metabolite, the metabolite's concentration to which it belongs.

|  |  |  |
| --- | --- | --- |
|  |  |  |
|  |  |  |
|  |  |  |
|  |  |  |

**Supplementary Figure 9.** Accuracy of validation sets in XGBoost model for different features.

|  |  |  |
| --- | --- | --- |
|  |  |  |
|  |  |  |
|  |  |  |
|  |  |  |

**Supplementary Figure 10.** Accuracy of X^MM^ in XGBoost model for different features.

# Section S2: Tables

In Supplementary Table 1-10, the classification accuracies of the PX-MDC model are compared for three different concentration threshold settings for ST000118, ST000385, and ST000418 data, with a missing rate of 20%, an α from 40% to 60%, and a step size of 5%. Where Supplementary Table 1, Supplementary Table 3, Supplementary Table 5, Supplementary Table 7 and Supplementary Table 9 are the accuracy of the model on the validation set and Supplementary Table 2, Supplementary Table 4, Supplementary Table 6, Supplementary Table 8 and Supplementary Table 10 are the accuracy of the model on the X^MM^ data. Where x is the high concentration threshold, y is the medium concentration threshold and z is the low concentration threshold.

|  | x=0.1_y=0.2_z=0.7 | x=0.25_y=0.45_z=0.3 | x=0.4_y=0.2_z=0.4 |
| --- | --- | --- | --- |
| ST000118 | 0.712230216 | 0.675 | 0.666942675 |
| ST000385 | 0.623704932 | 0.618958236 | 0.634453782 |
| ST000419 | 0.68185654 | 0.711911357 | 0.669230769 |

**Supplementary Table 1.** Accuracy of the model PX-MDC in the validation set when α is 40% and the missing rate is 20%.

|  | x=0.1_y=0.2_z=0.7 | x=0.25_y=0.45_z=0.3 | x=0.4_y=0.2_z=0.4 |
| --- | --- | --- | --- |
| ST000118 | 0.660963245 | 0.647238 | 0.688158 |
| ST000385 | 0.59458071 | 0.618236 | 0.660326 |
| ST000419 | 0.621535858 | 0.633275 | 0.694872 |

**Supplementary Table 2.** Accuracy of model PX-MDC in X^MM^ when α is 40% and missing rate is 20%.

|  | x=0.1_y=0.2_z=0.7 | x=0.25_y=0.45_z=0.3 | x=0.4_y=0.2_z=0.4 |
| --- | --- | --- | --- |
| ST000118 | 0.659217877 | 0.798611111 | 0.6375 |
| ST000385 | 0.607628233 | 0.644558617 | 0.654808959 |
| ST000419 | 0.647727273 | 0.668544601 | 0.696326531 |

**Supplementary Table 3.** Accuracy of the model PX-MDC in the validation set when α is 45% and the missing rate is 20%.

|  | x=0.1_y=0.2_z=0.7 | x=0.25_y=0.45_z=0.3 | x=0.4_y=0.2_z=0.4 |
| --- | --- | --- | --- |
| ST000118 | 0.674822923 | 0.647697595 | 0.627974045 |
| ST000385 | 0.62654253 | 0.636333015 | 0.676375042 |
| ST000419 | 0.740511727 | 0.660227152 | 0.703289684 |

**Supplementary Table 4.** Accuracy of model PX-MDC in X^MM^ when α is 45% and missing rate is 20%.

|  | x=0.1_y=0.2_z=0.7 | x=0.25_y=0.45_z=0.3 | x=0.4_y=0.2_z=0.4 |
| --- | --- | --- | --- |
| ST000118 | 0.682781457 | 0.77037037 | 0.658064516 |
| ST000385 | 0.706140869 | 0.713220676 | 0.695498676 |
| ST000419 | 0.711409396 | 0.680297398 | 0.71105309 |

**Supplementary Table 5.** Accuracy of the model PX-MDC in the validation set when α is 50% and the missing rate is 20%.

|  | x=0.1_y=0.2_z=0.7 | x=0.25_y=0.45_z=0.3 | x=0.4_y=0.2_z=0.4 |
| --- | --- | --- | --- |
| ST000118 | 0.635190575 | 0.669014085 | 0.688157895 |
| ST000385 | 0.653184924 | 0.662940912 | 0.694140013 |
| ST000419 | 0.700417973 | 0.691551232 | 0.723454752 |

**Supplementary Table 6.** Accuracy of model PX-MDC in X^MM^ when α is 50% and missing rate is 20%.

|  | x=0.1_y=0.2_z=0.7 | x=0.25_y=0.45_z=0.3 | x=0.4_y=0.2_z=0.4 |
| --- | --- | --- | --- |
| ST000118 | 0.700729927 | 0.703703704 | 0.755813953 |
| ST000385 | 0.706918239 | 0.701536936 | 0.70904325 |
| ST000419 | 0.662162162 | 0.767137097 | 0.716783217 |

**Supplementary Table 7.** Accuracy of the model PX-MDC in the validation set when α is 55% and the missing rate is 20%.

|  | x=0.1_y=0.2_z=0.7 | x=0.25_y=0.45_z=0.3 | x=0.4_y=0.2_z=0.4 |
| --- | --- | --- | --- |
| ST000118 | 0.68033312 | 0.661589404 | 0.712357955 |
| ST000385 | 0.685512751 | 0.704274162 | 0.731577795 |
| ST000419 | 0.768418874 | 0.668298654 | 0.744648627 |

**Supplementary Table 8.** Accuracy of model PX-MDC in X^MM^ when α is 55% and missing rate is 20%.

|  | x=0.1_y=0.2_z=0.7 | x=0.25_y=0.45_z=0.3 | x=0.4_y=0.2_z=0.4 |
| --- | --- | --- | --- |
| ST000118 | 0.707006369 | 0.720588235 | 0.723529412 |
| ST000385 | 0.635068981 | 0.762512768 | 0.73471223 |
| ST000419 | 0.819121447 | 0.751503006 | 0.780322307 |

**Supplementary Table 9.** Accuracy of the model PX-MDC in the validation set when α is 60% and the missing rate is 20%.

|  | x=0.1_y=0.2_z=0.7 | x=0.25_y=0.45_z=0.3 | x=0.4_y=0.2_z=0.4 |
| --- | --- | --- | --- |
| ST000118 | 0.736680955 | 0.730416373 | 0.782551223 |
| ST000385 | 0.747106637 | 0.748031496 | 0.762355759 |
| ST000419 | 0.733817242 | 0.756066158 | 0.760351967 |

**Supplementary Table 10.** Accuracy of model PX-MDC in X^MM^ when α is 60% and missing rate is 20%.
